# Safety, tolerability, and pharmacokinetics of AL-335 in healthy volunteers and hepatitis C virus-infected subjects

**DOI:** 10.1371/journal.pone.0204974

**Published:** 2018-10-16

**Authors:** Matthew W. McClure, Elina Berliba, Tengiz Tsertsvadze, Adrian Streinu-Cercel, Leen Vijgen, Béatrice Astruc, Alain Patat, Christopher Westland, Sushmita Chanda, Qingling Zhang, Thomas N. Kakuda, Jennifer Vuong, Nick Khorlin, Leonid Beigelman, Lawrence M. Blatt, John Fry

**Affiliations:** 1 Alios BioPharma Inc., part of the Janssen Pharmaceutical Companies, South San Francisco, CA, United States of America; 2 State Medical University “N. Testemitanu” and Arensia Exploratory Medicine, Chisinau, Republic of Moldova; 3 Director General, AIDS and Clinical Immunology Research Center of Georgia, Tbilisi, Georgia; 4 National Institute for Infectious Diseases “Prof. Dr. Matei Balș”, Carol Davila University of Medicine and Pharmacy, Bucharest, Romania; 5 Janssen Research & Development, Janssen Pharmaceutica NV, Beerse, Belgium; 6 Biotrial, Rennes, France; Kaohsiung Medical University, TAIWAN

## Abstract

**Background:**

The nucleotide analog AL-335 is a pangenotypic hepatitis C virus (HCV) nonstructural protein (NS)5B inhibitor being evaluated as treatment for chronic HCV infection.

**Methods:**

This three-part randomized, double-blind study evaluated the safety and pharmacokinetics of single and multiple ascending oral doses of AL-335. Healthy volunteers (HVs) received single doses of AL-335 (100–1,200 mg) or placebo in a fasted or fed (400 mg) state. Non-cirrhotic subjects (HCV genotype [GT]1−4) and GT1-infected subjects with Child Pugh A cirrhosis received multiple doses of AL-335 (400, 800, 1,200 mg) or placebo once daily (QD) for 7 days.

**Results:**

Forty-eight HVs and 64 subjects with HCV GT1−4 were randomized and received treatment. AL-335 was well tolerated in HVs and HCV-infected subjects with/without cirrhosis. AL-335 was rapidly absorbed and converted to the metabolites ALS-022399 and ALS-022227. ALS-022227 exposure increased less than dose-proportionally and was unaffected by food, while AL-335 and ALS-022399 exposure increased with food by 85% and 50%, respectively, in HVs. Rapid and dose-dependent reductions in HCV-RNA were observed in GT1-infected subjects. In non-cirrhotic, GT1−4-infected subjects receiving AL-335 800 mg QD, potent antiviral activity was observed, regardless of genotype (mean maximum reductions in HCV-RNA of 4.0−4.8 log_10_ IU/mL). The same dose in GT1-infected cirrhotic subjects resulted in a 3.5 log_10_ IU/mL mean maximum reduction in HCV-RNA.

**Conclusions:**

AL-335 was well tolerated when administered as single and multiple doses, with an acceptable pharmacokinetic profile. The drug also demonstrated potent antiviral activity in HCV GT1–4-infected subjects, including GT1-infected subjects with cirrhosis.

## Trial registration

NCT02339207; clinicaltrials.gov.

## Introduction

Chronic hepatitis C virus (HCV) infection affects approximately 71 million people worldwide and is associated with severe morbidity/mortality [1]. Fortunately, effective HCV treatment, measured by sustained virologic response, can prevent disease progression and improve both all-cause and liver-specific survival rates [2–5].

The availability of all-oral direct-acting antiviral (DAA) therapies with a variety of mechanisms of action has improved tolerability and led to fewer drug interactions, less frequent dosing, and reduced treatment duration. Improved sustained virologic response rates have also been seen among traditionally ‘difficult-to treat’ subpopulations [6]. Despite these improvements, limitations of current DAA regimens such as the lack of pangenotypic coverage of HCV genotypes (GT), persistent risk of drug-drug interactions (DDI), and the requirement of treatment durations ≥12 weeks have resulted in an ongoing opportunity to improve upon currently available DAA therapies [6, 7].

One promising DAA drug class often associated with a low risk of DDIs, a high barrier to resistance, and pangenotypic coverage is the nucleotide analogs, which includes sofosbuvir [8–10]. These synthetic compounds are transformed *in vivo* from the pharmacologically inactive pro-drug into a biologically active species [8] where they inhibit viral replication via a classic chain termination mechanism [10]. The uridine-based nucleotide monophosphate pro-drug, AL-335, is an investigational HCV nonstructural protein (NS)5B inhibitor that has demonstrated pangenotypic *in vitro* activity with mean 50% effective concentration (EC_50_) values ranging from 0.04 to 0.08 μM for GT1 replicons and GT1, 2, 3, 4, 5, and 6 chimeric replicons. In addition, AL-335 showed a high barrier to resistance, and limited risk of DDI *in vitro* [11, 12]. *In vivo*, AL-335 is rapidly converted to ALS-022399 (monophosphate precursor) by esterases, then subsequently phosphorylated intracellularly. The active moiety of AL-335, ALS-022235 (5’-triphosphate), inhibits HCV NS5B ribonucleic acid (RNA)-dependent RNA polymerase by acting as a chain terminator of RNA synthesis [11]. Furthermore, AL-335 and its metabolites do not interact with host polymerases, including mitochondrial RNA polymerase, the inhibition of which has been linked with mitochondrial toxicity [13]. Dephosphorylation of ALS-022235 yields the parent nucleoside ALS-022227.

The current study, AL-335-601 (clinicaltrials.gov number: NCT02339207), was the first clinical study of AL-335 as a DAA for the treatment of HCV. This study assessed the initial safety and pharmacokinetics of single ascending oral doses (SAD) of AL-335 in healthy volunteers and the safety, pharmacokinetics, and viral effects of multiple ascending oral doses (MAD) of AL-335 in treatment-naïve cirrhotic or non-cirrhotic subjects with chronic HCV GT1−6 infection.

## Methods

### Study objectives and design

**Fig 1:**
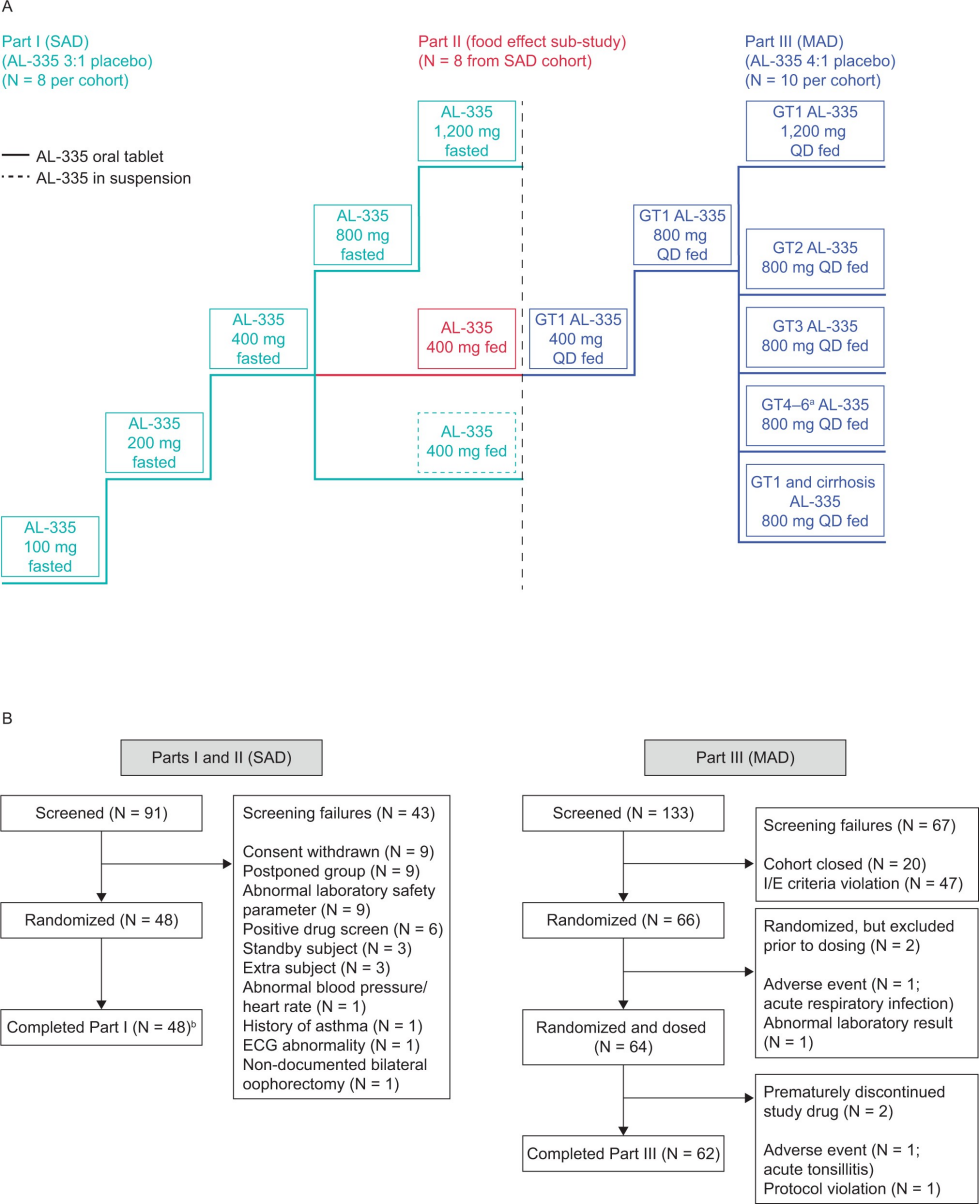
**(A) Study design and (B) subject disposition.** ^a^All randomized subjects in the GT4–6 cohort were infected with GT4; ^b^8/48 subjects (from AL-335 400 mg tablet cohort) were enrolled in Part I and then re-dosed in fed state. ECG = electrocardiogram, fasted = in fasted state, fed = in fed state, GT = genotype, I/E = inclusion/exclusion, MAD = multiple ascending doses, QD = once daily, SAD = single ascending doses.

This randomized, double-blind, placebo-controlled study comprised three parts (Fig 1A). Part I (SAD) was conducted in healthy volunteers and evaluated the safety, tolerability, and pharmacokinetics of five single doses of AL-335 (100, 200, 400, 800, and 1,200 mg) in a fasted state, on Day 1. All doses were administered as oral tablets; a single 400 mg dose as an oral suspension was also assessed in the fed state. Eight healthy volunteers were randomized in a 3:1 ratio (AL-335:placebo) per cohort, and were monitored through 8 days.

Part II assessed the impact of a high-fat breakfast on the pharmacokinetics of AL-335 in eight healthy volunteers previously given AL-335 400 mg tablet or placebo in the fasted state in Part I. In Part II, 11–21 days after the first dose, volunteers received a single dose of AL-335 400 mg following a high-fat breakfast, and were subsequently monitored for a further 8 days.

In Part III (MAD), safety, pharmacokinetics, HCV-RNA kinetics, and the emergence of amino acid changes in the HCV NS5B gene were evaluated following administration of seven once-daily (QD) oral doses of AL-335 tablets (400 mg, 800 mg, or 1,200 mg) through 8 consecutive days, to HCV GT1−6-infected subjects without cirrhosis, and to HCV GT1-infected subjects with Child Pugh A cirrhosis, in a fed state. Up to 10 subjects were randomized in a 4:1 ratio (AL-335:placebo) per MAD cohort. Subjects were monitored through 21 days from first dose in Part III.

Parts I and II were completed at a clinical pharmacology unit in France, and Part III was conducted at four sites in France, Georgia, Moldova, and Romania. The study protocol, and all amendments, were approved in all enrolling countries by appropriate national health authorities and local ethics committees, and met the principles of the Declaration of Helsinki and Good Clinical Practice guidelines. The protocol was amended three times during the course of the study. The main reasons included: addition of a broader range of subjects with chronic HCV infection (GT2, GT3, GT4−6, and those with compensated cirrhosis); addition of a cohort to evaluate an oral suspension of AL-335 compared with a tablet formulation; increase in the sample size to allow for additional sites and cohorts of subjects; adjustments to the schedule of events and patient management guidelines; and provision of updated information on the clinical experience for prior cohorts. All subjects provided informed, written consent to participate.

The study protocol and amendments (Substantial Modification document [SM] n°1, SM2 and SM3), the subject information sheet and the informed consent were submitted to and approved before the start of the study by:

For France: The Independent Ethics Committee (IEC): Comité de Protection des Personnes (CPP) Ouest VI, Centre Hospitalier Universitaire Cavale Blanche, Avenue Tanguy Prigent, 29609 Brest Cedex, France; and the Medecines Agency (MA): Agence Nationale de Sécurité du Médicament et des produits de santé (ANSM).

For Georgia: The Local Ethics Committee (LEC) and, only after approval by the LEC, the Health Authority (HA): Ministry of Labour, Health and social Affairs of Georgia, Legal Entity of Public Law, State Regulation Agency for Medial Activities, 144 Ak. Tsereteli ave., 0159 Tbilisi, Georgia.

For Moldova: The National Ethics Committee (NEC): Clinical Research of Medicines and New Methods of Treatment; the MA: Agency of Medicines and Medical Devices, 2/1 Korolenko Street, MD-2028, Chisinau, Republic of Moldova; and the Ministry of Health of the Republic of Moldova.

For Romania: The NEC: Academy of Medical Sciences, National Bioethics Committee of Medicines and Medical Devices, 19–21, Sos. Stefan cel Mare street, District 2, Bucharest, Romania; and the MA: Ministry of Health, National Agency of Medicines and Medical Devices, 48 Av. Sanatescu Street, District 1, 011478 Bucharest, Romania.

### Study populations

For a detailed listing of the entry criteria for each part of the study, please refer to the S1 Appendix, and the study protocol, which can be found in S2 Appendix.

#### Parts I−III

Male or female healthy volunteers or subjects were required to be in good general health, based on a thorough medical evaluation that included medical history, physical examination, laboratory tests, and an electrocardiogram (ECG). Women of child-bearing potential were not eligible for enrollment into the study.

Subjects in Parts I−III were excluded if they had a positive pre-study drugs test, including for amphetamines, barbiturates, cocaine, opiates, cannabinoids (HCV-infected subjects in Part III could be included if they had a positive result for cannabinoids at screening but abstained from cannabinoid use throughout the study), phencyclidine, and benzodiazepines.

#### Additional part-specific entry criteria

**Parts I and II**. The date of the first subject enrollment was December 6, 2014, and the date of the last completed subject was June 26, 2015.

**Part III**. Subjects were HCV-infected, treatment-naїve (to DAA treatment), without cirrhosis (except for one cirrhosis cohort), and aged between 18 and 65 years. Subjects were enrolled according to HCV genotype: GT1, 2, 3, or 4−6. BMI was within the range 18–35 kg/m^2^ inclusive (minimum weight 50 kg). All subjects required documentation of HCV infection for greater than 6 months at the time of study drug administration (HCV-RNA ≥10^5^ IU/mL for subjects without cirrhosis or ≥10^4^ and ≤10^8^ IU/mL for subjects with cirrhosis). To be eligible for the GT1 with compensated cirrhosis cohort, subjects were required to meet the Child Pugh Class A definition plus either a recent (<6 months) liver biopsy result indicating the presence of cirrhosis, or a recent (<3 months) FibroScan liver stiffness score ≥14.5 kPa. Liver ultrasound concerning for malignancy was exclusionary. The date of the first subject enrollment was January 28, 2015, and the date of the last completed subject was May 3, 2016.

### Study assessments

Details of safety and pharmacokinetic assessments for Parts I–III and details of pharmacodynamic assessments for Part III (MAD) can be found in the protocol in S2 Appendix.

Briefly, adverse events (AEs) were assessed continuously while subjects were confined to the clinic, and at each outpatient visit. Safety laboratory tests, including hematology, biochemistry, and urinalysis, were assessed every 1–5 days while confined, then periodically at outpatient visits. ECGs were collected daily while confined, then periodically at outpatient visits. Vital signs and physical examinations were conducted at regular intervals throughout the study. Blood samples for HCV-RNA quantification (using COBAS Ampliprep/COBAS TaqMan HCV Test Version 2.0 [Roche]) and for viral sequencing (using Sanger population sequencing of the HCV NS5B gene) were collected daily while confined and at each follow-up visit. HCV genotyping was performed at screening using the VERSANT HCV Genotype 2.0 Assay (LiPA, Siemens Healthcare). Urine (SAD; 800 mg fasted cohort only) and plasma pharmacokinetic samples were collected over the first 48 hours in Parts I and II, and Day 1 and Day 7 in Part III. Additional pharmacokinetic samples were collected beyond 48 hours after a dose. Plasma and urine concentrations of study medication and metabolites were then assessed by a liquid chromatography tandem mass spectrometry method and used to calculate the values of various pharmacokinetic parameters.

### Study drug dosing–fed or fasted state

In Part I, healthy volunteers and subjects fasted for at least 8 hours overnight until 4 hours post-dose, except for those receiving AL-335 400 mg as an oral suspension, who were dosed in the fed state. For those fasting, water was freely available except from 1 hour prior to dosing until 2 hours post-dosing.

In Part II, healthy volunteers were given a standardized high-fat-content breakfast (consisting of two eggs fried in butter, two strips of bacon, two slices of toast with butter, four ounces of hash brown potatoes [fried with butter] and 8 ounces [240 mL] of whole milk) [14] 30 minutes before dosing. Consumption of grapefruit or grapefruit-containing products was prohibited during the study.

In Part III, HCV GT1−6-infected subjects were dosed in the fed state.

### Statistical analyses

See S1 Appendix for further details of the statistical analyses, including pharmacokinetics.

Given the small sample size in each cohort, all assessments were summarized descriptively. Continuous variables were summarized by number of subjects, mean, standard deviation, median, and range. Categorical data were summarized by the number and percentage of subjects.

Pharmacokinetic data were analyzed using Phoenix WinNonLin version 6.3 (Certara USA Inc., Princeton, NJ, USA). Safety and pharmacokinetic data were summarized using SAS software (9.2 release or higher; SAS Institute Inc., Cary, NC, USA). Relevant pharmacokinetic parameters (e.g. area under the concentration-time curve from time zero to the last sample with measurable plasma concentration [AUC_0–last_], maximum measured drug concentration [C_max_], time of maximum concentration, and apparent terminal elimination half-life [t_½_]) were calculated by standard non-compartmental methods for those subjects with sufficient plasma (or urine, Part I only) concentration data.

For analysis of food effects (Part II only), an ANOVA was performed using log-transformed data for C_max_ and AUC_0–last_ for AL-335, ALS-022399, and ALS-022227 with log_(dose)_ as fixed effect and subject as random effect. Geometric mean ratios and corresponding 90% confidence interval (CI) were presented. Comparable exposures between the food conditions or formulation was concluded if the 90% CI for C_max_ and AUC_0–last_ fell within the range of 80.00‒125.00%.

Summary statistics were calculated from log-transformed HCV-RNA values, and data were presented in terms of the absolute value over time and the mean change from baseline up to Day 21, with baseline HCV-RNA defined as the average of Day -2 and Day 1 pre-dose values. AEs were coded according to the Medical Dictionary for Regulatory Activities (MedDRA; Version 17.1).

## Results

### Study population disposition and baseline characteristics

The disposition of screened and randomized subjects is defined in Fig 1. In total, 91 healthy volunteers were screened and 48 were randomized and completed Part I of the study. Among these, eight subjects who received a fasted 400 mg tablet dose of AL-335 or placebo in Part I continued for a second period of single-dose administration in the fed state in Part II. For Part III, a total of 133 HCV GT1-, 2-, 3-, or 4−6-infected subjects were screened, and 66 subjects were randomized. Of these, 64 subjects were dosed and 62 (97%) subjects completed the study.

Subject baseline demographics and disease characteristics for each cohort in Parts I−II are presented in Table 1, and Part III are presented in Table 2. In general, there were no clinically significant imbalances between comparison groups in all three Parts. The majority of enrolled subjects were male (88%) and white (97%), with a mean age of 31.0−55.5 years and BMI of 22.9−32.5 kg/m^2^. In Part III, the mean baseline HCV-RNA levels ranged from 5.0 to 6.5 log_10_ IU/mL.

**Table 1:** Subject baseline demographics and disease characteristics in Parts I and II; SAD and food effect sub-study.

**PART I** **SAD**	**Placebo fasted**	**AL-335 100 mg tablet fasted**	**AL-335 200 mg tablet fasted**	**AL-335 400 mg tablet fasted**	**AL-335 400 mg oral suspension fed**	**AL-335 800 mg tablet fasted**	**AL-335 1,200 mg tablet fasted**	**Overall**
N	12	6	6	6	6	6	6	48
Age (years), mean (SD)	35.8 (13.2)	43.2 (5.9)	38.5 (10.0)	45.2 (9.0)	31.3 (11.4)	31.0 (10.4)	36.5 (15.9)	37.2 (11.8)
Male, n (%)	10 (83.3)	5 (83.3)	6 (100)	5 (83.3)	6 (100)	6 (100)	6 (100)	44 (91.7)
Race, n (%)								
White	12 (100)	6 (100)	6 (100)	6 (100)	4 (66.7)	6 (100)	6 (100)	46 (95.8)
Black/African American	0	0	0	0	1 (16.7)	0	0	1 (2.1)
Mixed race	0	0	0	0	1 (16.7)	0	0	1 (2.1)
BMI (kg/m^2^), mean (SD)	24.0 (2.0)	23.8 (2.7)	23.7 (3.5)	25.7 (2.3)	23.7 (3.7)	22.9 (3.3)	24.3 (2.8)	24.0 (2.7)
**PART II** **Food effect sub-study**	**Placebo fed**			**AL-335 400 mg tablet fed**				**Overall**
N	2			6				8
Age (years), mean (SD)	39.0 (18.4)			45.2 (9.0)				43.6 (10.7)
Male, n (%)	1 (50.0)			5 (83.3)				6 (75.0)
Race, n (%)								
White	2 (100)			6 (100)				8 (100)
Ethnicity, n (%)								
Not Hispanic or Latino	2 (100)			6 (100)				8 (100)
BMI (kg/m^2^), mean (SD)	25.5 (0.1)			25.7 (2.3)				25.7 (1.9)

BMI = body mass index, SAD = single ascending doses, SD = standard deviation.

**Table 2:** Subject baseline demographics and disease characteristics in Part III; MAD.

PART III MAD	HCV GT1				HCV GT1 + comp cirrhosis		HCV GT2		HCV GT3		HCV GT4–6^a^		Overall
	Placebo fed	AL-335 400 mg fed	AL-335 800 mg fed	AL-335 1,200 mg fed	Placebo fed	AL-335 800 mg fed	Placebo fed	AL-335 800 mg fed	Placebo fed	AL-335 800 mg fed	Placebo fed	AL-335 800 mg fed	
N	6	8	8	8	2	8	2	8	2	8	1	3	64
Age (years), mean (SD)	41.7 (9.8)	38.6 (15.0)	38.3 (11.2)	37.9 (12.1)	55.5 (2.1)	48.4 (13.4)	38.0 (2.8)	41.8 (11.4)	44.0 (5.7)	38.9 (4.1)	48.0 (N/A)	46.3 (8.6)	41.6 (11.0)
Male, n (%)	6 (100)	5 (62.5)	7 (87.5)	7 (87.5)	2 (100)	5 (62.5)	1 (50.0)	8 (100)	2 (100)	8 (100)	1 (100)	2 (66.7)	54 (84.4)
Race, n (%)													
White	6 (100)	8 (100)	8 (100)	8 (100)	2 (100)	8 (100)	2 (100)	8 (100)	2 (100)	8 (100)	1 (100)	2 (66.7)	63 (98.4)
Black/African American	0	0	0	0	0	0	0	0	0	0	0	1 (33.3)	1 (1.6)
Ethnicity, n (%)													
Not Hispanic or Latino	6 (100)	8 (100)	8 (100)	8 (100)	2 (100)	8 (100)	2 (100)	8 (100)	2 (100)	8 (100)	1 (100)	3 (100)	64 (100)
BMI (kg/m^2^), mean (SD)	25.3 (3.0)	28.1 (3.7)	28.9 (4.1)	24.7 (2.6)	27.1 (1.4)	24.3 (4.5)	32.5 (2.9)	26.9 (3.4)	24.4 (1.9)	24.7 (3.4)	23.1 (N/A)	27.1 (3.6)	26.3 (3.8)
Mean HCV viral load (log_10_ IU/mL)	5.8	6.2	6.3	5.9	5.8	5.9	6.2	6.5	5.0	6.3	6.3	5.9	N/A

^a^All randomized subjects in the HCV GT4–6 cohort were infected with GT4.

BMI = body mass index, comp = compensated, GT = genotype, HCV = hepatitis C v”us, MAD = multiple ascending doses, N/A = not available, SD = standard deviation.

Among the 64 subjects that were dosed with AL-335 in Part III, 40 were infected with HCV GT1 (including 10 subjects with Child Pugh A cirrhosis), 10 with GT2, 10 with GT3, and four with GT4. No HCV GT5- or 6-infected subjects enrolled in the GT4−6 cohort, which did not fill by the end of the study, at which point four subjects had been randomized (three AL-335: one placebo).

Among the 10 subjects randomized to the GT2 cohort, a retrospective analysis of genotype based on NS5B Sanger sequencing demonstrated that eight subjects in this cohort were infected with a GT2/1b recombinant virus, with the virus’ NS5B gene being of GT1b. The other two subjects were infected with full-length HCV GT2, with one infected with subtype 2c (who received placebo) and one being subtype 2k (who received active treatment).

### Safety

In Parts I and II, no serious AEs were reported. Two treatment-emergent AEs (TEAEs) were reported during Part I: a mild feeling of palpitations (AL-335 100 mg) and a moderate toothache (two occasions in the same subject) (AL-335 200 mg), neither of which were considered related to study drug. No TEAEs were reported during Part II. No clinically significant changes in laboratory parameters, vital signs, ECG, or physical examinations were recorded in Part I or Part II.

In Part III, no serious AEs were reported and only one TEAE (acute tonsillitis in a placebo-treated subject) resulted in study drug discontinuation. The overall rate of TEAEs reported in subjects following MAD of AL-335 was 29.4% (n = 15, reporting a total of 21 TEAEs) for subjects receiving AL-335 (all doses) compared with 53.8% (n = 7, reporting a total of 14 TEAEs) for subjects receiving placebo. No TEAEs occurred in a dose-responsive manner, nor did any occur in a clinically important imbalance in active versus placebo groups. The most commonly reported TEAEs among subjects treated with AL-335 were headache (n = 7, reporting seven TEAEs), nasopharyngitis (n = 2, reporting two TEAEs), and increased alanine transaminase/aspartate transaminase (n = 2, each reporting elevations of alanine transaminase/aspartate transaminase). These elevations were similar in magnitude to baseline values and occurred at the Day 17 completion visit (concurrent with the subjects’ HCV-RNA levels returning to baseline). All TEAEs reported were mild or moderate in severity except one (elevated creatine phosphokinase in a non-cirrhotic HCV GT1-infected subject treated with AL-335 800 mg), which was severe in intensity and considered unrelated to the study drug because it was associated with antecedent strenuous physical activity with improvement despite continued dosing. A complete list of all reported TEAEs is presented by treatment group in Table A in S1 Appendix.

Grade 3 biochemistry laboratory abnormalities were detected in a total of nine subjects during Part III (one AL-335 400 mg QD-, six AL-335 800 mg QD-, and two placebo-treated subjects). None of these resulted in premature discontinuation of the study drug. They included one case of elevated creatinine phosphokinase (described above) and one case of elevated bilirubin in a subject treated with placebo. All other Grade 3 biochemistry laboratory abnormalities (elevated cholesterol [n = 3], glucose [n = 1], and calcium levels [n = 1], and low phosphate levels [n = 2]) were not considered to be clinically significant. No clinically significant changes in vital signs, ECG, or physical examinations were observed.

### Pharmacokinetics

#### Parts I and II (SAD and food effect sub-study)

**Fig 2:**
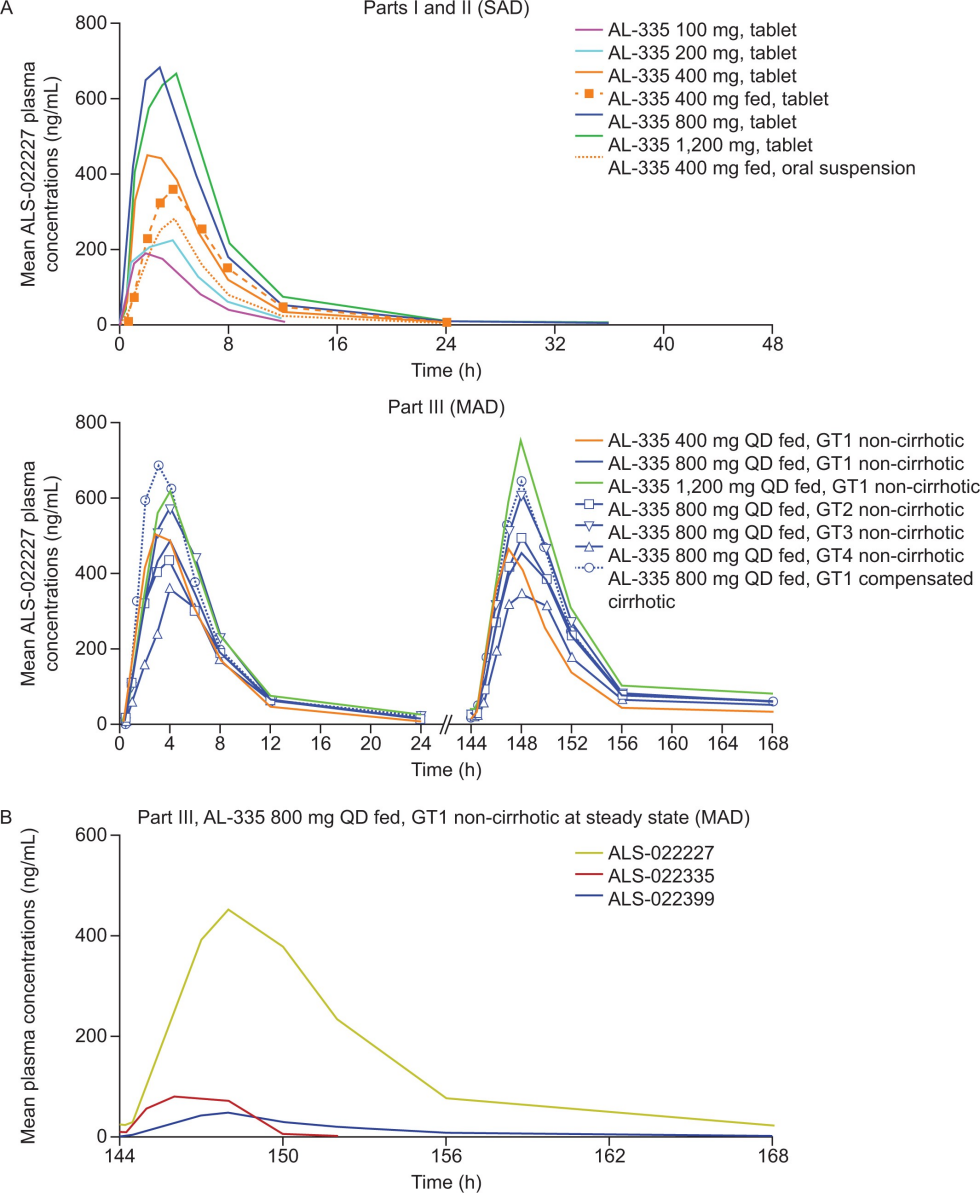
**(A) Mean plasma concentration of ALS-022227 after SAD in Part I/II (top) and after MAD in Part III on Days 1 and 7 (bottom) in healthy volunteers; (B) Mean plasma concentration of AL-335, ALS-022399, and ALS-022227 after multiple doses of AL-335 administered as 800 mg QD in HCV GT1-infected subjects without cirrhosis (Part III, cohort 2).** fed = in fed state, GT = genotype, HCV = hepatitis C virus, MAD = multiple ascending doses, QD = once daily, SAD = single ascending doses.

The mean plasma pharmacokinetic parameters of AL-335, ALS-022399, and ALS-022227 following a single dose of AL-335 are presented in Table B in S1 Appendix, and the plasma concentration–time profile of ALS-022227 is presented in Fig 2A. When administered under fasted conditions, AL-335 was rapidly absorbed and metabolized to ALS-022399 and ALS-022227, as indicated by the short half-life of AL-335 (mean t_½_ ≤1 h for all doses) and relatively short time of maximum concentration of ALS-022399 (median 1–2.5 h) and ALS-022227 (median 2.5–4 h).

Following dosing with AL-335, the C_max_ and AUC_0–last_ of AL-335, ALS-022399, and ALS-022227 increased in a less than dose-proportional manner with the exception of ALS-022399, for which a close to dose-proportional increase in AUC_0–last_ was observed for increasing doses of AL-335.

The administration of AL-335 (400 mg) under fed conditions resulted in increased exposure of AL-335 and ALS-022399 (increases in AUC_0–last_ of 85% and 50%, respectively) but not ALS-022227 (mean AL-335, ALS-022399, and ALS-022227 AUC_0–last_ values of 76, 117, and 2,512 ng.h/mL, respectively, under fasted conditions and 140, 175, and 2,425 ng.h/mL, respectively, in the fed state). The effect of food on the pharmacokinetics of AL-335 and its metabolites was not considered clinically significant.

Exposure (C_max_ and AUC_0–last_) to AL-335 and its metabolites increased significantly following administration of the tablet formulation of AL-335 compared with the same dose of an oral suspension (400 mg fed). For AL-335, C_max_ and AUC_0–last_ were both approximately 80% higher following tablet administration compared with oral suspension, and a similar trend was observed for ALS-022399 (76% and 66% higher, respectively) and ALS-022227 (52% and 83% higher, respectively). No significant difference in time of maximum concentration was observed for any analytes between formulations.

Pharmacokinetic analysis of urine following single administration of AL-335 800 mg under fasted conditions revealed that AL-335 was mainly recovered in urine as the parent nucleoside ALS-022227 (13.5%), and only 0.142% and 0.446% of the total dose was recovered as AL-335 and ALS-022399, respectively.

#### Part III (MAD)

Plasma pharmacokinetic parameters of AL-335, ALS-022399, and ALS-022227 following multiple ascending doses of AL-335 (Days 1–7) are presented in Table C in S1 Appendix and the plasma concentration-time profile of ALS-022227 is presented in Fig 2A. AL-335 was less rapidly absorbed in HCV-infected subjects than in healthy volunteers, but the same rapid conversion of AL-335 to ALS-022399 and ALS-022227 was observed.

In subjects with HCV GT1 infection without cirrhosis, peak AL-335 and ALS-022399 plasma concentrations and exposure to AL-335 and ALS-022399 increased in a generally dose-proportional or close to dose-proportional manner following the first dose. By Day 7 of AL-335 administration, these parameters were generally slightly lower (~20%) compared with Day 1, with some exceptions for individual doses. A less than dose-proportional increase in C_max_ and AUC_0−last_ parameters was observed for ALS-022227 following repeated doses.

Following repeated administration of AL-335 800 mg, there were no clinically relevant pharmacokinetic differences across HCV GT1–4 subtypes with respect to AL-335, ALS-022399, and ALS-022227, given the small sample sizes and variability. A representative mean plasma concentration-time profile of AL-335, ALS-022399, and ALS-022227 at steady-state is presented in Fig 2B. In HCV GT1-infected subjects with compensated cirrhosis, there was an increase in C_max_ and AUC_0–last_ for AL-335 (up to ~2.1- and ~1.5-fold increase on Day 1 and Day 7, respectively) and ALS-022399 (up to ~1.6- and ~1.3-fold increase on Day 1 and Day 7, respectively), compared with HCV GT1-infected subjects without compensated cirrhosis. Additionally, ALS-022227 C_max_ was increased (~1.4-fold on Day 1 and Day 7) in subjects with compensated cirrhosis, while similar exposures were observed.

In general, no significant accumulation of exposure (AUC_0–24h_ and C_max_) was observed between Day 1 and Day 7 for the three analytes (t_½_ 0.6–0.8 h) in any of the cohorts assessed in Part III.

### Pharmacodynamic outcomes

**Fig 3:**
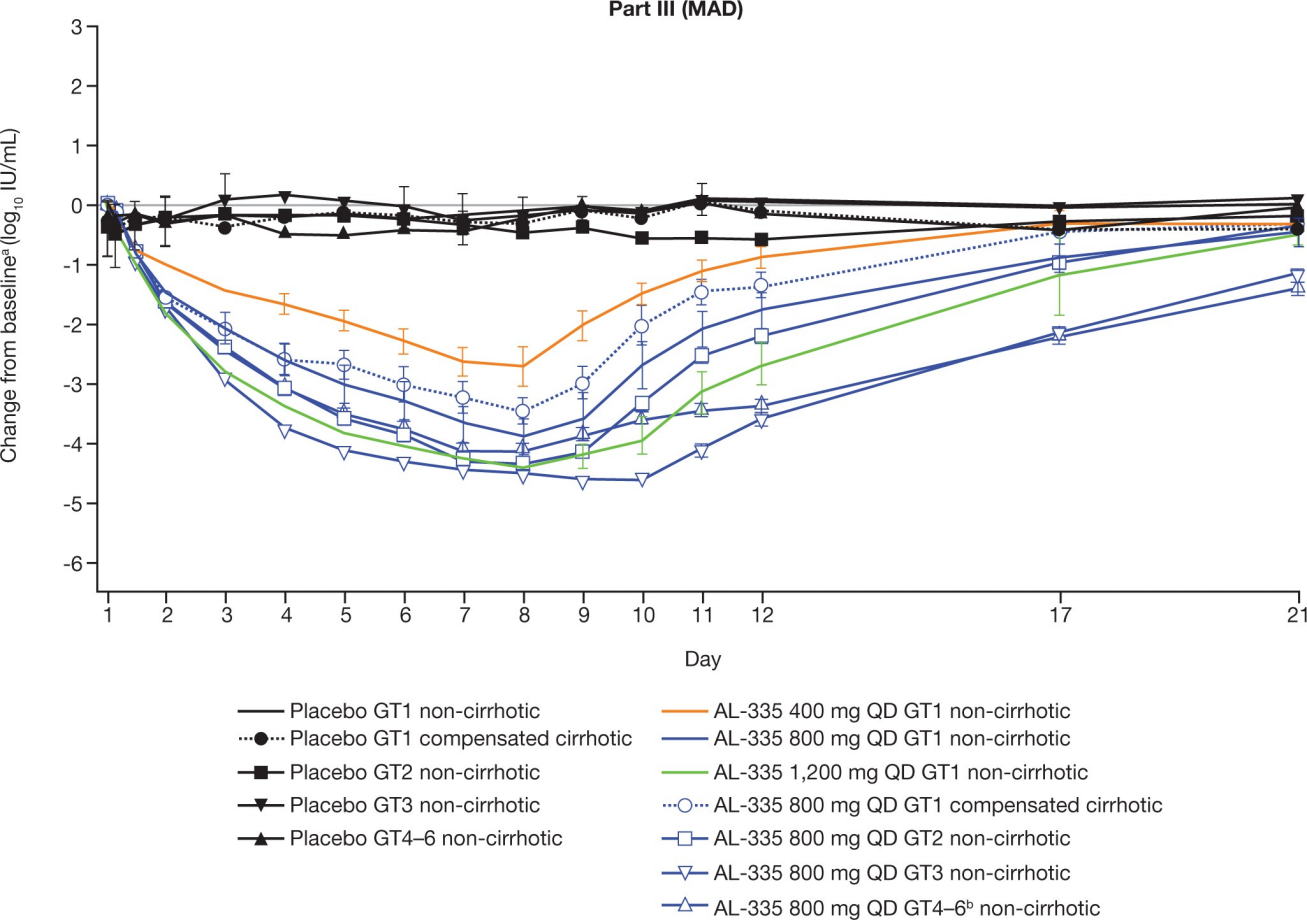
Mean (±SEM) HCV-RNA change from baseline (log_10_ IU/mL) over time following multiple oral administrations of AL-335 (tablet form) under fed conditions (Part III, Days 1–7; pharmacodynamic set). Values below the LLOQ (<15 IU/mL) and with HCV-RNA detected have been replaced by the LLOQ value (= 15 IU/mL). Not detected values have been replaced by 10 IU/mL. ^a^Baseline is defined as the average of Day -2 and Day 1 pre-dose values; ^b^All randomized subjects in the GT4–6 cohort were infected with GT4. GT = genotype, HCV = hepatitis C virus, LLOQ = lower limit of quantification, MAD = multiple ascending doses, QD = once daily, RNA = ribonucleic acid, SEM = standard error of the mean.

**Fig 4:**
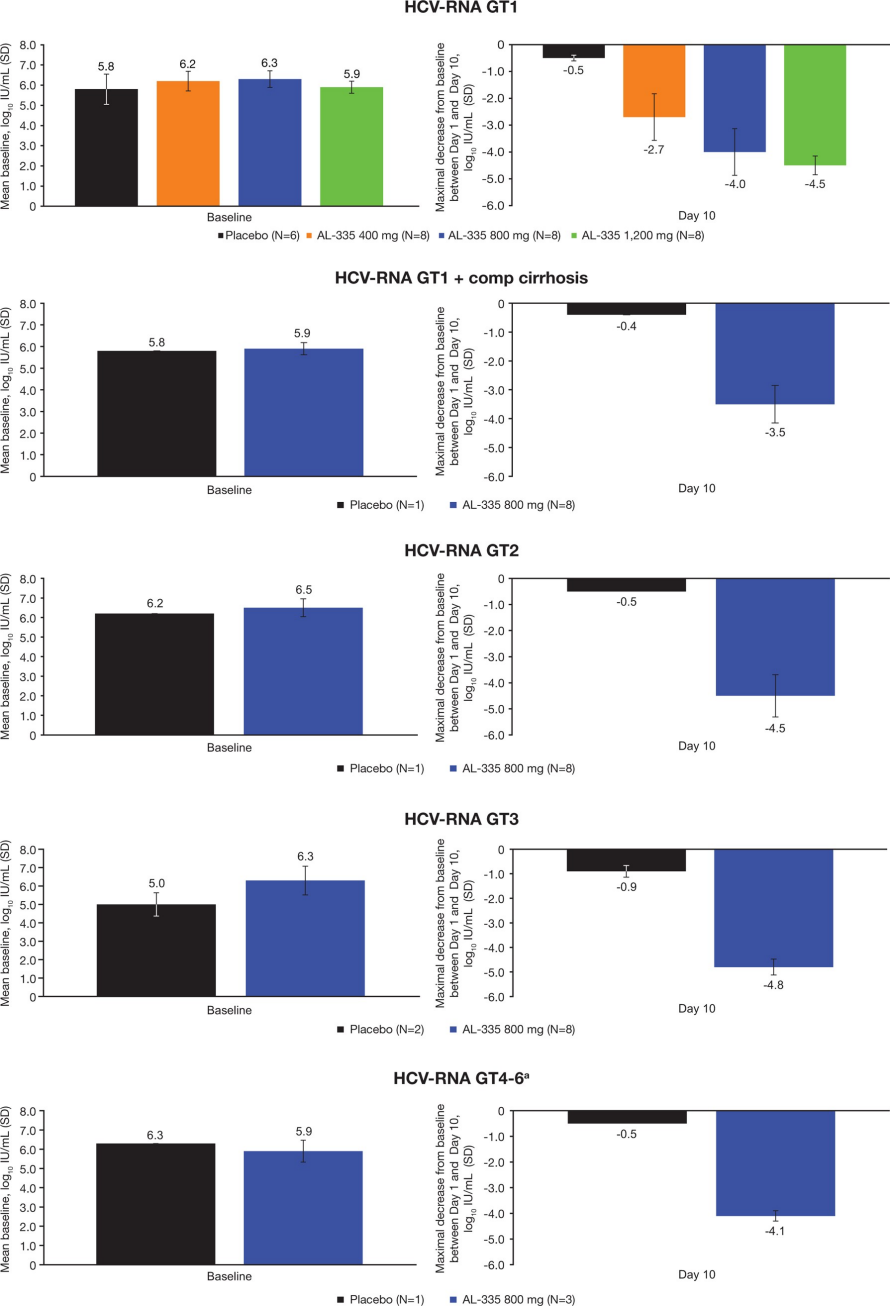
Mean maximal change from baseline in HCV-RNA per cohort between Day 1 and Day 10 in Part III. ^a^All randomized subjects in the HCV GT4–6 cohort were infected with GT4. Baseline is defined as the average of Day -2 and Day 1 pre-dose values. comp = compensated, GT = genotype, HCV = hepatitis C virus, RNA = ribonucleic acid, SD = standard deviation.

In subjects with GT1 infection without cirrhosis, a rapid, sustained dose-dependent reduction in mean HCV-RNA levels was observed following multiple doses (Fig 3), with mean maximal reductions from baseline in HCV-RNA of 2.7, 4.0, and 4.5 log_10_ IU/mL for the AL-335 400 mg, 800 mg, and 1,200 mg doses, respectively. A similar antiviral response was observed in subjects receiving AL-335 800 mg regardless of HCV genotype, with mean maximal reductions in HCV-RNA from baseline of 4.0, 4.5, 4.8, and 4.1 log_10_ IU/mL for GT1-, GT2-, GT3-, and GT4-infected subjects, respectively (Fig 4). Of note, the majority of subjects (N = 8) in the GT2 cohort who received AL-335 were infected with a recombinant virus with an NS5B gene derived from GT1b. In the one subject infected with full-length HCV GT2 (subtype 2k) who received AL-335, the maximum reduction in HCV-RNA from baseline was 5.15 log_10_ IU/mL. In GT1-infected subjects with compensated cirrhosis, AL-335 800 mg resulted in a mean maximal reduction of 3.5 log_10_ IU/mL. In contrast, the mean maximal reduction in HCV-RNA among subjects administered placebo was minimal (0.9 log_10_ IU/mL). Notably, no cases of on-treatment viral breakthrough were observed in any subjects receiving AL-335. During the follow-up period, HCV-RNA levels returned to pre-treatment levels for all subjects.

The NS5B S282T amino acid substitution, associated with AL-335 *in vitro* resistance, was not observed by Sanger population sequencing in any of the subjects, either at baseline or emerging at the end of dosing or at the end of follow-up (Day 21). In one GT1b-infected subject (without cirrhosis), who received AL-335 800 mg, an emerging NS5B C316Y (in a mixture with reference amino acid C316) was observed at the end of dosing (Day 7) but was no longer observed at the end of follow-up (Day 21). The C316Y substitution did not reduce the activity of AL-335 when tested as a site directed mutant in a GT1 transient replicon assay (data on file).

## Discussion

This study demonstrated that AL-335 doses up to 1,200 mg for durations of up to 7 days were well tolerated in healthy volunteers (single doses) and HCV GT1−4-infected subjects with or without Child Pugh A cirrhosis (GT1 only).

No safety signals were identified in the study, which suggested that further investigation of AL-335 in HCV-infected subjects at the doses evaluated in this study, including for longer treatment durations, is warranted.

The pharmacokinetic profile of AL-335 following SAD and MAD in healthy volunteers or HCV-infected subjects showed that the prodrug AL-335 was rapidly converted, as indicated by the quick appearance of the parent nucleoside (ALS-022227). Unfortunately, the active moiety ALS-022235 could not be directly measured in this study as liver biopsy would be required and due to the technical challenges of intracellular (intrahepatocyte) bioanalysis [15]. ALS-022227 is a product of ALS-022235 dephosphorylation; therefore, plasma concentrations of ALS-022227 are an indication that activation occurred. This is further supported by the substantial decline in HCV-RNA observed in the HCV-infected subjects.

Exposure to AL-335 and its metabolites following oral administration was higher when administered as a tablet versus an oral suspension. The effect of food on exposure to the parent nucleoside, ALS-022227, was not considered clinically significant, despite increased exposure to AL-335 and ALS-022399 under fed conditions. Future clinical trials of AL-335 will use the tablet formulation which will be administered without regard to meals.

Following single oral administration, AL-335 was mainly recovered in the urine as the parent nucleoside, ALS-022227. Following multiple ascending doses of AL-335 over 7 consecutive days, there was no apparent accumulation of AL-335 or metabolites. The pharmacokinetics of AL-335, ALS-022399, and ALS-022227 were similar for all subjects regardless of HCV genotype, and the presence of compensated cirrhosis in HCV GT1-infected subjects was associated with increased exposure to AL-335, ALS-022399, and ALS-022227. The pharmacokinetic data from this study support QD dosing of AL-335 irrespective of HCV genotype in subjects with or without compensated cirrhosis.

The rapid, sustained, dose-dependent reduction in mean HCV-RNA levels and similar levels of antiviral activity, regardless of genotype, indicate that AL-335 has pangenotypic antiviral activity, at least across the genotypes evaluated. The antiviral response seen in the 800 and 1,200 mg groups is comparable to what has been demonstrated with sofosbuvir monotherapy over 7 days [16]. Furthermore, the activity observed in GT1-infected subjects with compensated cirrhosis indicates that AL-335 800 mg may be sufficient as part of a combination DAA regimen to treat all subjects regardless of cirrhosis and HCV genotype status. One caveat should be acknowledged, however, with regards to the data derived from the GT2 cohort. Only one of these AL-335-treated subjects had a full-length GT2 virus, while the other seven subjects were infected with a recombinant GT2/1b virus with an NS5B gene derived from GT1b. The crossover point in a recombinant 2k/1b virus which is common in Georgia, where the majority of these subjects were recruited, has been mapped within the NS2 genomic region, with the viral genome part at the 5’ end of the crossover point being of GT2k origin and that at the 3’ end of the crossover point, including the NS5B polymerase, being of GT1b origin [17, 18]. As such, the activity observed for AL-335 for these recombinant viruses may more accurately reflect the activity of AL-335 against the GT1b polymerase. Nevertheless, in light of the substantial antiviral activity (5.15 log_10_ IU/mL maximum reduction) observed in the one subject with a full-length GT2 (2k) virus and the known antiviral activity observed *in vitro* against GT2 [11], the authors still argue that the data generated in this study indicate AL-335 has genotypic coverage for the genotypes studied (i.e. GT1−4).

The NS5B S282T amino acid substitution, associated with AL-335 *in vitro* resistance, was not observed by population sequencing in any of the subjects with sequencing data available either at baseline or emerging at the end of dosing or at end of follow-up. This is encouraging given the significant impact that resistance-associated substitutions can have on the clinical management of HCV-infected subjects. This finding is in line with the high genetic barrier for the development of resistance in subjects for the class of nucleotide analogs [19].

There were several limitations of this study. Primarily it is important to note the limited sample size of the groups studied, and as such, these data should be interpreted with caution. A further limitation was the absence of HCV GT5- and GT6-infected subjects from the HCV GT4–6 cohort. Given that AL-335 demonstrated potent and consistent antiviral activity against HCV GT1–6 *in vitro* and in HCV GT1−4-infected subjects *in vivo* in this study, similar activity of AL-335 in HCV GT5- and GT6-infected subjects may be expected. Another limitation is the short treatment duration and the monotherapy design. However, it was critical that the safety, pharmacokinetics, and antiviral activity of AL-335 be characterized as a monotherapy prior to being combined with other anti-HCV agents as a potential curative therapy.

In conclusion, AL-335 doses up to 1,200 mg for durations of up to 7 days appear to be generally safe and well tolerated, with a favorable pharmacokinetic profile regardless of HCV genotype or cirrhosis. AL-335 monotherapy in subjects without cirrhosis resulted in rapid, potent, and prolonged reductions in HCV-RNA with mean maximum reductions from baseline in HCV-RNA ≥4.0 log_10_ IU/mL in subjects infected with HCV GT1, 2, 3, and 4. In HCV GT1-infected subjects with Child Pugh A cirrhosis, HCV-RNA reductions displayed a similar pattern, albeit at a slightly lower magnitude.

## Supporting information

S1 Appendix (DOCX)

S2 Appendix (PDF)

S1 Checklist (DOCX)
