# Supplementary material for: Safety, tolerability, and pharmacokinetics of AL-335 in healthy volunteers and hepatitis C virus-infected subjects
Source: PLoS One. 2018 Oct 16;13(10):e0204974. doi: 10.1371/journal.pone.0204974 (PMC6191080; doi:10.1371/journal.pone.0204974)
Supplement: S1 Appendix — (DOCX) [file pone.0204974.s001.docx]

**S1 Appendix**

**Study objectives and design**

The primary objective of this study was to evaluate the safety and tolerability of single doses of AL-335 administered to healthy volunteers and multiple doses of AL-335 administered to hepatitis C virus (HCV)-infected subjects. Secondary objectives included: the characterization of the pharmacokinetics of AL-335 and its metabolites (ALS-022399 and ALS-022227) in plasma and urine; the effect of formulation (tablet versus suspension) and food intake on the pharmacokinetics of AL-335; and the HCV-ribonucleic acid (RNA) viral kinetics and viral resistance profile after multiple doses of AL-335.

**Study populations**

**Parts I−III**

Subjects with clinically significant medical illness or psychiatric disorder, or clinically significant abnormal electrocardiogram or laboratory findings, were excluded from study participation. Subjects testing positive for hepatitis A virus immunoglobulin, hepatitis B surface antigen antibody, or HIV antibody were also excluded. Any subject reporting regular alcohol intake (>7 or >14 units per week for females and males, respectively) within 3 months of the screening visit was ineligible for study enrollment.

**Additional part-specific entry criteria**

**Parts I and II**

Subjects were healthy volunteers aged between 18 and 60 years and with a body mass index (BMI) of 18–32 kg/m^2^ inclusive (minimum weight 50 kg). No more than 25% of subjects in any cohort could be enrolled with a BMI ≥30 kg/m^2^. Subjects were excluded if they had a history of regular tobacco use (i.e. ≥10 cigarettes per day) or had used nicotine-containing products within 3 months of the screening visit.

**Study assessments**

**Safety assessments (Parts I–III)**

All subjects underwent a complete physical examination at screening and at study completion. Clinical laboratory variables (including serum chemistry, hematology, and urinalysis) were assessed at screening and throughout the study (Days -2, 2, 3, and 8 [Parts I and II]; Days -2, 2, 7, 9, 12, and 21 [Part III]). Vital signs (body temperature, respiratory rate, supine blood pressure, and heart rate) and 12-lead electrocardiogram measurements were recorded at regular intervals throughout. Adverse events (AEs) were monitored throughout the duration of the study. An AE was considered treatment-emergent if it occurred at the same time as or after receiving the first/only dose of study drug.

**Pharmacokinetic assessments (Parts I–III)**

Samples for pharmacokinetic analysis were collected throughout the study (Days 1–6 and 8 [Parts I and II] and Days 1–12 and 21 [Part III]). Pharmacokinetic determinations were based on plasma concentrations of AL-335 (pro-drug), ALS-022399 (monophosphate precursor), and ALS-022227 (parent nucleoside) following single (Parts I and II) and multiple (Part III, Days 1–7) oral doses of AL-335. The following parameters were obtained using non-compartmental analysis: maximum measured drug concentration (C_max_), time of maximum concentration, apparent terminal elimination half-life, and area under the concentration-time curve from time zero to the last sample with measurable plasma concentration (AUC_0-last_) using the linear trapezoidal rule. For Part I only, the same pharmacokinetic parameters were compared following a single oral dose of AL-335 400 mg administered as a tablet (in the fasted state) and an oral suspension (in the fed state). For Part II only, the same pharmacokinetic parameters were compared following a single oral dose of AL-335 400 mg administered under fasted (in Part I) or fed conditions (in Part II). Additionally in Part I, the urinary excretion of AL-335, ALS-022399, and ALS-022227 was determined following a single 800 mg dose of AL-335 under fasted conditions.

Plasma and urinary concentrations of AL-335, ALS-022399, and ALS-022227 were determined using validated liquid chromatography with tandem mass spectrometry (inVentiv Health Clinical Laboratories, Princeton, NJ, USA). The limits of quantification of plasma concentrations were 1.00 ng/mL for AL-335, 2.00 ng/mL for ALS-022399, and 5.00 ng/mL for ALS-022227. Limits of quantification of urine concentrations were 5 ng/mL for AL-335, 2 ng/mL for ALS-022399, and 20 ng/mL for ALS-022227.

**Pharmacodynamic assessments (Part III, MAD)**

HCV genotype was determined at screening using the VERSANT HCV Genotype 2.0 Assay (LiPA, Siemens Healthcare), which uses dual target analysis of 5’ non-coding and core viral genomic regions*.* Blood samples for HCV-RNA evaluation and viral resistance testing were taken at screening, Days -2, 1–12, 17, and 21. Plasma HCV-RNA was quantified using the COBAS^®^ Ampliprep/COBAS^®^ TaqMan^®^ HCV Test (Version 2.0, Roche) with a lower limit of quantification of 15 IU/mL. Monitoring of viral resistance to AL-335 was performed by population sequencing of the HCV nonstructural protein 5B polymerase coding region. Samples for viral resistance monitoring were selected at baseline, at end of dosing (Day 7), and at the end of the follow-up phase (Day 21), provided that HCV-RNA levels were above the limit for viral sequencing (1,000 IU/mL). The resistance analysis considered a list of 9 NS5B amino acid positions of interest that are associated with resistance to the class of nucleotide analogue NS5B inhibitors (NS5B amino acid positions 96, 142, 159, 223, 226, 282, 316, 320, and 321), including positions of specific interest for AL-335 based on *in vitro* findings [1, 2].

**Statistical analyses**

For the safety analyses (Parts I−III), out-of-range clinical laboratory parameters were flagged according to their Division of Acquired Immunodeficiency Syndrome toxicity grade [3].

For the pharmacokinetic analyses (Parts I−III), for plasma concentrations below the limit of quantification (BLQ), values occurring prior to the first detectable concentration were replaced by ‘0’, and if an entire concentration-time profile was BLQ, the profile was excluded from the pharmacokinetic analysis. All BLQ values occurring after the first detectable concentration were treated as missing.

Dose-proportionality of C_max_ and AUC_0–last_ values for AL-335, ALS-022399, and ALS-022227 was assessed using an exponential regression model. Pharmacokinetic parameters (C_max_ and AUC_0–last_) and dose values were log-transformed and evaluated using a
mixed-effects model with standard covariance structures including log_(dose)_ as fixed effect and subject as random effect. Dose-proportionality was declared if the two-sided 90% confidence interval for the slope value of log transformed dose was within the critical region [(1+ln(LL)/ln(r)), (1+ln(UL)/ln(r))] where r = Dose Maximum/Dose Minimum, LL (lower limit) = 0.8 and UL (upper limit) = 1.25.

**Reference**

1. Pawlotsky JM. Hepatitis C virus resistance to direct-acting antiviral drugs in interferon-free regimens. Gastroenterology 2016; 151:70-86.
2. Sarrazin C. The importance of resistance to direct antiviral drugs in HCV infection in clinical practice. J. Hepatol. 2016;64:486-504.
3. U.S. Department of Health and Human Services, National Institutes of Health, National Institute of Allergy and Infectious Diseases, Division of AIDS. Division of AIDS (DAIDS) Table for Grading the Severity of Adult and Pediatric Adverse Events, Corrected Version 2.1. 2017. Available from: <https://rsc.tech-res.com/docs/default-source/safety/division-of-aids-(daids)-table-for-grading-the-severity-of-adult-and-pediatric-adverse-events-corrected-v-2-1.pdf>

**Table A.** **Treatment-emergent adverse events (Part III; MAD).**

| **PART III**  **MAD** | **HCV GT1** | | | | **HCV GT1 +**  **compensated cirrhosis** | | **HCV GT2** | | **HCV GT3** | | **HCV GT4–6^a^** | |
| --- | --- | --- | --- | --- | --- | --- | --- | --- | --- | --- | --- | --- |
|  | **Placebo** | **AL-335**  **400 mg** | **AL-335**  **800 mg** | **AL-335**  **1,200 mg** | **Placebo** | **AL-335 800 mg** | **Placebo** | **AL-335 800 mg** | **Placebo** | **AL-335 800 mg** | **Placebo** | **AL-335 800 mg** |
| N | 6 | 8 | 8 | 8 | 2 | 8 | 2 | 8 | 2 | 8 | 1 | 3 |
| **All TEAEs** | **3 (50.0)** | **2 (25.0)** | **4 (50.0)** | **1 (12.5)** | **0 (0)** | **1 (12.5)** | **2 (100)** | **2 (25.0)** | **1 (50.0)** | **4 (50.0)** | **1 (100)** | **1 (33.3)** |
| Abdominal distension | 0 | 0 | 0 | 0 | 0 | 0 | 0 | 0 | 0 | 1 (12.5) | 0 | 0 |
| Abdominal pain upper | 0 | 0 | 0 | 0 | 0 | 0 | 0 | 0 | 0 | 1 (12.5) | 0 | 0 |
| Toothache | 0 | 0 | 0 | 0 | 0 | 0 | 0 | 0 | 1 (50.0) | 0 | 0 | 0 |
| Asthenia | 0 | 0 | 0 | 0 | 0 | 0 | 0 | 0 | 0 | 0 | 1 (100) | 0 |
| Fatigue | 0 | 1 (12.5) | 0 | 0 | 0 | 0 | 0 | 0 | 0 | 0 | 0 | 0 |
| Hyperbilirubinemia | 1 (16.7) | 0 | 0 | 0 | 0 | 0 | 0 | 0 | 0 | 1 (12.5) | 0 | 0 |
| Acute tonsillitis | 0 | 0 | 0 | 0 | 0 | 0 | 1 (50.0) | 0 | 0 | 0 | 0 | 0 |
| Nasopharyngitis | 0 | 0 | 1 (12.5) | 0 | 0 | 1 (12.5) | 0 | 0 | 0 | 0 | 0 | 0 |
| ALT increased | 1 (16.7) | 0 | 2 (25.0) | 0 | 0 | 0 | 0 | 0 | 0 | 0 | 0 | 0 |
| AST increased | 0 | 0 | 2 (25.0) | 0 | 0 | 0 | 0 | 0 | 0 | 0 | 0 | 0 |
| Blood bilirubin increased | 1 (16.7) | 0 | 0 | 0 | 0 | 0 | 0 | 0 | 0 | 0 | 0 | 0 |
| Blood cholesterol increased | 1 (16.7) | 0 | 0 | 0 | 0 | 0 | 0 | 0 | 0 | 0 | 0 | 0 |
| Blood creatinine phosphokinase increased | 1 (16.7) | 0 | 1 (12.5) | 0 | 0 | 0 | 0 | 0 | 0 | 0 | 0 | 0 |
| Lipase increased | 0 | 0 | 0 | 0 | 0 | 0 | 0 | 0 | 1 (50.0) | 1 (12.5) | 0 | 0 |
| Platelet count decreased | 0 | 0 | 0 | 0 | 0 | 0 | 0 | 0 | 0 | 1 (12.5) | 0 | 0 |
| Myalgia | 0 | 0 | 0 | 0 | 0 | 0 | 1 (50.0) | 0 | 0 | 0 | 0 | 0 |
| Headache | 2 (33.3) | 2 (25.0) | 1 (12.5) | 1 (12.5) | 0 | 0 | 0 | 2 (25.0) | 1 (50.0) | 1 (12.5) | 0 | 0 |
| Somnolence | 0 | 0 | 0 | 0 | 0 | 0 | 0 | 0 | 0 | 0 | 0 | 1 (33.3) |
| Pollakiuria | 1 (16.7) | 0 | 0 | 0 | 0 | 0 | 0 | 0 | 0 | 0 | 0 | 0 |

^a^All randomized subjects in the GT4–6 cohort were infected with GT4.

ALT = alanine aminotransferase, AST = aspartate aminotransferase, GT = genotype, HCV = hepatitis C virus, MAD = multiple ascending doses, TEAE = treatment-emergent adverse event.

**Table B. Plasma pharmacokinetic parameters of AL-335, ALS-022399, and ALS-022227 following single oral administration of AL-335 (tablet form) under fasted conditions (Part I).**

| **Parameter** | **AL-335 (tablet, fasted conditions)** | | | | |
| --- | --- | --- | --- | --- | --- |
|  | **100 mg** | **200 mg** | **400 mg** | **800 mg** | **1,200 mg** |
| **C_max_ (ng/mL), mean (SD)**  AL-335  ALS-022399  ALS-022227 | 13 (7.4)  7 (1.7)  215 (107.6) | 39 (26.8)  11 (2.6)  250 (52.4) | 72 (32.7)  27 (5.8)  482 (239.6) | 109 (74.7)  45 (15.1)  710 (338.3) | 91 (45.4)  55 (17.7)  699 (136.2) |
| **t_max_ (h), median (min, max)**  AL-335  ALS-022399  ALS-022227 | 0.5 (0.5, 2.0)  1.0 (0.5, 3.0)  2.5 (1.0, 3.0) | 0.5 (0.5, 2.0)  2.0 (0.5, 6.0)  3.0 (1.0, 4.0) | 0.5 (0.5, 1.0)  1.0 (0.5, 2.0)  2.5 (2.0, 4.0) | 0.75 (0.5, 1.0)  1.0 (1.0, 6.0)  2.5 (2.0, 3.0) | 0.75 (0.5, 1.0)  2.5 (1.0, 8.0)  4.0 (2.0, 4.0) |
| **AUC_0–last_ (ng.h/mL), mean (SD)**  AL-335  ALS-022399  ALS-022227 | 11 (4.1)  18 (7.9)  1,014 (537.4) | 48 (27.5)  75 (51.7)  1,377 (286.3) | 93 (59.5)  120 (29.4)  2,779 (1333) | 155 (121.0)  263 (54.6)  4,270 (1707) | 144 (61.4)  371 (128.0)  4,757 (702.2) |
| **t_1/2_ (h), mean (SD)**  AL-335  ALS-022399  ALS-022227 | ND  ND  2.0 (0.2) | 1. (0.7)   ND  2.1 (0.2) | 0.6 (0.2)  2.4 (0.3)  3.1 (1.5) | 0.6 (0.1)  2.9 (0.4)  4.4 (1.4) | 0.8 (0.5)  3.0 (0.7)  5.6 (1.9) |

AUC_0–last_ = area under the concentration-time curve from time zero to last sample with measurable plasma concentration, C_max_ = maximum measured drug concentration, ND = not determined as more than half of the values were missing or not reliably determined, SD = standard deviation, t_½_ = apparent terminal elimination half-life, t_max_ = time of maximum concentration.

**Table C. Plasma pharmacokinetic parameters of AL-335, ALS-022399, and ALS-022227 on Days 1 and 7 following multiple oral administrations of AL-335 (tablet form) under fed conditions (Part III, Days 1–7).**

| **Parameter** | **AL-335 (tablet, fed conditions)** | | | | | | | | | | | | | |
| --- | --- | --- | --- | --- | --- | --- | --- | --- | --- | --- | --- | --- | --- | --- |
|  | **400 mg (GT1)** | | **800 mg (GT1)** | | **1,200 mg (GT1)** | | **800 mg (GT1  with cirrhosis)** | | **800 mg (GT2)** | | **800 mg (GT3)** | | **800 mg (GT4–6)** | |
|  | **Day 1** | **Day 7** | **Day 1** | **Day 7** | **Day 1** | **Day 7** | **Day 1** | **Day 7** | **Day 1** | **Day 7** | **Day 1** | **Day 7** | **Day 1** | **Day 7** |
| **C_max_ (ng/mL), mean (SD)** | | | | | | | | | | | | | | |
| AL-335  ALS-022399  ALS-022227 | 100 (53)  38 (19)  536 (140) | 77 (43)  31 (14)  482 (181) | 188 (74)  65 (27)  508 (281) | 140 (48)  52 (16)  478 (148) | 325 (192)  111 (38)  628  (281) | 301 (192)  124 (50)  750  (299) | 404 (241)  111 (52)  733 (286) | 214 (111)  81 (39)  649 (327) | 146 (52)  55 (16)  450 (145) | 124 (62)  57 (16)  504  (169) | 164 (76)  63 (17)  597  (196) | 134 (50)  77 (26)  613  (109) | 203 (106)  60 (22)  381 (162) | 187 (155)  58 (11)  369  (46) |
| **t_max_ (h), median (min, max)** | | | | | | | | | | | | | | |
| AL-335  ALS-022399  ALS-022227 | 1.5 (0.5, 3.0)  2.0 (2.0, 4.0)  3.0 (3.0, 4.0) | 2.0 (0.5, 2.0)  3.0 (1.0, 3.0)  3.0 (2.0, 4.0) | 1.0 (0.5, 4.0)  2.5 (2.0, 6.0)  4.0 (3.0, 6.0) | 3.0 (1.0, 4.0)  4.0 (3.0, 4.0)  4.0 (3.0, 6.0) | 1.0 (1.0, 2.0)  3.0 (2.0, 6.0)  4.0 (3.0, 4.0) | 2.0 (1.0, 3.0)  3.0 (2.0, 6.0)  4.0 (4.0, 4.0) | 1.0 (0.5, 2.0)  2.0 (1.0, 3.0)  3.0 (2.0, 4.0) | 2.0 (1.0, 3.0)  3.0 (2.0, 4.0)  4.0 (2.0, 6.0) | 2.0 (1.0, 6.0)  2.0 (2.0, 6.0)  4.0 (2.0, 6.0) | 2.5 (1.0, 4.0)  3.5 (2.0, 6.0)  4.0 (3.0, 6.0) | 2.5 (1.0, 3.0)  3.5 (2.0, 12.0)  4.0 (3.0, 4.0) | 2.5 (1.0, 3.0)  3.5 (2.0, 8.0)  4.0 (4.0, 6.0) | 1.0 (1.0, 4.0)  2.0 (2.0, 4.0)  4.0 (4.0, 6.0) | 2.0 (1.0, 3.1)  3.0 (2.0, 3.1)  4.0 (4.0, 6.1) |
| **AUC_0–last_ (ng.h/mL), mean (SD)** | | | | | | | | | | | | | | |
| AL-335  ALS-022399  ALS-022227 | 189 (129)  169 (90)  3,264 (963) | 173 (143)  131 (60)  2,991 (906) | 392 (204)  333 (136)  3,373 (1,380) | 331 (122)  318 (75)  4,093 (577) | 655 (311)  661 (300)  4,231 (1,377) | 700 (366)  814 (319)  6,227 (1,738) | 742 (417)  454 (203)  4,273 (1,226) | 491 (186)  422 (275)  4,965 (1,312) | 334 (171)  309 (108)  3,185 (1,155) | 323 (163)  308 (105)  4,207 (1,404) | 304 (108)  335 (79)  3,951 (1,279) | 323 (124)  503 (413)  5,006 (996) | 498 (344)  330 (80)  2,653 (1,382) | 536 (570)  324 (79)  3,510 (1,141) |
| **t_1/2_ (h), mean (SD)** | | | | | | | | | | | | | | |
| AL-335  ALS-022399  ALS-022227 | ND  2.4 (0.4)  3.3 (0.5) | ND  2.2 (0.4)  8.2 (4.3) | 0.7 (0.1)  2.6 (0.8)  4.3 (0.6) | 0.7 (0.1)  3.4 (1.3)  15.6 (4.3) | 0.7 (0.1)  3.2 (2.0)  5.2 (1.2) | 0.8 (0.2)  3.2 (1.2)  27.9 (3.6) | 0.6 (0.1)  2.1 (0.5)  4.6 (1.1) | 0.6 (0.1)  2.4 (0.8)  16.9 (5.5) | 0.7 (0.1)  2.8 (0.5)  4.3 (0.7) | 0.7 (0.1)  2.7 (1.0)  15.5 (2.9) | 0.6 (0.1)  2.9 (0.8)  3.7 (0.3) | 0.6 (0.1)  2.7 (0.8)  16.6 (2.5) | 0.7 (0.1)  2.8 (0.6)  4.7 (0.7) | ND  2.9 (1.0)  18.7 (7.4) |

AUC_0–last_ = area under the concentration-time curve from time zero to last sample with measurable plasma concentration, C_max_ = maximum measured drug concentration, GT = genotype, ND = not determined as more than half of the values were missing or not reliably determined, SD = standard deviation,
t_½_ = apparent terminal elimination half-life, t_max_ = time of maximum concentration.
