# Supplementary material for: Safety, tolerability, and pharmacokinetics of AL-335 in healthy volunteers and hepatitis C virus-infected subjects
Source: PLoS One. 2018 Oct 16;13(10):e0204974. doi: 10.1371/journal.pone.0204974 (PMC6191080; doi:10.1371/journal.pone.0204974)
Supplement: S2 Appendix — (PDF) [file pone.0204974.s002.pdf]

Alios BioPharma, Inc
260 E. Grand Ave
South San Francisco, CA 94080

Protocol Number: AL-335-601

A Randomized, Double-blind, Placebo-controlled, First-in-human, 3-Part Study of Orally Administered AL-335 to Evaluate the Safety, Tolerability, and Pharmacokinetics of Single Ascending Dosing and Food-effect in Healthy Volunteers, and Multiple Ascending Dosing in Subjects with Chronic Hepatitis C Genotype 1 Infection

Drug Name: AL-335

IND Number: This is a non-IND study

EudraCT Number: 2014-003750-14

Date: April 15, 2015

Version 3.0

CONTACT INFORMATION

For up-to-date contact information, see Study Binder: Emergency Contact Sheet.

All serious pretreatment and treatment emergent adverse events, including pregnancies and suspected pregnancies,* occurring **from the time of** consent up to 90 days after study drug administration is completed, regardless of relationship to study drug, must be reported via fax to ICON Pharmacovigilance within 24 hours of knowledge of the event.

Please fax all Serious Adverse Event Forms to:

Icon Pharmacovigilance	
**SAE Email Address**	[redacted]
**UK SAE Fax Number**:	[redacted]

Any fatal or life-threatening event, pregnancy or suspected pregnancy must also be reported within 24 hours by telephone

* See Sections 7.1.1 and 7.1.3 for the definitions of *pretreatment adverse event* and *serious adverse event. Pregnancy itself does not constitute an adverse event.*

TABLE OF CONTENTS

CONTACT INFORMATION 2

TABLE OF CONTENTS 3

IN-TEXT FIGURES 6

IN–TEXT TABLES 6

ABBREVIATIONS 7

LIST OF STUDY DRUGS AND METABOLITES 9

SYNOPSIS 10

1.0 BACKGROUND AND RATIONALE 20

1.1 Background Information 20
1.2 Rationale for the Study 22
1.3 Rationale for Dose Selection 23

2.0 STUDY DESIGN 24

2.1 Summary 24
2.2 Study Schema 26

3.0 STUDY OBJECTIVES AND ENDPOINTS 27

3.1 Study Objectives 27
3.1.1 Primary Objective 27
3.1.2 Secondary Objectives 27
3.2 Study Endpoints 28
3.2.1 Primary Endpoints 28
3.2.2 Secondary Endpoints 28

4.0 SELECTION AND WITHDRAWAL OF SUBJECTS 28

4.1 Study Population 28
4.2 Inclusion Criteria 29
4.3 Exclusion Criteria 30
4.4 Subject Screening and Enrollment 31
4.5 Subject Discontinuation 32
4.5.1 Subject Discontinuation Criteria 32
4.5.2 Procedures for Subjects Who Discontinued 33
4.5.3 Documentation of Withdrawal of Subjects 33
4.6 Study Discontinuation 33

5.0 TREATMENT OF SUBJECTS 33

5.1 Treatment Regimens 33
5.1.1 Cohort Progression Guidelines 34

5.2 Description of Study Drug and Background Therapy 36
5.2.1 Study Drug 36
5.3 Dose Preparation and Administration 36
5.4 Ordering Study Drug 37
5.5 Drug Accountability 37
5.6 Disposition of Used, Partially Used, and Unused Study Medication Containers 37
5.7 Concomitant Medications 37
5.8 Prohibited Medications 38
6.0 STUDY PROCEDURES 38
6.1 Schedule of Events 38
6.2 On-study Evaluations, Procedures, and Dosing 47
6.2.1 Diet, Fluid, and Activity 47
6.2.2 Pharmacokinetic Blood and Urine Sampling 48
6.2.3 Central Laboratory Evaluations 48
6.2.4 HCV Evaluations 48
6.2.5 HCV Resistance Monitoring 49
6.2.6 12-Lead Electrocardiograms 49
6.2.7 Vital Signs 49
6.2.8 Physical Examinations 49
6.2.9 Contraception 49
7.0 SAFETY MONITORING AND REPORTING 50
7.1 Definitions 50
7.1.1 Pretreatment Events 50
7.1.2 Adverse Events 50
7.1.3 Serious Adverse Events 50
7.2 Documenting and Reporting of Adverse Events (including Serious Adverse Events) 51
7.2.1 Documenting and Reporting Pretreatment Events 51
7.2.2 Documenting and Reporting Adverse Events 51
7.2.3 Assigning Attribution of Adverse Events 53
7.2.4 Classifying Action Taken with Study Drug 53
7.2.5 Classifying Adverse Event Outcome 54
7.2.6 Documenting and Reporting Serious Pretreatment Events and Serious Adverse Events 54
7.2.7 Documenting and Reporting of Pregnancy 55
7.3 Follow-up of Adverse Events and Serious Adverse Events 55
7.4 Sponsor's Review of Adverse Events and Serious Adverse Events 55
7.5 Emergency Unblinding For Parts 1 and 2 (SAD and Food Effect) 55

7.6 Emergency Unblinding for Part 3 (MAD) 56

8.0 STUDY VARIABLES AND MEASUREMENTS 56

8.1 Efficacy Variables/Measurements 56

8.2 Safety Variables/Measurements 56

8.2.1 Adverse Events 56

8.2.2 Clinical Laboratory Measurements 56

8.2.3 Prior and Concomitant Medications 56

8.3 Pharmacokinetic Measurements 57

8.4 Pharmacodynamic Measurements 57

8.5 Viral Resistance 57

9.0 STATISTICAL CONSIDERATIONS 57

9.1 General Considerations 57

9.2 Study Endpoints 58

9.2.1 Primary Endpoint 58

9.2.2 Secondary Endpoints 58

9.3 Determination of Sample Size 58

10.0 RANDOMIZATION 59

10.1 Study Conduct 59

10.1.1 Subject Disposition 59

10.1.2 Replacement of Subjects 59

10.1.3 Procedures for Handling Missing, Unused, or Spurious Data 59

10.2 Analysis Data Sets 59

10.3 Demographics and Baseline Characteristics 59

10.4 Safety Analysis 60

10.4.1 Adverse Events 60

10.4.2 Vital Signs, ECG, Physical Examination, and Laboratory Assessments 60

10.4.3 Concomitant Medications 60

10.5 Pharmacokinetic Analysis 60

10.6 HCV Viral Load Analysis 60

11.0 ADMINISTRATIVE CONSIDERATIONS 61

11.1 Study Compliance 61

11.2 Informed Consent and Protected Subject Health Information Authorization 61

11.3 Subject Screening Log 62

11.4 Case Report Forms 62

11.5 Study Monitoring Requirements 62

11.6 Retention of Records 62

11.7 Confidentiality and Publication Policy 63

11.8 Conduct of Study and Protection of Human Subjects 63

12.0 REFERENCES 64

13.0 APPENDICES 66

IN-TEXT FIGURES

Figure 2.2-1. Dosing Schema 27

IN–TEXT TABLES

Table 1.3-1. Parameters Used in Determining Human Projected Efficacious Doses 23

Table 1.3-2. Safety Margin and Starting Doses per FDA Guidelines 24

Table 5.1-1. Dosing Regimen 33

Table 6.1-1. Schedule of Events SAD Phase (Part 1) 39

Table 6.1-2. Schedule of Events for Food Effect Phase (Part 2) 41

Table 6.1-3. Schedule of Events for MAD Phase (Part 3) 43

Table 6.1-4. Schedule of Events for Resistance Monitoring 46

ABBREVIATIONS

Term	Definition
AE	Adverse event
Ae	Total amount excreted in urine
ALT	Alanine aminotransferase
AST	Aspartate aminotransferase
$AUC_{0\text{-}inf}$	Area under the concentration-time curve from time zero to infinity
$AUC_{0\text{-}last}$	Area under the concentration-time curve from time zero to last sample with measurable plasma concentration
$AUC_{0\text{-}tau}$	Area under plasma concentration-time curve during the dosing interval
BMI	Body Mass Index
BP	Blood pressure
C_{last}	Last measurable plasma concentration
C_{max}	Maximum measured drug concentration
C_{min}	Minimum measured concentration drug concentration
CBC	Complete blood count
CDER	Center for Drug Evaluation and Research
CHC	Chronic Hepatitis C
CL	Systemic clearance
$Cl_{R(0\text{-}t)}$	Renal clearance over 0 to t time period
CL/F	Apparent oral clearance
CRF	Case report form
CSR	Clinical Study Report
DAA	Direct-acting antiviral agent
EC	Ethics Committee
EC_{50}	Half maximal effective concentration
EC_{90}	90% maximal effective concentration
ECG	Electrocardiogram
EU	European Union
FA	Full Analysis set
FDA	Food and Drug Administration
FE	Food Effect
FSH	Follicle Stimulating Hormone
GCP	Good Clinical Practice
HAV IgM	Hepatitis A immunoglobulin
HBsAg	Hepatitis B surface antigen
HCC	Hepatocellular carcinoma
HCV	Hepatitis C virus
HED	Human equivalent dose
HIV	Human Immunodeficiency Virus
HR	Heart rate
HV	Healthy volunteer
ICF	Informed Consent Form
ICH	International Conference on Harmonisation

Term	Definition
IEC	Independent Ethics Committee
IFN	Interferon
INR	International Normalization Ratio
IRB	Institutional Review Board
K_i	Dissociation constant for inhibitor binding
kPa	Kilo Pascal
λz	Terminal elimination rate constant
MAD	Multiple ascending dose
MedDRA	Medical Dictionary for Regulatory Activities
NOAEL	No-observed-adverse-effect level
NSAID	Non-steroidal anti-inflammatory drug
NTP	Nucleoside triphosphate
PD	Pharmacodynamics
PE	Physical examination
PEG-IFN	Pegylated interferon
PI	Principal investigator
PK	Pharmacokinetic(s)
PK/PD	Pharmacokinetic/Pharmacodynamic
PO	Orally, by mouth
PRN	*Pro re nata*
PT	Prothrombin Time
PTT	Partial Thromboplastin Time
qd	Once daily
RBC	Red blood cell
RBV	Ribavirin
RNA	Ribonucleic acid
RR	Respiratory Rate
RVR	Rapid virological response
SAD	Single ascending dose
SAE	Serious adverse event
SAP	Statistical analysis plan
SOC	System Organ Class
SUSAR	Serious and Unexpected Adverse Reaction
SVR	Sustained viral response
$t_{1/2}$	Half-life
t_{last}	Time to last measurable plasma concentration
t_{max}	Time of maximum concentration
ULN	Upper limit of normal
V_z/F	The apparent steady state volume of distribution
WHO	World Health Organization

LIST OF STUDY DRUGS AND METABOLITES

Compound Number	Alternative Compound Number	Comment
AL-335	ALS-022335	Investigational drug; *S*p isomer; phosphoramidate prodrug of the uridine nucleoside analog ALS-022227
ALS-022227	-	parent uridine nucleoside analog, major metabolite of AL-335
ALS-022316	-	5'-monophosphate of AL-022227 (NMP)
ALS-022235	-	5'-triphosphate of ALS-022227 (NTP), active entity
ALS-022399	-	5'-monophosphate precursor, major metabolite of AL-335

NTP: nucleoside triphosphate, NMP: nucleoside monophosphate

SYNOPSIS

A Randomized, Double-blind, Placebo-controlled, First-in-human, 3-Part Study of Orally Administered AL-335 to Evaluate the Safety, Tolerability, and Pharmacokinetics of Single Ascending Dosing and Food-effect in Healthy Volunteers, and Multiple Ascending Dosing in Subjects with Chronic Hepatitis C Genotype 1 Infection

**Protocol Number:**	AL-335-601
**Phase:**	1
**Study Drug:**	AL-335
**IND Number:**<br>**EudraCT Number:**	This is a non-IND study<br>2014-003750-14
**Background Therapy:**	None
**Comparator:**	None
**Indication:**	Chronic hepatitis C (CHC)
**Study Design:**	This randomized, double-blind, placebo-controlled, 3-part study will assess the safety, tolerability, and pharmacokinetics of orally administered AL-335 in healthy volunteers (HV) and subjects with CHC infection.<br><br>Part 1: HV will receive one of 5 single ascending doses (SAD) of AL-335 ranging from 100 mg to 1200 mg. Within each cohort subjects will be randomized to receive either AL-335 or placebo (n=8 per cohort; 6 assigned to AL-335 and 2 assigned to placebo) in a fasted state. Up to three additional cohorts may be evaluated at the discretion of the Sponsor and Principal Investigator (PI) based on the emerging pharmacokinetic (PK) profile and the presence of an acceptable safety profile as outlined in Section 5.1.1. The additional cohorts may be administered an oral suspension or tablet formulation of AL-335 corresponding to one or more of the planned dose levels or to evaluate a higher dose. These doses will be evaluated under fed conditions.<br><br>Part 2: Eight HV from Cohort 3 in Part 1 will receive a second single dose of AL-335 or placebo (as per their randomized assignment in Part 1) after a washout period of 11 to 21 days to assess food effects on pharmacokinetics.<br><br>Part 3: The following cohorts of 10 subjects with CHC infection will be evaluated.<br><br>Subjects with CHC genotype 1 infection will receive one of

3 ascending doses of AL-335 (400 mg, 600 mg, or 800 mg) or placebo dosed once daily for 7 days (n=10 per cohort, 8 assigned to AL-335 and 2 assigned to placebo). Based on the emerging data, an additional cohort of 10 subjects with CHC genotype 1 infection may be enrolled to evaluate an alternative dose of AL-335.

The following cohorts may be enrolled after a review of the safety and efficacy of the initial MAD cohorts which will be conducted by the sponsor in consultation with the principal investigators. The dose to be administered will have demonstrated at least a mean 3 $\log_{10}$ reduction in HCV RNA concentrations in a prior cohort of genotype 1 subjects in combination with an acceptable safety and PK profile.

- Genotype 2: a cohort of ten (10) treatment naïve subjects with genotype 2 CHC infection will be enrolled (8 assigned to AL-335 and 2 assigned to placebo) to evaluate AL-335 dosed once daily for 7 days.
- Genotype 3: a cohort of ten (10) treatment naïve subjects with genotype 3 CHC infection will be enrolled (8 assigned to AL-335 and 2 assigned to placebo) to evaluate AL-335 dosed once daily for 7 days.
- Genotypes 4-6: a cohort of ten (10) treatment naïve subjects with genotypes 4-6 CHC infection will be enrolled (8 assigned to AL-335 and 2 assigned to placebo) to evaluate AL-335 dosed once daily for 7 days.
- Compensated cirrhosis: a cohort of ten (10) subjects with CHC genotype 1 infection and documented compensated cirrhosis (Child Pugh Class A) will be enrolled (8 assigned to AL-335 and 2 assigned to placebo) to evaluate AL-335 dosed once daily for 7 days Sequence analysis of HCV NS5b from CHC GT1 cohorts assigned to receive either AL-335 or placebo will be completed prior to enrollment of subjects with compensated cirrhosis and CHC GT1.

In Parts 1–2, the doses to be evaluated may be modified based on emerging PK and safety data. However, there will be no more than a three-fold increase in dose per cohort (see *Cohort Progression Guidelines*, Section 5.1.1) and the mean exposure for ALS-022227 ($AUC_{0\text{-}24}$) at a dose level will be projected not to exceed 75,500 ng·h/mL.

In Part 3, the doses to be evaluated may be modified based on emerging PK, HCV RNA and safety data. However, the maximum dose

	administered will not exceed the maximum tolerated dose observed in Part 1; the mean exposure for ALS-022227 (AUC_{0-24}) at a dose level will be projected not to exceed 75,500 ng·h/mL; and there will be no more than a three-fold increase in dose per cohort (see *Cohort Progression Guidelines*, Section 5.1.1).<br><br>In Parts 1–3, an increase in dose will only occur if the previous dose is demonstrated to be generally safe and tolerable (see *Cohort Progression Guidelines*, Section 5.1.1). Before the second and subsequent cohorts are conducted, safety data from the prior cohort will be reviewed by the Principal Investigator (PI) and Sponsor.<br><br>Part 3 will commence when the third dose of Part 1 is found to be safe and tolerable based upon review of the blinded safety data by the PI and the Sponsor. Dosing will occur after a standard meal.<br><br>Safety and tolerability will be evaluated on an ongoing basis through assessment of adverse events, blood and urine sample analyses, collection of electrocardiograms (ECG), vital signs and physical examinations.<br><br>The Sponsor will provide access to components of standard of care for subjects with CHC participating in Part 3 who are unable to access it locally, through an appropriate mechanism for each participating country, subject to local regulations. To be eligible, subjects with CHC must complete the study and commence treatment within 6 months of the study completion visit.<br><br>See Section 6.0 for Study Procedures.
**Study Objectives:**	Primary:<br>• To evaluate the safety and tolerability of single and multiple doses of AL-335 administered to HV and subjects with CHC infection, respectively<br><br>Secondary:<br>• To evaluate, after single and multiple doses of AL-335, the pharmacokinetics of AL-335, ALS-022399 and ALS-022227 (and other metabolites if applicable) in plasma and urine (single doses only)<br>• To evaluate, after a single dose of AL-335, the effect of dosage formulation (tablet versus suspension) on the pharmacokinetics of AL-335, ALS-022399 and ALS-022227 (and other metabolites if applicable) in plasma<br>• To evaluate the effect of food intake on the pharmacokinetics

	of AL-335, ALS-022399 and ALS-022227 (and other metabolites if applicable)<br>• To evaluate the viral kinetics of HCV RNA in subjects with CHC infection treated with AL-335<br>• To evaluate the viral resistance profile after 7 daily doses of AL-335 in subjects with CHC infection<br>• To characterize the relationship between plasma exposures of AL-335 and/or its metabolites and viral kinetics
**Primary Endpoint:**	• Safety data including but not limited to treatment emergent adverse events, physical examination findings, vital signs, 12-lead ECG and clinical laboratory results (including chemistry, hematology, and urine)
**Secondary Endpoints:**	• PK parameters of AL-335, ALS-022399, ALS-022227 (and other metabolites if applicable) following single dose administration: C_{max}, t_{max}, $t_{1/2}$, CL/F and V_z/F (for AL-335 only), $AUC_{0\text{-}inf}$ or AUC_{last}<br>• PK parameters of AL-335, ALS-022399, ALS-022227 (and other metabolites if applicable) following repeat dose administration: C_{max}, t_{max}, $t_{1/2}$, AUC_{last} and $AUC_{0\text{-}tau}$<br>• PK parameters of AL-335, ALS-022399, ALS-022227 (and other metabolites if applicable) following single dose administration in tablet form as compared with suspension: C_{max}, t_{max}, $t_{1/2}$, CL/F and V_z/F (for AL-335 only), $AUC_{0\text{-}inf}$ or AUC_{last}<br>• PK parameters of AL-335, ALS-022399, ALS-022227 (and other metabolites if applicable) after a single oral dose in HV in fasted conditions as compared with fed conditions<br>• Concentrations in urine and urinary excretion of AL-335, ALS-022399, ALS-022227 (and other metabolites if applicable) after a single oral dose in HV in fasted conditions<br>• HCV ribonucleic acid (RNA) viral load change from baseline in subjects with CHC infection<br>• Sequence analysis of the HCV NS5B region in subjects with CHC infection, as appropriate
**Duration of Treatment and Study Period:**	Part 1: Single dose in a fasted state (initial 5 cohorts) or fed state (optional cohorts)<br>Part 2: Single dose in a fasted state (Cohort 3 from Part 1) followed by

	a single dose in a fed state after an 11- 21 day washout<br>Part 3: Once daily dosing for 7 consecutive days after a standard meal.
**Number of Sites and Location:**	Up to 6 sites in France, Moldova, Georgia and Romania
**Sample Size:**	Part 1 (SAD):<br>Approximately 40 HV (n=8 per cohort) who will receive one dose of AL-335 or placebo. An additional 24 HV may be enrolled into three additional dose cohorts at the discretion of the PI and Sponsor.<br>Part 2 (Food Effect):<br>Approximately 8 HV who will receive one dose of AL-335 or placebo on two occasions, one initially in a fasted state (Cohort 3 from Part 1) and one in a fed state, separated by an 11 to 21day washout (Part 2).<br>Part 3 (MAD):<br>Approximately 70 (up to 80) subjects with CHC infection who will be dosed once daily with AL-335 or placebo for 7 days. Dosing will take place after a standard meal.
**Inclusion Criteria:**	Main Inclusion Criteria for All Subjects:<br>• Subject has provided written consent.<br>• In the investigator's opinion, the subject is able to understand and comply with protocol requirements, instructions, and protocol-stated restrictions and is likely to complete the study as planned.<br>• Subject is in good health as deemed by the investigator, based on the findings of a medical evaluation including medical history, physical examination, laboratory tests, and ECG.<br>• Male or female, 18–60 years of age for HV and 18–65 years of age for subjects with CHC.<br>• Body mass index (BMI) 18–32 kg/m^2, inclusive, for HV and 18–35 kg/m^2, inclusive, for subjects with CHC. The minimum weight is 50 kg in both populations. No more than 25% of patients in any cohort may be enrolled with a BMI ≥ 30 kg/m^2.<br>• A female subject is eligible to participate in this study if she is of non-childbearing potential (defined as females with a documented tubal ligation, bilateral oophorectomy, or hysterectomy) or postmenopausal (defined as 12 months of spontaneous amenorrhea and follicle stimulating hormone (FSH) level within the laboratory's reference range for

	postmenopausal females). A postmenopausal female receiving hormone replacement therapy who is willing to discontinue hormone therapy 28 days before study drug dosing and agrees to remain off hormone replacement therapy for the duration of the study may be eligible for study participation.<br>• If male, subject is surgically sterile or practicing specific forms of birth control (as outline in Section 6.2.9) until 90 days after the end of the study.<br>Additional inclusion criteria for subjects with CHC infection:<br>• Documentation of HCV infection for greater than 6 months at dosing<br>• Screening HCV RNA viral load ≥ 10^5 IU/mL using a sensitive quantitative assay, such as COBAS® Taqman® HCV Test 2.0, except for subjects with compensated cirrhosis (Child-Pugh Class A) who may have HCV RNA viral load ≥10^4 and ≤10^8 IU/mL<br>• In addition, subjects with compensated cirrhosis and CHC genotype 1 infection and must meet the Child-Pugh Class A definition (see Appendix F) and at least one of the following criteria:<br>a. Liver biopsy result within 6 months of Day 1 indicating the presence of cirrhosis (e.g., Metavir F4; Ishak > 5) or<br>b. Fibroscan evaluation within 3 months of Day 1 with a liver stiffness score ≥ 14.5 kPa
**Exclusion Criteria:**	Subjects will be ineligible for this study if they meet **any one** of the following criteria:<br>1. Clinically significant cardiovascular, respiratory, renal, gastrointestinal, hematologic, neurologic, thyroid, or any other medical illness or psychiatric disorder, as determined by the Investigator and/or Sponsor's Medical Monitor.<br>2. Positive test for HAV IgM, HBsAg, or HIV antibody. In Parts 1 and 2 positive HCV serology is exclusionary.<br>3. Any condition that, in the opinion of the investigator, would compromise the study's objectives or the well-being of the subject or prevent the subject from meeting the study requirements.<br>4. Participation in an investigational drug trial or having received an investigational vaccine within 30 days or 5 half-lives

(whichever is longer) prior to study medication.

5. Clinically significant abnormal ECG findings. Particularly, a history or family history of prolonged QT syndrome (e.g., torsade de pointes) or sudden cardiac death; or a corrected QT interval (QTc) > 450 milliseconds for male subjects and > 470 milliseconds for female subjects at the Screening Visit.
6. Clinically significant blood loss or elective blood donation of significant volume (i.e., > 500 mL) within 60 days of first dose of study drug; > 1 unit of plasma within 7 days of first dose of study drug.
7. Abnormal heart rate, respiratory rate, temperature or blood pressure values outside of the normal range (evaluated in a semi-recumbent or recumbent position after 5 minutes of rest). One repeat measurement after an additional 5 minutes of rest is permitted.
8. Evidence of active infection (other than CHC infection in subjects enrolled in Part 3).
9. Unwilling to abstain from alcohol for 48 hours prior to the start of dosing through the study completion visit.
10. History of regular alcohol intake > 7 units per week of alcohol for females and > 14 units per week for males (one unit is defined as 10 g alcohol) within 3 months of screening visit.
11. For healthy subjects (Parts 1–2), history of regular use of tobacco (i.e., ≥10 cigarettes per day) or nicotine-containing products within 3 months of the screening visit. For subjects with CHC infection, history of regular use of tobacco- or nicotine-containing products is allowed.
12. The subject has a positive pre-study drug screen. A minimum list of drugs that will be screened for includes amphetamines, barbiturates, cocaine, opiates, cannabinoids, and benzodiazepines. Subjects with CHC may be included if they have a positive result for cannabinoids at screening. However, they must be willing to abstain from cannabinoid use throughout the duration of the study.
13. In Parts 1–2, the use of concomitant medications, including prescription, over the counter medications, or herbal medications within 14 days prior to the first dose of study medication is excluded, unless approved by the Sponsor's Medical Monitor. PRN use of a nonsteroidal anti-inflammatory

drug (NSAID) is permitted.

14. For subjects in Part 3, the use of prescription and over the counter medications deemed necessary to maintain the health status of the subject are permitted, if approved by the Sponsor's Medical Monitor. PRN NSAID use is permitted. Further guidance for the use of prior medication in subjects with CHC can be found in Section 5.7.
15. Subjects must not have received any drug known to be a strong inducer or inhibitor of CYP450 enzymes within 2 weeks prior to study drug dosing (Appendix C).
16. Exposure to more than four new investigational entities within 12 months prior to the first dosing day.
17. Abnormal biochemistry or hematology laboratory results obtained at screening. Elevated bilirubin in subjects with suspected Gilbert's disease is allowed.

A subject with a clinical abnormality or laboratory parameters outside the reference range for the population being studied may be included if the Investigator and the Sponsor's Medical Monitor agree that the finding is unlikely to introduce additional risk factors and will not interfere with data interpretation. A single repeat laboratory evaluation is allowed for eligibility determination. The Investigator is encouraged to discuss any laboratory abnormalities which are considered potentially clinically significant with the Sponsor Medical Monitor prior to randomization.

Additional exclusion criteria for subjects with CHC infection:

18. History of clinical hepatic decompensation, e.g., variceal bleeding, spontaneous bacterial peritonitis, ascites, hepatic encephalopathy or active jaundice (within the last year)
19. For all MAD Cohorts (except subjects with compensated cirrhosis), a liver biopsy within two years or Fibroscan evaluation within 6 months prior to randomization that demonstrates cirrhosis (Knodell score > 3, Metavir score > 3, Ishak score > 4). Fibroscan liver stiffness score > 10.5 kPa.
20. Prior treatment for CHC with direct acting antiviral agents
21. Serum alanine aminotransferase (ALT) concentration >5 x ULN
22. Evidence on screening liver ultrasound of hepatic mass or lesion concerning for malignancy.

**Dosage Form and Strength:**	AL-335 will be supplied as tablets in two strengths, 25 mg and 100 mg, and as a powder for reconstitution (10 g per bottle). Matching placebos will be provided for all dosage forms of AL-335.
**Dose Regimen:**	**Part 1.** The planned doses of AL-335 to be evaluated:<br><br>Cohort 1: 6 subjects to receive single oral 100 mg dose of AL-335 in a fasted state with 2 subjects receiving matching placebo<br><br>Cohort 2: 6 subjects to receive single oral 200 mg dose of AL-335 in a fasted state with 2 subjects receiving matching placebo<br><br>Cohort 3: 6 subjects to receive single oral 400 mg dose of AL-335 in a fasted state with 2 subjects receiving matching placebo<br><br>Cohort 4: 6 subjects to receive single oral 800 mg dose of AL-335 in a fasted state with 2 subjects receiving matching placebo<br><br>Cohort 5: 6 subjects to receive single oral 1,200 mg dose of AL-335 in a fasted state with 2 subjects receiving matching placebo<br><br>Cohort 6: 6 subjects to receive single 400 mg oral dose of AL-335 (oral suspension formulation) in a fed state, with 2 subjects receiving matching placebo<br><br>Cohort 7 (optional): 6 subjects to receive single oral dose of AL-335 in a fed or fasted state at a dose to be determined and administered as either a tablet or oral suspension, with 2 subjects receiving matching placebo<br><br>Cohort 8 (optional): 6 subjects to receive single oral dose of AL- in a fed or fasted state at a dose to be determined and administered as either a tablet or oral suspension, with 2 subjects receiving matching placebo<br><br>**Part 2.** The effect of food will be evaluated in 8 subjects who received the 3rd dose fasted in Part 1 (Cohort 3). These subjects will also receive AL-335 or placebo in fed (high fat meal) conditions after an 11 to 21 day washout period.<br><br>**Part 3.** The multiple-dose escalation phase will evaluate:<br><br>Cohort 1: 8 CHC subjects (genotype 1) will be assigned to receive 400 mg of AL-335 given once daily on 7 consecutive days in a fed state with 2 subjects receiving matching placebo.<br><br>Cohort 2: 8 CHC subjects (genotype 1) will be assigned to receive 600 mg of AL-335 given once daily on 7 consecutive days in a fed state with 2 subjects receiving matching placebo.

	Cohort 3: 8 CHC subjects (genotype 1) will be assigned to receive 800 mg AL-335 given once daily on 7 consecutive days in a fed state with 2 subjects receiving matching placebo.<br><br>Cohort 4 (optional): 8 CHC subjects (genotype 1) will be assigned to receive AL-335 given once daily on 7 consecutive days in a fed state with 2 subjects receiving matching placebo. The dose to be administered will be determined by the Sponsor after completion of Cohort 3.<br><br>Cohort 5: Eight (8) CHC subjects (genotype 2) will be assigned to receive AL-335 given once daily on 7 consecutive days in a fed state with 2 subjects receiving matching placebo. The dose to be administered will be determined by the Sponsor after completion of the initial cohorts in subjects with genotype 1 CHC (no earlier than Cohort 2).<br><br>Cohort 6: Eight (8) CHC subjects (genotype 3) will be assigned to receive AL-335 given once daily on 7 consecutive days in a fed state with 2 subjects receiving matching placebo. The dose to be administered will be determined by the Sponsor after completion of the initial cohorts in subjects with genotype 1 CHC (no earlier than Cohort 2).<br><br>Cohort 7: Eight (8) CHC subjects (genotype 4-6) will be assigned to receive AL-335 given once daily on 7 consecutive days in a fed state with 2 subjects receiving matching placebo. The dose to be administered will be determined by the Sponsor after completion of the initial cohorts in subjects with genotype 1 CHC (no earlier than Cohort 2).<br><br>Cohort 8: Eight (8) CHC subjects (genotype 1 with documented compensated cirrhosis) will be assigned to receive AL-335 given once daily on 7 consecutive days in a fed state with 2 subjects receiving matching placebo. The dose to be administered will be determined by the Sponsor after completion of the initial cohorts in subjects with genotype 1 CHC (no earlier than Cohort 2). Sequence analysis of HCV NS5b from CHC GT1 cohorts assigned to receive either AL-335 or placebo will be completed prior to enrollment of subjects with compensated cirrhosis.
**Predosing and Concomitant Medications:**	Nil

1.0 BACKGROUND AND RATIONALE

1.1 BACKGROUND INFORMATION

There are approximately 170 million people or 3% of the world's population infected with hepatitis C virus (HCV) (World Health Organization, WHO) with more than 365,000 deaths per year resulting from long-term complications (Perz et al. 2006). Chronic hepatitis C (CHC) is the most common chronic liver disease in the United States and Europe and is the leading cause for liver transplantation in these regions. CHC infection results in a significant risk of developing long-term complications such as cirrhosis and hepatocellular carcinoma (HCC), although estimates for the progression rates vary. The risk of hepatic decompensation in subjects with cirrhosis is approximately 5% per year (Poynard et al. 1997) and the 5-year survival rate after decompensation is around 50% (Planas et al. 2004).

The options for treating HCV infection are evolving rapidly. Following the approval of sofosbuvir (Sovaldi™ U.S. Prescribing Information, December 2013), the combination of pegylated interferon (Peg-IFN) and ribavirin (RBV) with sofosbuvir has become the standard of care for the treatment of genotype 1 CHC in the United States. The combination of sofosbuvir plus simeprevir with or without ribavirin is also recommended for individuals who are not eligible to receive IFN. For patients with HCV genotype 2 and 3, treatment with sofosbuvir and RBV is recommended (Recommendations for Testing, Managing, and Treating Hepatitis C).

Additional treatment strategies are being developed to replace PEG IFN and RBV with potent combinations of direct acting antiviral agents (DAA) that target different parts of the viral genome and life cycle. These combinations need to be more tolerable and result in high rates of sustained virological response (SVR) with shorter treatment durations. In addition, they should have broad genotype activity, minimal drug–drug interactions, high barriers to resistance, complimentary resistance profiles, and be capable of being used in fixed dose once daily orally administered combinations.

Nucleoside polymerase inhibitors have demonstrated the potential to become the backbone of DAA combinations due to their potential to provide a high-barrier to resistance, antiviral potency, activity against multiple genotypes and favorable clinical safety profile.

AL-335 is a uridine base nucleoside monophosphate prodrug (or nucleotide analog) being developed as an orally administered anti-HCV therapeutic. In the cell-based HCV replicon assay, AL-335 has demonstrated potent antiviral activity with an EC_{50} value of 75 nM. The mechanism of action of AL-335, mediated by its triphosphate derivative, ALS-022235, is through inhibition of NS5B, the HCV RNA-dependent RNA polymerase. ALS-022235 has been demonstrated to be a substrate for NS5B and after incorporation into the growing HCV RNA chain, acts as a chain terminator of further extension. AL-335 retains potent antiviral activity against most HCV replicons that show resistance to other direct acting antiviral agents. AL-335 is efficiently converted to ALS-022235 in human hepatocytes. In nonclinical studies, AL-335 is well absorbed and is extracted into the liver. In the liver,

metabolic conversion occurs leading to significant levels of the triphosphate derivative ALS-022235. ALS-022235 has demonstrated excellent selectivity and is not an inhibitor of human DNA or RNA polymerases, including the mitochondrial RNA polymerase. The half-life of ALS-022235 in human hepatocytes is approximately 31.4 hours and in vivo dog liver is 25.6 hours, making AL-335 suitable for once daily dosing. AL-335 has also demonstrated pan-genotypic activity in vitro, demonstrating similar potency across HCV replicons containing NS5B coding sequence derived from genotypes 1a, 1b, 2b, 3a, and 4a. Since AL-335 inhibits HCV polymerase with the same mechanism of action as sofosbuvir, which is active against genotypes 1-6 (Svarovskaiaet al. 2014; Lawitz et al. 2013), it is anticipated that AL-335 will also inhibit HCV GT5-6. Combination studies of AL-335 with other direct acting antiviral agents demonstrated additive to synergistic activity in vitro (see Investigators Brochure for more information).

AL-335 is metabolized by esterases. It has demonstrated a low inhibition potential to CYP1A2, CYP2B6, CYP2C8, CYP2C9, CYP2C19, CYP2D6 and CYP3A4 (IC_{50} > 26 μM). The metabolites, ALS-022399 and ALS-022227, did not show any inhibition to CYP2B6, CYP2C8 and CYP3A4, CYP450s that have been tested to date. AL-335 and metabolites are not expected to cause drug–drug interaction with other drugs that are metabolized by cytochrome P450 enzymes. However, as a precaution, subjects should not receive any medications known to be an inducer or inhibitor of liver enzymes within 3 weeks prior to receiving study medication until completing the study unless they receive treatment for CHC.

AL-335 was well tolerated in all toxicology studies. Following 14 days of repeated oral dosing, the target organs for toxicity were not identified even when dosed up to the maximum recommended doses of 1000 mg/kg/day in both rats and dogs. No significant changes in the hematology, serum chemistry or histopathological changes in either species when dosed for 14-day up to 1000 mg/kg/day, the highest dose tested. Other non-adverse, minimal, non-physiologically relevant changes reported include slight increase in heart rate (~10%) and body temperature (< 1°C) in the dog cardiovascular study following single dose of 1000 mg/kg dose. The 1000 mg/kg/day was considered the no-observed-adverse-effect-level (NOAEL) in both rats and dogs, where highest steady state AUC_{0-24} for AL-335, ALS-022399 and ALS-022227 were 2490, 22900 and 75500 ng·h/mL, respectively.

Infrequent but serious adverse events associated with mitochondrial toxicity, such as myopathy, lactic acidosis, hepatomegaly with steatosis, neuropathy, and pancreatitis, have been observed with chronic administration of nucleos(t)ide analogs for the treatment of HIV infection (Lewis and Dalakas 1995) and chronic hepatitis B (Fontana 2009). The risk of developing any of these abnormalities in the phase 1 program is considered to be very low as AL-335 did not exhibit mitochondrial toxicity in vitro and the durations of dosing in the phase 1 studies is short.

As of 10 April 2015, forty (40) healthy volunteers have received single doses up to 1200 mg (SAD) or two 400 mg doses (Part 2) of study medication. There were no SAEs, and no clinically relevant laboratory, ECG, Holter, vital sign, or physical exam safety signals have

been identified. Three subjects reported four adverse events (AEs), one of which emerged prior to dose administration. For the treatment emergent adverse events (TEAE), one subject experienced mild intermittent palpitations 72 hours after dosing. The event did not require concomitant medication or other intervention, was not associated with objective evidence of tachycardia, and was deemed unlikely to be related to study drug. The second TEAE, moderate dental pain (2 events) , occurred 9 hours post-dose and again 6 days after dosing. These events were assessed as unrelated to study drug.

In Part 3 (MAD), twenty (20) subjects with genotype 1 CHC infection have completed the study, each receiving 7 days of dosing with study medication. Sixteen (16) received active study medication (400 or 800 mg AL-335) and four (4) received placebo. A total of 17 adverse events have been reported after initiation of dosing. The most commonly reported adverse events (≥2 events reported) were headache (8 events), increased creatine kinase (2 events), elevated ALT (2 events) and elevated AST (2 events). The ALT and AST elevations each occurred in two subjects ≥10 days after the conclusion of dosing and were similar in magnitude to baseline (pre-dosing) levels. Three adverse events occurred one time each: fatigue, common cold, and increased bilirubin. All AEs were mild (8 events) or moderate (8 events) in severity with the exception of one event of elevated creatine kinase (CK) which was considered severe but not serious. This subject was asymptomatic and had a history of elevated CK levels related to body building activities. His CK levels peaked at Day 3 of dosing and then declined despite continuing dosing through study completion (Day 7). Including the laboratory-based AEs described above, no clinically relevant laboratory, ECG, Holter, vital sign, or physical exam safety signals have been identified to date.

Preliminary pharmacokinetic (PK) data from Part 1 (SAD) demonstrate that plasma exposures of AL-335 and its metabolites ALS-022399 and ALS-022227 (parent nucleoside), increased linearly in an approximately dose proportional manner up to 800 mg and remained below the protocol mandated limits. Upon administration of AL-335 with high fat food, delayed T_{max} was noted for all three analytes. A slight increase in AL-335 and ALS-022399 AUC was observed with no significant changes in $C_{max.}$ Food did not affect C_{max} or AUC of ALS-022227. Inter subject variability in plasma exposure for 3 analytes were reduced when AL-335 was administered with food. These data suggest there was no significant or clinically relevant food effect observed and dosing in subsequent cohorts may proceed with or without food.

Additional information regarding AL-335 is available in the AL-335 Investigator’s Brochure.

1.2 RATIONALE FOR THE STUDY

This study is being performed to determine preliminary safety and PK data after single doses of AL-335 given in a fed and fasted state to HVs. Subsequently, the safety, pharmacokinetics, short-term antiviral efficacy, and resistance profile of AL-335 will be evaluated in treatment-naïve subjects with CHC genotype 1 infection. After an initial evaluation of the safety and efficacy of AL-335 in subjects with genotype 1 CHC infection, additional cohorts of subjects with genotype 2-6 CHC infection will be evaluated. In

addition a cohort of subjects with genotype 1 CHC infection and compensated cirrhosis will be evaluated to determine the safety, efficacy and PK profile in a population with more advanced liver disease. The inclusion of an oral suspension formulation will allow for an evaluation of the performance of the tablet formulation to determine if additional improvements in the formulation are required.

1.3 RATIONALE FOR DOSE SELECTION

A series of nonclinical toxicology, safety pharmacology, and genotoxicity studies have been conducted which support the initiation of clinical trials in HV and subjects with CHC. The dose rationale is based on the projected human efficacious doses. The starting doses in the SAD and MAD are based on safety margins derived from the NOAELs that were obtained in the 14-day repeated dose toxicology studies in rats and dogs (see Table 1.3-1 and Table 1.3-2).

The projected human efficacious dose for AL-335 is 50–650 mg, once daily (qd). These projections are primarily based on formation of the nucleoside triphosphate (NTP), the active entity (ALS-022235), in the in vitro and in vivo assays. Due to similarity of the stability of the pro-drug, AL-335, in dogs and human in vitro plasma and liver S9 stability assays, the human dose projection assumes that the absorption, pharmacokinetics and rate and extent of formation of NTP in humans will be similar to dogs. In dogs, a steady state NTP C_{min} of 6.8 µM was formed in the liver following an oral dose of 10 mg/kg. Thus, in order to maintain a C_{min} at or above the K_i for inhibition of NS5B polymerase, NTP levels at EC_{50} and EC_{90} of inhibition of HCV replication in Huh 7 cells, the doses required in the dog range from 0.06–19 mg/kg. The human equivalent dose (HED) is 0.03–10.5 mg/kg or 1.8 to 630 mg for a 60 kg human (CDER Guidance, 2005). The once daily dosing regimen is based on the fact that the NTP has a long half-life of 25.5 hours in dog liver following single low oral dose of AL-335.

Table 1.3-1. Parameters Used in Determining Human Projected Efficacious Doses

K_i for NS5B polymerase	0.024 µM
NTP levels at EC_{50}	2.6 µM
NTP levels at EC_{90}	6.8 µM
Steady State C_{min} liver NTP levels in dogs at 10 mg/kg, oral with tablets	6.8 µM
Oral dose range to maintain C_{min} NTP levels at K_i, EC_{50} or EC_{90}	
Dog: 0.06 -19 mg/kg	Human equivalent dose (HED)*: 0.03 - 10.5 mg/kg<br>or<br>1.8 -630 mg for 60 kg human

*Conversion factor of 1.8 for dogs (FDA Guidance for Industry, Estimating the maximum safe starting dose in initial clinical trials for therapeutics in adult healthy volunteers, July 2005).

Table 1.3-2. Safety Margin and Starting Doses per FDA Guidelines

Safety Factor	Maximum Starting Dose	
	Based on NOAEL in Dog: 1000 mg/kg (HED = 33,333 mg) [a]	Based on NOAEL in Rat: 1000 mg/kg (HED = 9999 mg) [a]
10-Fold (FDA recommended)	3333 mg	999 mg
300-Fold (dog)	100 mg	-
100-Fold (rat)	-	100 mg

HED: human-equivalent dose; NOAEL: no-observed-adverse-effect level from 14-day repeat dose toxicity studies.
[a] Conversion factor of 1.8 for dogs, 6.2 for rats, and weight of human 60 kg

The proposed starting dose of 100 mg in the SAD is at least 100-fold lower than the NOAEL HEDs (see Table 1.3-2) providing a large safety margin. The planned dose escalation of 2- to 3-fold between doses in the SAD will establish the dose response relationship of PK parameters. The proposed top dose of 1200 mg is approximately 2 times the projected efficacious dose range and remains at least 10 fold lower than the NOAEL HEDs in the most sensitive species (rat; Table 1.3-2).

In the MAD the proposed starting dose of 400 mg is at least 25-fold lower than the NOAEL HEDs and within the projected human efficacious dose range. The proposed top dose of 600 mg is within the projected efficacious dose range and remains at least 16 fold lower than the NOAEL HEDs in the most sensitive species (rat; Table 1.3-2). The proposed dose escalations will allow evaluation of dose response relationship for PK and viral kinetics. Before each dose escalation, a review of the safety parameters will be conducted. Doses will not be escalated beyond the highest mean plasma exposures of the parent nucleoside, ALS-022227, that were obtained in toxicology studies conducted in the dog, where mean AUC_{0-24} was75500 ng·h/mL. See AL-335 Investigator's Brochure for more information regarding the toxicology studies.

2.0 STUDY DESIGN

2.1 SUMMARY

This randomized, double-blind, placebo-controlled, 3-part study will assess the safety, tolerability, and pharmacokinetics of orally administered AL-335 in healthy volunteers (HV) and subjects with CHC infection.

Part 1: HV will receive one of 5 single ascending doses (SAD) of AL-335 ranging from 100 mg to 1200 mg. Within each cohort subjects will be randomized to receive either AL-335 or placebo (n=8 per cohort; 6 assigned to AL-335 and 2 assigned to placebo), in a fasted state. Up to three additional cohorts may be evaluated at the discretion of the Sponsor and

Principal Investigator (PI) based on the emerging PK profile and the presence of an acceptable safety profile as outlined in Section 5.1.1. The additional cohorts may be administered an oral suspension or tablet formulation of AL-335 corresponding to one or more of the planned dose levels or to evaluate a higher dose. These doses will be evaluated under fed conditions.

Part 2: Eight HV from Cohort 3 in Part 1 will receive a second single dose of AL-335 or placebo (with a washout period of 11 to 21 days) to assess food effects on pharmacokinetics.

Part 3: The following cohorts of 10 subjects with CHC infection will be evaluated.

Subjects with CHC genotype 1 infection will receive one of 3 ascending doses of AL-335 (400 mg, 600 mg, or 800 mg) or placebo dosed once daily for 7 days (n=10 per cohort, 8 assigned to AL-335 and 2 assigned to placebo). Based on the emerging data, an additional cohort of 10 subjects with CHC genotype 1 infection may be enrolled to evaluate an alternative dose of AL-335.

The following cohorts may be enrolled after a review of the safety and efficacy of the initial MAD cohorts which will be conducted by the sponsor in consultation with the principal investigators. The dose to be administered will have demonstrated at least a mean 3 $\log_{10}$ reduction in HCV RNA concentrations in a prior cohort of genotype 1 subjects in combination with an acceptable safety and PK profile.

- Genotype 2: a cohort of ten (10) treatment naïve subjects with genotype 2 CHC infection will be enrolled (8 assigned to AL-335 and 2 assigned to placebo) to evaluate AL-335 dosed once daily for 7 days.
- Genotype 3: a cohort of ten (10) treatment naïve subjects with genotype 3 CHC infection will be enrolled (8 assigned to AL-335 and 2 assigned to placebo) to evaluate AL-335 dosed once daily for 7 days.
- Genotypes 4-6: a cohort of ten (10) treatment naïve subjects with genotypes 4-6 CHC infection will be enrolled (8 assigned to AL-335 and 2 assigned to placebo) to evaluate AL-335 dosed once daily for 7 days.
- Compensated cirrhosis: a cohort of ten (10) subjects with CHC genotype 1 infection and documented compensated cirrhosis (Child-Pugh Class A) will be enrolled (8 assigned to AL-335 and 2 assigned to placebo) to evaluate AL-335 dosed once daily for 7 days. Sequence analysis of HCV NS5b from CHC GT1 cohorts assigned to receive either AL-335 or placebo will be completed prior to enrollment of subjects with compensated cirrhosis and CHC GT1.

In Parts 1–2, the doses to be evaluated may be modified based on emerging PK and safety data. However, there will be no more than a three-fold increase in dose per cohort (see *Cohort Progression Guidelines*, Section 5.1.1) and the mean exposure for ALS-022227 (AUC_{0-24}) at a dose level will be projected not to exceed 75,500 ng·h/mL.

In Part 3, the doses to be evaluated may be modified based on emerging PK, HCV RNA and

safety data. However, the maximum dose administered will not exceed the maximum tolerated dose observed in Part 1; the mean exposure for ALS-022227 (AUC_{0-24}) at a dose level will be projected not to exceed 75,500 ng·h/mL; and there will be no more than a three-fold increase in dose per cohort (see *Cohort Progression Guidelines*, Section 5.1.1).

In Parts 1–3, an increase in dose will only occur if the previous dose is demonstrated to be generally safe and tolerable (see *Cohort Progression Guidelines*, Section 5.1.1). Before the second and subsequent cohorts are conducted, safety data from the prior cohort will be reviewed by the Principal Investigator (PI) and Sponsor.

Part 3 will commence when the third dose of Part 1 is found to be safe and tolerable based upon a review of the blinded safety data by the PI and the Sponsor. Dosing will take place after a standard meal.

Safety and tolerability will be evaluated on an ongoing basis through assessment of adverse events, blood and urine sample analyses, collection of electrocardiograms (ECG), vital signs and physical examinations. In the event a safety signal is detected, the pharmacokinetic/pharmacodynamic (PK/PD) relationship between AL-335 and its metabolites and various safety parameters including ECG changes, vital signs, and relevant laboratory parameters will be evaluated.

The Sponsor will provide access to components of Standard of Care for subjects with CHC participating in Part 3 who are unable to access it locally, through an appropriate mechanism for each participating country subject to local regulations. To be eligible, subjects with CHC must complete the study and commence treatment within 6 months of the study completion visit.

See Section 6.0 for Study Procedures.

2.2 STUDY SCHEMA

The dosing schema is presented below. Study procedures are tabulated in Section 6.1, *Schedule of Events*.

Figure 2.2-1. Dosing Schema

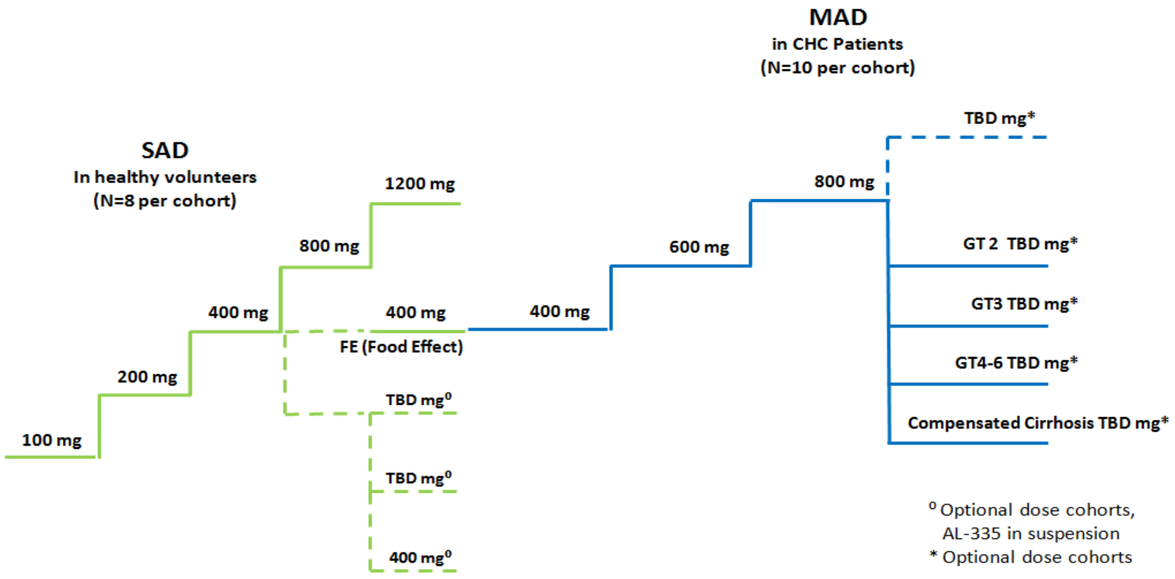

3.0 <u>STUDY OBJECTIVES AND ENDPOINTS</u>

3.1 STUDY OBJECTIVES

3.1.1 Primary Objective

To evaluate the safety and tolerability of single and multiple doses of AL-335 administered to HV and in subjects with CHC infection, respectively

3.1.2 Secondary Objectives

To evaluate, after single and multiple doses of AL-335, the pharmacokinetics of AL-335, ALS-022399 and ALS-022227 (and other metabolites if applicable) in plasma and urine (single doses only)

To evaluate, after a single dose of AL-335, the effect of dosage formulation (tablet versus suspension) on the pharmacokinetics of AL-335, ALS-022399 and ALS-022227 (and other metabolites if applicable) in plasma

To evaluate the effect of food intake on the pharmacokinetics of AL-335, ALS-022399 and ALS-022227 (and other metabolites if applicable)

To evaluate the viral kinetics of HCV RNA in subjects with CHC infection treated with AL-335

To evaluate the viral resistance profile after 7 daily doses of AL-335 in subjects with CHC infection

To characterize the relationship between plasma exposures of AL-335 and/or its metabolites and viral kinetics

3.2 STUDY ENDPOINTS

3.2.1 Primary Endpoints

- Safety data including but not limited to treatment emergent adverse events, physical examination findings, vital signs, 12-lead ECG and clinical lab results (including chemistry, hematology, and urine)

3.2.2 Secondary Endpoints

- PK parameters of AL-335, ALS-022399, ALS-022227 (and other metabolites if applicable) following single dose administration: C_{max}, t_{max}, $t_{1/2}$, CL/F and V_z/F (for AL-335 only), $AUC_{0\text{-}inf}$ or AUC_{last}
- PK parameters of AL-335, ALS-022399, ALS-022227 (and other metabolites if applicable) following repeat dose administration: C_{max}, t_{max}, $t_{1/2}$, AUC_{last} and $AUC_{0\text{-}tau}$
- PK parameters of AL-335, ALS-022399, ALS-022227 (and other metabolites if applicable) following single dose administration in tablet form as compared with suspension: C_{max}, t_{max}, $t_{1/2}$, CL/F and V_z/F (for AL-335 only), $AUC_{0\text{-}inf}$ or AUC_{last}
- PK parameters of AL-335 (and metabolites if applicable) after a single oral dose in HV in fasted conditions as compared with fed conditions
- Concentration in urine and urinary excretion of AL-335, ALS-022399, ALS-022227 (and other metabolites if applicable) after a single oral dose in HV in fasted conditions
- HCV ribonucleic acid (RNA) viral load change from baseline in subjects with CHC infection
- Sequence analysis of the HCV NS5B region in subjects with CHC infection, as appropriate

4.0 SELECTION AND WITHDRAWAL OF SUBJECTS

4.1 STUDY POPULATION

Approximately 40 HV (n=8 per cohort) will receive one dose of AL-335 or placebo in Part 1 (SAD). An additional 24 HV may be enrolled into three additional dose cohorts at the discretion of the PI and Sponsor. In Part 2 (Food Effect), approximately 8 HV (Cohort 3 from Part 1) will receive one dose of AL-335 or placebo on two occasions, one initially in a fasted state and one in a fed state, separated by an 11 to 21 day washout. In Part 3 (MAD), approximately 70 (up to 80) subjects with CHC infection will be enrolled to receive 7 days of dosing with AL-335 or placebo.

4.2 INCLUSION CRITERIA

Main Inclusion Criteria for All Subjects:

1. Subject has provided written consent.
2. In the investigator's opinion, the subject is able to understand and comply with protocol requirements, instructions, and protocol-stated restrictions and is likely to complete the study as planned.
3. Subject is in good health as deemed by the investigator, based on the findings of a medical evaluation including medical history, physical examination, laboratory tests, and ECG.
4. Male or female, 18–60 years of age for HV and 18–65 years of age for subjects with CHC.
5. Body mass index (BMI) 18–32 kg/m^2 inclusive for HV and 18–35 kg/m^2 for subjects with CHC, minimum weight is 50 kg in both populations. No more than 25% of patients in any cohort may be enrolled with a BMI ≥ 30 kg/m^2.
6. A female subject is eligible to participate in this study if she is of non-childbearing potential (defined as females with a documented tubal ligation, bilateral oophorectomy, or hysterectomy) or postmenopausal (defined as 12 months of spontaneous amenorrhea and follicle stimulating hormone (FSH) level within the laboratory's reference range for postmenopausal females). A postmenopausal female receiving hormone replacement therapy who is willing to discontinue hormone therapy 28 days before study drug dosing and agrees to remain off hormone replacement therapy for the duration of the study may be eligible for study participation.
7. If male, subject is surgically sterile or practicing specific forms of birth control (as outline in Section 6.2.9) until 90 days after the end of the study.

Additional inclusion criteria for subjects with CHC infection:

8. Documentation of HCV infection for greater than 6 months at dosing
9. Screening HCV RNA viral load ≥10^5 IU/mL using a sensitive quantitative assay, such as COBAS® Taqman® HCV Test 2.0, except for subjects with compensated cirrhosis (Child-Pugh Class A) who may have HCV RNA viral load ≥10^4 and ≤10^8 IU/mL
10. In addition, subjects with compensated cirrhosis and CHC genotype 1 infection and must meet the Child-Pugh Class A definition (see Appendix F) and at least one of the following criteria:
    - Liver biopsy result within 6 months of Day 1 indicating the presence of cirrhosis (e.g., Metavir F4; Ishak > 5) or
    - Fibroscan evaluation within 3 months of Day 1 with a liver stiffness score ≥ 14.5 kPa

4.3 EXCLUSION CRITERIA

Subjects will be ineligible for this study if they meet **any one** of the following criteria:

1. Clinically significant cardiovascular, respiratory, renal, gastrointestinal, hematologic, neurologic, thyroid, or any other medical illness or psychiatric disorder, as determined by the Investigator and/or Medical Monitor.
2. Positive test for HAV IgM, HBsAg, or HIV antibody. In Parts 1 and 2, positive HCV serology is exclusionary.
3. Any condition that, in the opinion of the investigator, would compromise the study's objectives or the well-being of the subject or prevent the subject from meeting the study requirements.
4. Participation in an investigational drug trial or having received an investigational vaccine within 30 days or 5 half-lives (whichever is longer) prior to study medication
5. Clinically significant abnormal ECG findings. Particularly, a history or family history of prolonged QT syndrome (e.g., torsade de pointes) or sudden cardiac death; or a corrected QT interval (QTc) > 450 milliseconds for male subjects and > 470 milliseconds for female subjects at the Screening Visit.
6. Clinically significant blood loss or elective blood donation of significant volume (i.e., > 500 mL) within 60 days of first dose of study drug; > 1 unit of plasma within 7 days of first dose of study drug
7. Abnormal heart rate, respiratory rate, temperature or blood pressure values outside of the normal range (evaluated in a semi-recumbent or recumbent position after 5 minutes of rest). One repeat measurement after an additional 5 minutes of rest is permitted.
8. Evidence of active infection (other than CHC infection in subjects enrolled in Part 3).
9. Unwilling to abstain from alcohol for 48 hours prior to the start of dosing through the study completion visit.
10. History of regular alcohol intake > 7 units per week of alcohol for females and > 14 units per week for males (one unit is defined as 10 g alcohol) within 3 months of screening visit.
11. For healthy subjects (Parts 1–2), history of regular use of tobacco (i.e., ≥ 10 cigarettes per day) or nicotine-containing products within 3 months of the screening visit. For subjects with CHC infection, history of regular use of tobacco- or nicotine-containing products is allowed.
12. The subject has a positive pre-study drug screen. A minimum list of drugs that will be screened for includes amphetamines, barbiturates, cocaine, opiates, cannabinoids, and benzodiazepines. Subjects with CHC may be included if they have a positive result for cannabinoids at screening. However, they must be willing to abstain from cannabinoid use throughout the duration of the study.

13. In Parts 1-2, the use of concomitant medications, including prescription, over the counter medications, or herbal medications within 14 days prior to the first dose of study medication is excluded, unless approved by the Sponsor's Medical Monitor. PRN use of a nonsteroidal anti-inflammatory drug (NSAID) is permitted.
14. For subjects in Part 3, the use of prescription and over the counter medications deemed necessary to maintain the health status of the subject are permitted, if approved by the Sponsor's Medical Monitor. PRN NSAID use is permitted. Further guidance for the use of prior medication in subjects with CHC can be found in Section 5.7.
15. Subjects must not have received any drug known to be a strong inducer or inhibitor of CYP450 enzymes within 2 weeks prior to study drug dosing (Appendix C).
16. Exposure to more than four new investigational entities within 12 months prior to the first dosing day.
17. Abnormal biochemistry or hematology laboratory results obtained at screening. Elevated bilirubin in subjects with suspected Gilbert's disease is allowed.

A subject with a clinical abnormality or laboratory parameters outside the reference range for the population being studied may be included if the Investigator determines the finding is unlikely to introduce additional risk factors and will not interfere with data interpretation. A single repeat laboratory evaluation is allowed for eligibility determination. The Investigator is encouraged to discuss any laboratory abnormalities which are considered potentially clinically significant with the Sponsor Medical Monitor prior to randomization.

Additional exclusion criteria for subjects with CHC infection:

18. History of clinical hepatic decompensation, e.g., variceal bleeding, spontaneous bacterial peritonitis, ascites, hepatic encephalopathy or active jaundice (within the last year)
19. For all MAD Cohorts (except subjects with compensated cirrhosis), a liver biopsy within two years or Fibroscan evaluation within 6 months prior to randomization that demonstrates cirrhosis (Knodell score > 3, Metavir score > 3, Ishak score > 4). Fibroscan liver stiffness score > 10.5 kPa.
20. Prior treatment for CHC with direct acting antiviral agents
21. Serum alanine aminotransferase (ALT) concentration > 5 x ULN
22. Evidence on screening liver ultrasound of hepatic mass or lesion concerning for malignancy.

4.4 SUBJECT SCREENING AND ENROLLMENT

Screening procedures will occur when a subject signs and dates an IRB/IEC/EC approved informed consent form (ICF) and provides authorization to use protected health information. The informed consent form will be completed prior to any study-specific procedures. For healthy volunteers screening criteria satisfied under a generic consent

within the screening window required by this study may be allowed at the sponsor's discretion. For subjects with CHC infection, screening procedures that are standard of care and have been undertaken prior to consent do not need to be repeated if they were conducted within the eligible screening period, unless otherwise specified.

To enroll, the investigator will verify eligibility according to all inclusion and exclusion criteria (Sections 4.2 and 4.3). For Parts 1, 2 and 3, eligible subjects will be assigned the next available subject number from a randomization list provided by the study statistician. Only the study pharmacist will have access to the corresponding treatment assignment. Any questions regarding eligibility should be addressed to the Sponsor's Medical Monitor prior to randomization.

4.5 SUBJECT DISCONTINUATION

Subjects are free to discontinue their participation at any time during this clinical trial. The investigator has the right to remove any subject from treatment with study drug or participation in the study. However, Alios requests that the investigator consult with the Sponsor's Medical Monitor before prematurely removing a subject. For subjects that discontinue study drug prematurely, sites are encouraged to follow these subjects as prescribed in this protocol as much as possible as this is the primary mechanism to ensure subject safety.

Any subject who decides to discontinue participation in the study, or meets the discontinuation criteria specified below, should undergo early discontinuation procedures (Section 4.5.2). Subjects that discontinue for safety reasons will not be replaced.

4.5.1 Subject Discontinuation Criteria

The primary consideration in any determination to discontinue a subject's participation must be the health and welfare of the subject. Reasons for discontinuation may include, but are not limited, to the following:

- An adverse or serious adverse event (AE/SAE), drug reaction, or complication, whether related or not to study drug, which precludes continuation of treatment with study drug. This includes the development of allergic reactions or other potentially serious drug reactions to the study medication.
- Noncompliance with study drug dosing.
- Noncompliance with study procedures.
- Lost to follow-up.
- Subject's right to withdraw consent at any time during the study, with or without a stated reason.
- The principal investigator's opinion that it is not in the subject's best interest to continue study participation.
- Sponsor's decision to terminate the study.

4.5.2 Procedures for Subjects Who Discontinued

Subjects who discontinue from treatment should undergo the Study Completion evaluations tabulated in Section 6.1 for the purpose of safety monitoring within 7 days after their last dose of study medication. Any subject that discontinues with ongoing AEs should be followed until resolution of their AE(s) or until the PI has determined that the AE(s) has stabilized.

4.5.3 Documentation of Withdrawal of Subjects

Document the reasons for early discontinuation of any subject from the study on the appropriate case report form (CRF). If the reason for early discontinuation is an AE or an abnormal laboratory value, record the specific event or test result on the AE CRF, and monitor the subject until the event is resolved or deemed stable by the investigator.

4.6 STUDY DISCONTINUATION

The sponsor has the right to terminate this study or remove a participating site at any time. Reasons for terminating the study or site may include, but are not limited to, the following:

- The incidence or severity of AEs in this or other studies indicates a potential health hazard to subjects.
- Subject enrollment is unsatisfactory.
- Data recording is inaccurate or incomplete.
- Investigator does not adhere to the protocol or applicable regulatory guidelines in conducting the study.
- A decision from the Institutional Review Board (IRB)/Independent Ethics Committee (IEC), or regulatory authority to terminate the study.

5.0 TREATMENT OF SUBJECTS

5.1 TREATMENT REGIMENS

The treatment regimens through study completion are shown in Table 5.1-1.

Table 5.1-1. Dosing Regimen

Cohort No.	Study Drug Dose Level and Frequency
**Part 1**	
1	6 subjects to receive a single oral 100-mg dose of AL-335 in a fasted state with 2 subjects receiving matching placebo
2	6 subjects to receive a single oral 200-mg dose of AL-335 in a fasted state with 2 subjects receiving matching placebo
3	6 subjects to receive a single oral 400-mg dose of AL-335 in a fasted state with 2 subjects receiving matching placebo

Cohort No.	Study Drug Dose Level and Frequency
4	6 subjects to receive a single oral 800-mg dose of AL-335 in a fasted state with 2 subjects receiving matching placebo
5	6 subjects to receive a single oral 1,200-mg dose of AL-335 in a fasted state with 2 subjects receiving matching placebo
6	6 subjects to receive single 400 mg oral dose of AL-335 (oral suspension formulation) in a fed state, with 2 subjects receiving matching placebo
7, 8	Two additional dose cohorts of AL-335 may be enrolled at the discretion of the Sponsor and the PI based on the emerging PK profile and the presence of an acceptable safety profile. Per cohort, six (6) subjects will receive single oral dose of AL-335 in a fed or fasted state at a dose to be determined and administered as either a tablet or oral suspension, with 2 subjects receiving matching placebo
**Part 2**	
	8 subjects who received the 3rd dose fasted in Part 1 (Cohort 3) will also receive AL-335 or placebo in fed (high fat meal) conditions after an 11-21-day washout period.
**Part 3**	
1	8 CHC subjects (genotype 1) will be assigned to receive 400 mg of AL-335 given once daily on 7 consecutive days with 2 subjects receiving matching placebo in a fed state.
2	8 CHC subjects (genotype 1) will be assigned to receive 600 mg of AL-335 given once daily on 7 consecutive days with 2 subjects receiving matching placebo in a fed state.
3	8 CHC subjects (genotype 1) will be assigned to receive 800 mg of AL-335 given once daily on 7 consecutive days with 2 subjects receiving matching placebo in a fed state.
4	(Optional) 8 CHC subjects (genotype 1) will be assigned to receive AL-335 given once daily on 7 consecutive days in a fed state with 2 subjects receiving matching placebo. The dose to be administered will be determined by the Sponsor after completion of Cohort 3.
5	8 CHC subjects (genotype 2) will be assigned to receive AL-335 given once daily on 7 consecutive days with 2 subjects receiving matching placebo in a fed state. The dose to be administered will be determined by the Sponsor after completion of the initial cohorts in subjects with genotype 1 CHC (no earlier than Cohort 2).
6	8 CHC subjects (genotype 3) will be assigned to receive AL-335 given once daily on 7 consecutive days with 2 subjects receiving matching placebo in a fed state. The dose to be administered will be determined by the Sponsor after completion of the initial cohorts in subjects with genotype 1 CHC (no earlier than Cohort 2).
7	8 CHC subjects (genotype 4-6) will be assigned to receive AL-335 given once daily on 7 consecutive days with 2 subjects receiving matching placebo in a fed state. The dose to be administered will be determined by the Sponsor after completion of the initial cohorts in subjects with genotype 1 CHC (no earlier than Cohort 2).
8	8 CHC subjects (genotype 1) with compensated cirrhosis (Child-Pugh A) will be assigned to receive AL-335 given once daily on 7 consecutive days with 2 subjects receiving matching placebo in a fed state. The dose to be administered will be determined by the Sponsor after completion of the initial cohorts in subjects with genotype 1 CHC (no earlier than Cohort 2). Sequence analysis of HCV NS5b from CHC GT1 cohorts assigned to receive either AL-335 or placebo will be completed prior to enrollment of subjects with compensated cirrhosis and CHC GT1.

5.1.1 Cohort Progression Guidelines

For Part 1 (SAD), the decision to proceed to subsequent dosing cohorts will be made by the

Sponsor in consultation with the PI based on a blinded review of the emerging safety data and PK data, if available. Three additional dose cohorts may be enrolled at the discretion of the Principal Investigator (PI) and Sponsor based on the emerging PK profile and the presence of an acceptable safety profile. The dose(s) to be administered and the form of administration (tablet or oral suspension) will be determined by the sponsor. The planned dose escalation schema may be amended based on the emerging PK and safety data. However, the maximum dose administered will not exceed 1500 mg or the maximum mean plasma exposures that were obtained for ALS-022227 in toxicology studies conducted in the dog (i.e., an $AUC_{0\text{-}24}$ > 75500 ng·h/mL). In addition, there will be no more than a three-fold increase in dose per cohort. For the 3rd and subsequent cohorts, dose escalation cannot proceed without review of the PK data from two dose levels below the next cohort.

For Part 3 (MAD), the decision to proceed to subsequent dosing cohorts will be made by the Sponsor in consultation with the PI based on a blinded review of clinical safety data through Day 9. In addition, during Part 3, representatives of the Sponsor who will have no direct involvement in the day to day activities of the study will review the HCV RNA data to confirm the dose to be evaluated for the next cohort. Subsequent dose levels may be amended on the basis of the emerging safety, PK and/or HCV RNA data. In the presence of an acceptable safety profile, the dose to be evaluated in the next cohort may be increased if the data suggest that improvements in either PK or HCV RNA concentrations are still possible. Alternatively, the dose to be evaluated in the next cohort may be decreased if the data suggest that there is value in exploring a lower intermediary dose. However, the highest dose level evaluated will not exceed the maximum tolerated dose in Part 1 or the maximum mean plasma exposures that were obtained for ALS-022227 in toxicology studies conducted in the dog (i.e., an $AUC_{0\text{-}24}$ > 75500 ng·h/mL). In addition, there will be no more than a three-fold increase in dose per cohort.

All 8 healthy volunteers or 8 of the 10 subjects with CHC in each cohort must complete the dosing schedule before a decision is made to proceed to subsequent dosing cohorts. Additional subjects may be recruited to evaluate a dose further if the data obtained from the initial cohort is not sufficiently robust for any given dose level. A decision to replace subjects for reasons other than safety-related will be made by the Sponsor in consultation with the PI.

Dose escalation will be suspended:

- If 4 or more subjects in a dose cohort experience moderate AEs or toxicities considered possibly or probably related to study drug, OR
- If 2 or more subjects in a dose cohort experience severe AEs or toxicities considered possibly or probably related to study drug, OR

- If one or more subjects in a dose cohort experience life threatening AEs or toxicities considered possibly or probably related to study drug. In this case, the Sponsor's Medical Monitor will review the treatment allocation to determine if the subject is receiving study medication or placebo. If the subject was receiving placebo then dose escalation may recommence, OR
- If 3 or more subjects in a dose cohort experience a sustained QTcF greater than 500 msec associated with an increase greater than 60 msec , OR
- If 3 or more subjects in a dose cohort experience a sustained resting heart rate of greater than 100 beats per minute

If dose escalation is suspended, a more comprehensive review of the safety data will be undertaken by the Sponsor and the PI. Based on this review, a decision will be made to continue with dose escalation, repeat a previously evaluated dose, evaluate a lower dose, modify the protocol, or terminate the study.

5.2 DESCRIPTION OF STUDY DRUG AND BACKGROUND THERAPY

5.2.1 Study Drug

Study drug will be supplied as tablets or in suspension. Study drug or placebo will be administered to subjects in a blinded fashion. The site pharmacist will be responsible for ensuring that treatment allocation information is not shared with other site personnel. Study drug and placebo will be supplied to the site in open-label containers and stored at controlled room temperature.

5.3 DOSE PREPARATION AND ADMINISTRATION

Study drug must be prepared by the licensed investigational pharmacist or other authorized personnel with appropriate training. The PI, sub-investigator, or PI designated health professional trained in managing allergic reactions, such as anaphylaxis, should be available during and after study drug administration. An emergency cart and medications must also be readily available.

AL-335 will be supplied as tablets or as a powder in amber bottles (10 g) for reconstitution as a suspension.

AL-335 tablets will contain 25 or 100 mg of AL-335 and microcrystalline cellulose (filler), croscarmellose sodium (disintegrant), and magnesium stearate (lubricant).

The matching placebo tablets will consist of lactose monohydrate (filler), microcrystalline cellulose (filler), croscarmellose sodium (disintegrant), and magnesium stearate (lubricant).

AL-335 powder will be mixed with methylcellulose (400cp) to create an oral suspension.

Dosing will be staggered to avoid having all of the subjects on the same exact study schedule. For subjects participating in Parts 2 or 3, administration of study medication should occur at the same time of day as the first dose. The exact time of dosing will be recorded in the source documents and the CRFs.

Each dose of study drug or placebo will be administered orally with approximately 250 mL of room temperature water to drink. A mouth check will be performed to ensure that the tablet was swallowed.

For additional instructions on preparation and administration of study drug, refer to the Pharmacy Manual.

5.4 ORDERING STUDY DRUG

To order study drug, refer to the Pharmacy Manual.

5.5 DRUG ACCOUNTABILITY

Study site personnel will maintain adequate records of the receipt and disposition of all study medication shipped to the site. Records must include dates, lot numbers, quantities received, quantities dispensed, date and time of preparation, date and time of administration, and the identification number of each subject who has received each lot of study drug.

The investigator or designee will administer study drug only to subjects enrolled in this protocol. The investigator will not supply study drug to any person not authorized to receive it.

5.6 DISPOSITION OF USED, PARTIALLY USED, AND UNUSED STUDY MEDICATION CONTAINERS

All used and unused study drug supplied by Alios BioPharma must be retained by the pharmacist. Periodically throughout and at the conclusion (or suspension, termination, or discontinuation) of the study, an unblinded representative of Alios BioPharma or its designated agent will conduct inventories and accountability of study materials. Once accountability is completed, an Alios BioPharma representative or designee will authorize the return of all used and unused study medication containers to a designated facility. For study medication containers returned to Alios BioPharma or its designated agent, records will include dates, lot numbers, and quantities of study drug returned. No local destruction of study drug is permitted.

5.7 CONCOMITANT MEDICATIONS

For Parts 1–2, from the time of admission to the Phase 1 Unit until the end of study, concomitant medications, including prescription, over the counter, and herbal medications, are prohibited with the exception of NSAIDS (e.g., ibuprofen) and medications required to ensure patient safety (See Prohibited Medications). The investigator is required to discuss the use of concomitant medications with the Sponsor’s Medical Monitor prior to initiation of any medications unless the subject requires immediate medical attention. Record **all** concomitant medications and supportive therapy from the date the informed consent is signed through the final study visit in the source documentation.

In Part 3, only those concomitant medications deemed necessary to maintain the subject's health status (e.g., chronic prescription medications), NSAIDs (on a PRN basis), or medications to ensure patient safety are permitted (See Prohibited Medications). The investigator is required to discuss the use of concomitant medications with the Sponsor's Medical Monitor prior to initiation of any medications unless the subject requires immediate medical attention. Record **all** concomitant medications and supportive therapy from the date the informed consent is signed through the final study visit in the source documentation.

5.8 PROHIBITED MEDICATIONS

In Parts 1–3, prohibited medications include sensitive or narrow CYP3A substrates or strong inducers and inhibitors of CYP3A (Appendix C). The use of over the counter, dietary, and herbal medications during study conduct is strongly discouraged unless their use can be demonstrated to be necessary to maintain a subject's current health status. The investigator must alert the Sponsor's Medical Monitor if a subject receives a prohibited medication.

Study treatment may be discontinued if a subject receives a prohibited medication. Subjects that discontinue study drug due to use of a prohibited medication will be followed up as outlined in Section 4.5.1, *Subject Discontinuation Criteria*.

6.0 STUDY PROCEDURES

6.1 SCHEDULE OF EVENTS

Subjects must receive the first dose of study drug within 21 days of the start of screening. Table 6.1-1 details the schedule of events for Part 1, the single ascending dose (SAD) phase. Table 6.1-2 details the schedule for Part 2, the food effect phase.

Subjects with CHC infection must receive the first dose of study drug within 28 days of screening. Table 6.1-3 details the schedule for Part 3, the multiple ascending dose (MAD) phase.

Table 6.1-1. Schedule of Events SAD Phase (Part 1)

Days / Assessments	Screen	Check-in				Check-out				Completion
	**-21 to -3**	**-2**	**-1**	**1**	**2**	**3**	**4**	**5**	**6**	**8**
Obtain informed consent before study procedures	X									
Confined to clinic		X	X	X	X	X				
Outpatient visits	X						X	X	X	X
Demographics	X									
Height, Weight, BMI	X									
Drug and alcohol screen	X	X								
Medical History	X	X (interim)								
Physical Exam [1]	X	X				X				X
Hepatitis and HIV screen (HBsAg, HBsAB, HBcAb, HCVAb, HIVAb, HAV IgM)	X									
Vital Signs [2]	X	X	X	X	X	X	X	X	X	X
Randomization				X						
**Study drug administration [3]**				**X**						
AE Evaluation	X---X									
PK Samples [4, 5]				X	X	X	X	X	X	X
Serum Chemistries, including ALT/AST [6]	X	X			X	X				X
CBC w/ differential including PT/PTT-INR plus Urinalysis	X	X			X	X				X
Pregnancy Test [7]	X	X								X
12-lead Holter Monitoring [8]			X	X						
12-Lead electrocardiogram [9]	X	X		X	X	X				X

1. Complete physical exam (PE) is to be done at screening and study completion. Symptom directed physical exams as indicated at other visits.
2. Vital signs (BP, HR, RR, body temperature) will be obtained in a semi-recumbent or recumbent position after 5 minutes of rest at Screening; Check-in (Day -2); Day -1; predose and at 1, 2, 4, 6 and 10 hours postdose on Day 1; 24 and 36 hours postdose; prior to clinic check-out (Day 3); and daily on Days 4, 5, 6 and 8. Vital signs will be conducted after ECG assessments and prior to PK collections.
3. Prior to each dose of study medication subjects will fast from food for ≥8 hours overnight until 4 hours postdose (SAD Cohorts 1-5). For the Optional SAD Cohorts (6-8), prior to each dose of study medication subjects will receive a standard meal and 250 mL of water. Except as part of dosing water may be

consumed freely except between 1 hour prior to and 2 hours after dosing. Subjects may be dosed in a staggered fashion to accommodate postdosing assessments. For subjects participating in Part 1 (SAD) & Part 2 (FE), dosing should occur at the same time each day +/- 5 minutes (e.g., subject 1 is dosed at 7:00AM on SAD Day 1 & FE Day 1, subject 2 is dosed at 7:05AM on SAD Day 1 & FE Day 1, etc.)

4. Plasma sample time-points: before dose administration; 0.25 and 0.5 and 1, 2, 3, 4, 6, 8, 12, 24 (Day 2), 36, 48 (Day 3), 72 (Day 4), 96 (Day 5), 120 (Day 6), and 168 hours (Day 8) postdosing.
5. Urine sample collection only (4th cohort only): -12–0, 0–6, 6–12, 12–24, 24–30, 30–36, 36–48, and 48–72, hours postdose.
6. Blood for routine safety evaluations should be taken in a fasted state.
7. Pregnancy testing can utilize urine or serum consistent with local standards.
8. 12-lead Holter monitoring will be conducted for 24 hours (+/- 1 hour) prior to dosing, and for 24 hours postdosing.
9. 12-lead ECGs will be obtained at Screening, Check-in, pre-dosing, 1.25, 3.5, 7 and 10 hours postdose on Day 1 and once on Days 2, 3, and 8. ECGs may be repeated at the discretion of the investigator to rule out erroneous readings. ECGs will be conducted immediately prior to PK sample collection and will be obtained in a semi-recumbent or recumbent position after 5 minutes of rest. Daily ECGs must be obtained at the same time each day (+/- 30 min).

Table 6.1-2. Schedule of Events for Food Effect Phase (Part 2)

Days	Screen	Check in				Check out					Check -in*				Check out				Completion
**Assessments**	**-21 to -3**	**-2**	**-1**	**1**	**2**	**3**	**4**	**5**	**6**	**8**	**-2**	**-1**	**+1**	**+2**	**+3**	**+4**	**+5**	**+6**	**+8**
Obtain informed consent before study procedures	X																		
Confined to clinic		X	X	X	X	X					X	X	X	X	X				
Outpatient visits	X						X	X	X	X						X	X	X	X
Demographics	X																		
Height, Weight, BMI	X																		
Drug and alcohol screen	X	X									X								
Medical History	X	X (interim)																	
Physical Exam [1]	X	X				X				X	X				X				X
Hepatitis and HIV screen (HBsAg, HBsAB, HBcAb, HCVAb, HIVAb, HAV IgM)	X																		
Vital Signs [2]	X	X	X	X	X	X	X	X	X	X	X	X	X	X	X	X	X	X	X
**Randomization**				**X**															
**Study drug administration [3]**				**X**									**X**						
**High-fat meal predose [3]**													**X**						
AE Evaluation	X--X																		
PK Samples [4,5]				X	X	X	X	X	X	X			X	X	X	X	X	X	X
Serum Chemistries w/ ALT & AST [6]	X	X			X	X				X	X			X	X				X
CBC w/differential including PT/PTT-INR and Urinalysis	X	X			X	X				X				X	X				X
Pregnancy Test [7]	X	X									X								X
12-lead Holter Monitoring [8]			X	X															
12-Lead ECG [9]	X	X		X	X	X				X	X	X	X	X	X				X

* Dose 2 must occur 11-21 days after Dose 1.

1. A complete physical exam (PE) is to be done at screening and at completion. Symptom directed PE as indicated at all other visits.
2. Vital signs (BP, HR, RR, body temperature) will be obtained in a semi-recumbent or recumbent position after 5 minutes of rest at the following intervals
    - Screening
    - Dose 1: Check-in (Day -2),Day -1, predose and at 1, 2, 4, 6 and 10 hours postdose on Day 1, 24 and 36 hours postdose (Day 2), prior to clinic check-out (Day 3) and on Days 4, 5, 6 and 8.
    - Dose 2: Check-in (Day-2), Day -1, predose and at 1, 2, 4, 6 and 10 hours postdose on Day +1, 24 and 36 hours postdose(Day +2), prior to clinic check-out (Day +3) and on Days +4, +5, +6 and +8.

    Vital signs will be conducted after ECG assessments and prior to PK collections.
3. Prior to Dose 1, subjects will fast from food for ≥8 hours overnight until 4 hours postdose. Except as part of dosing water may be consumed freely except between 1 hour prior to and 2 hours after dosing. Prior to Dose 2, subjects will consume a high fat meal as outlined in Protocol Section 6.2.1. Subjects may be dosed in a staggered fashion to accommodate postdosing assessments. For subjects participating in Part 1 (SAD) & Part 2 (FE), dosing should occur at the same time each day +/- 5 minutes (e.g., subject 1 is dosed at 7:00AM on SAD Day 1 & FE Day 1 and Day 11, subject 2 is dosed at 7:05AM on SAD Day 1 & FE Day 1 and Day 11, etc).
4. Plasma or whole blood sample time-points:

    Dose 1: predose administration, 0.25, 0.5, 1, 2, 3, 4, 6, 8, 12, 24 (Day 2), 36, 48 (Day 3), 72 (Day 4), 96 (Day 5), 120 (Day 6), and 168 hours (Day 8) postdosing

    Dose 2: predose administration, 0.25, 0.5, 1, 2, 3, 4, 6, 8, 12, 24 (Day+2), 36, 48 (Day +3), 72 (Day +4), 96 (Day +5), 120 (Day +6) and 168 hours (Day +8) post-2nd dose
5. No urine samples will be taken.
6. Blood for routine safety evaluations should be taken in a fasted state.
7. Pregnancy testing can utilize urine or serum consistent with local standards.
8. 12-lead Holter monitoring will be conducted for 24 hours (+/- 1 hour) prior to dosing, and for 24 hours postdosing (During Part 1 only).
9. 12-lead ECGs will be obtained for both dosing events. For the first dose, ECGs will be collected at Screening, Check-in, predose, 1.25, 3.5, 7 and 10 hours postdose on Day 1, and once on Days 2, 3, and 8. For the second dose, ECGs will be collected at Check-in, predose, 1.25, 3.5, 7 and 10 hours postdose on Day +1, once on Days +2, +3 and at the completion visit. ECGs will be obtained in a semi-recumbent or recumbent position after 5 minutes of rest and may be repeated at the discretion of the investigator. Daily ECGs must be obtained at the same time each day (+/- 30 min).

Table 6.1-3. Schedule of Events for MAD Phase (Part 3)

Part 3: MAD

Day / Assessments	Screen	Check-in		Dosing								Check-out					Com-pletion
**Assessments**	**-28 to -3**	**-2**	**-1**	**1**	**2**	**3**	**4**	**5**	**6**	**7**	**8**	**9**	**10**	**11**	**12**	**17**	**21 (±1)**
Obtain informed consent before study procedures	X																
Confined to clinic		X	X	X	X	X	X	X	X	X	X	X					
Outpatient visits	X												X	X	X	X	X
Demographics	X																
Height, Weight, BMI	X																
Drug and Alcohol Screen	X	X															
Ultrasound scan (abdominal)	X																
Medical History	X	X (interim)															
Physical Exam [1]	X	X										X					X
Hepatitis and HIV screen (HAVIgM, HBsAg, HBsAB, HBcAb, HCVAb, HIVAb)	X																
Alpha-fetoprotein	X																
HCV genotype	X																
IL28B determination		X															
Vital Signs [2]	X	X	X	X	X	X	X	X	X	X	X	X	X	X	X	X	X
**Randomization [3]**				**X**													
**Study drug administration [4]**				**X**	**X**	**X**	**X**	**X**	**X**	**X**							
AE Evaluation	X--X																
PK Samples [5]				X	X	X	X	X	X	X	X	X	X	X	X		X
Liver function tests	X	X	X	X	X	X	X	X	X	X	X	X	X	X	X	X	X
CBC w/diff., PT/PTT & INR, serum chemistry (other than liver function tests) and Urinalysis[12]	X	X			X					X		X			X		X
HCV RNA concentrations [6,7]	X	X		X	X	X	X	X	X	X	X	X	X	X	X	X	X

Part 3: MAD																	
Day	Screen	Check-in		Dosing								Check-out					Com-pletion
Assessments	-28 to -3	-2	-1	1	2	3	4	5	6	7	8	9	10	11	12	17	21 (±1)
Plasma for drug resistance monitoring[8]		X		X	X	X	X	X	X	X	X	X	X	X	X	X	X
Pregnancy Test [9]	X	X										X					X
12-lead Holter monitoring [10]			X							X							
12-Lead electrocardiogram [11]	X	X		X	X	X	X	X	X	X	X					X	X

1. A complete physical exam (PE) is to be done at screening and at study completion. Symptom directed PE may be conducted as indicated at subsequent visits.
2. Vital signs (BP, HR, RR, body temperature) will be obtained in a semi-recumbent or recumbent position after 5 minutes of rest at Screening, Check-in (Day -2), Day -1, predose and at 1, 2, 4, 6 and 10 hours postdose on Day 1; predose and at 1 and 4 hours postdose on Days 2 through 7; Day 8; prior to clinic check-out (Day 9); and on Days 10, 11, 12, 17 and 21. Vital signs will be conducted after ECG assessments and prior to PK collections.
3. Randomization may take place up to 2 days before first dose.
4. Prior to each dose of study medication, subjects will receive a standard meal and 250 mL of water. Except as part of dosing water may be consumed freely until 1 hour prior & 2 hours after dosing. Subjects may be dosed in a staggered fashion to accommodate postdosing assessments. Dosing should occur at the same time each day +/- 5 minutes (e.g., subject 1 is dosed at 7:00AM on MAD Days 1 through 7, subject 2 is dosed at 7:05 AM on MAD Days 1-7, etc.
5. PK sample times:
   Day 1: predose administration and 0.25, 0.5, 1, 2, 3, 4, 6, 8, 12 hours postdose
   Days 2–6: predose
   Day 7: predose administration and 0.25, 0.5, 1, 2, 3, 4, 6, 8, 12, 24 and 36 (Day 8), 48 (Day 9), 72 (Day 10), 96 (Day 11), 120 (Day 12) postdosing and on Day 21.
   No urine samples for PK analysis will be collected.
6. Measured using COBAS® TaqMan® HCV Test (Version 2.0, Roche).
7. Timing for HCV RNA concentrations:
   Screening and Day -2,
   Day 1 predose and at 1, 4 and 12 hours after 1st dose
   3 hours postdose on Days 2–8
   Day 9 prior to checkout
   At outpatient visits on Days 10, 11, 12, 17 and 21.
8. Plasma for resistance monitoring will be obtained predose during the dosing period and on the same days as samples for HCV RNA evaluation thereafter. Sequencing of the HCV polymerase NS5b coding region will be conducted at baseline, end of treatment and at Day 21 for all subjects enrolled in the study.

9. Pregnancy testing can utilize urine or serum consistent with local standards.

10. 12-lead Holter monitoring will be conducted for 24 hours (+/- 1 hour) prior to dosing, and for 24 hours postdosing after the last dose of study medication

11. 12-lead ECGs will be obtained at Screening, at Check-in; pre-dosing, 1.25, 3.5, 7, and 10 hours on Day 1; and once daily on Days 2 to 8, Day 17, and Day 21. Daily ECGs must be obtained at the same time each day (+/- 30 min). ECGs may be repeated at the discretion of the investigator to account for erroneous readings and will be obtained in a semi-recumbent or recumbent position after 5 minutes of rest

12. Blood for routine safety evaluations should be taken in a fasted state.

Table 6.1-4. Schedule of Events for Resistance Monitoring

Part 3: MAD HCV NS5b Resistance Monitoring			
	**Baseline**	**End of Treatment**	**Completion**
**Assessments** / **Day**	**1**	**7**	**21 (±1)**
Resistance Testing	X	X	X

Sequencing of the HCV polymerase NS5b coding region will be conducted at baseline, end of treatment (Day 7; where HCV RNA levels > 1000 IU/mL) and at Day 21 for subjects with CHC enrolled in the study. Additional sequencing will be conducted in the following circumstances:

a. HCV RNA rebound: defined as subjects who demonstrate a sustained ≥ 1.0 $\log_{10}$ IU/mL increase of HCV RNA above nadir before the end of treatment (≥ 2 consecutive measurements), where nadir is a ≥ 1.0 $\log_{10}$ IU/mL decrease from baseline.

b. Partial response: defined as those subjects who show an initial HCV RNA decline (≥ 1.0 $\log_{10}$ IU/ml decrease from baseline) followed by stabilization (≥ 2 consecutive measurements with HCV RNA levels similar to those at nadir) while receiving AL-335 .

If a patient meets either of these definitions, then attempts will be made to determine the NS5B coding sequences for any samples with HCV RNA > 1000 IU/mL. Subsequently, phenotypic characterization of selected samples may be carried out by the amplification of the entire NS5b coding region and cloning into a replicon shuttle vector.

6.2 ON-STUDY EVALUATIONS, PROCEDURES, AND DOSING

Where multiple procedures are scheduled at the same time point(s) relative to dosing, the following chronology of events should be adhered to, where possible:

- Vital signs: obtain after ECGs and as close as possible to scheduled time, but prior to blood specimen collection.
- Blood pressure/pulse rate: obtain as close as possible to scheduled time, but prior to blood specimen collection.
- PK blood specimens: obtain at scheduled time.
- PK urine samples: subjects may void their bladder up to 10 minutes prior to dosing i.e., 0 hour.
- Assessments conducted within 10% of the nominal time (e.g., within 6 minutes of a 60 minute time point) from dosing will not be captured as a protocol deviation as long as the exact time of the assessment is noted on the source document and data collection record (e.g., CRF).
- Other Procedures: all other procedures should be obtained as close as possible to the scheduled time but may be obtained before or after blood specimen collection
- Where no scheduled time is specified a given evaluation can be conducted 2–4 hours postdosing.

6.2.1 Diet, Fluid, and Activity

While in the Phase 1 unit, subjects in Part 1–3 will receive standardized diets at scheduled times that do not conflict with study-related procedures. In Part 1, prior to each dose of study medication, subjects may fast from food for at least 8 hours overnight until 4 hours postdose (SAD Cohorts 1-5). For the Optional SAD Cohorts (6-8), prior to each dose of study medication subjects will receive a standard meal and 250 mL of water. In Part 2, subjects will receive a single dose in a fasted state (Cohort 3 from Part 1) followed by a single dose in a fed state after an 11- 21 day washout. Except as part of dose administration, water may be consumed freely except between 1 hour prior to dosing and 2 hours post-dosing. In Part 3, prior to each dose of study medication, subjects will receive a standard meal and 250 mL of water.

Subjects in Part 2, the food effect study phase, will be given a standardized high-fat content meal within 30 minutes of their second dose; the meal will be completed at least 10 minutes prior to dosing. The meal will consist of the following (CDER Guidance, 2002):

- Two eggs fried in butter
- Two strips of bacon
- Two slices of toast with butter
- Four ounces of hash brown potatoes (fried with butter)

- Eight ounces (240 mL) of whole milk

Subjects will refrain from any strenuous activities from 48 hours prior to check-in through study completion. Consumption of grapefruit or grapefruit products such as juice are prohibited during study participation. Other prohibited foods that either inhibit or induce the CYP450 enzyme class are included in Appendix C.

6.2.2 Pharmacokinetic Blood and Urine Sampling

Blood samples for PK analysis will be collected at the scheduled times via an indwelling catheter and/or direct venipuncture using 4-mL Vacutainer collection tubes containing sodium fluoride/potassium oxalate anticoagulant pre-spiked with dichlorvos to stabilize the AL-335 and metabolites post-sampling. Saline or heparin flushes may be used to maintain viability of indwelling catheters. Additional information may be found in the Pharmacokinetic Sample Processing Manual.

Urine samples will be collected at the designated time points and processed according to the methodology described in the Laboratory Manual.

Samples may be used for future metabolite identification and/or further evaluation of the bioanalytical method. These data will be used for internal exploratory purposes and will not be included in the clinical study report.

Processing and shipping instructions are detailed in the Laboratory Manual. Plasma and urinary concentrations of AL-335, AL-022399 and AL-02227 will be determined by Inventiv Health Clinical Laboratories using a validated bioanalytical method.

All efforts will be made to obtain the PK samples at the exact nominal time relative to dosing. However, samples obtained within 10% of the nominal time (e.g., within 6 minutes of a 60 minute time point) from dosing will not be captured as a protocol deviation as long as the exact time of the sample collection is noted on the source document and data collection record (e.g., CRF).

To maintain the study blind, results of the individual PK analyses will not be shared with the Principal Investigator or Sponsor representatives involved in managing the study. PK data will be provided in a blinded fashion to the PI and Sponsor representatives to assist with dose escalation decisions during the course of the study.

6.2.3 Central Laboratory Evaluations

Local laboratories will be used for all routine screening and study related laboratory evaluations. A central laboratory will be used to conduct IL-28B, and HCV RNA evaluations.

6.2.4 HCV Evaluations

HCV RNA concentrations will be determined using COBAS® TaqMan® HCV Test (Version 2.0, Roche). To maintain the study blind, results of the individual HCV viral load analyses will not be shared with the PI or Sponsor representatives involved in managing the study.

6.2.5 HCV Resistance Monitoring

Blood taken for monitoring of potential viral resistance to AL-335 will be evaluated by the sponsor or designee by population sequencing of the HCV polymerase NS5B coding region and phenotypic susceptibility testing. Clonal sequencing, Next-generation sequencing, and/or sequencing of other regions of the HCV genome may also be performed. For all subjects with CHC, samples for viral resistance testing will be taken at the same time points as those taken for HCV RNA evaluation (see Table 6.1-3). Samples for viral resistance monitoring will be selected based on the results obtained from the HCV RNA determinations. Samples encompassing the NS5B region or other regions of the HCV genome may also be used in further phenotypic studies.

Further details of HCV resistance monitoring procedures can be found in Table 6.1-4.

Approximately 8mL of blood will be collected at each protocol-specified time point using an indwelling venous catheter and/or direct venipuncture into two 4mL (plastic) K2EDTA Vacutainer tubes. Additional processing information may be found in the Laboratory Manual.

6.2.6 12-Lead Electrocardiograms

The investigator will be responsible for evaluating the results and determining if any findings are of clinical significance. ECGs will be obtained in a semi-recumbent or recumbent position after 5 minutes of rest and may be repeated at the investigator discretion to account for erroneous readings.

6.2.7 Vital Signs

Vital signs (oral or body temperature, respiratory rate, and automated supine blood pressure and heart rate) will be obtained in a semi-recumbent or recumbent position after 5 minutes of rest. Blood draws will take precedence over vital signs in the event that the two procedures need to be conducted at the same time. In this case, vital signs should be obtained as close the scheduled time as possible, after ECG assessments and prior to blood collections.

6.2.8 Physical Examinations

Subjects will have complete physical examination at screening and study completion. A brief symptom directed examination will be conducted at all other timepoints as indicated in the Schedule of Events. BMI will be calculated by clinic staff at the Screening visit using the following formula:

$$\text{BMI} = \text{weight in kg} \div (\text{height in meters})^2$$

6.2.9 Contraception

Males will either be sterile or agree to one of the following methods of contraception from first dose of study drug until 90 days following the final dose of study drug:

- Male condom with spermicide
  A sterile sexual partner

Males will advise their female sexual partners to use:

- A non-hormonal IUD with spermicide
- Female condom with spermicide
- Contraceptive sponge with spermicide
- Diaphragm with spermicide
- Cervical cap with spermicide
- Oral, implantable, transdermal, or injectable contraceptives

For all subjects, two forms of contraception should be employed.

7.0 SAFETY MONITORING AND REPORTING

7.1 DEFINITIONS

7.1.1 Pretreatment Events

A pretreatment event is any event that meets the criteria for an AE/SAE and occurs after the subject signs the informed consent form but before receiving the first administration of study drug.

7.1.2 Adverse Events

An AE is any event, side effect, or untoward medical occurrence in a subject enrolled in a clinical trial whether or not it is considered to have a causal relationship to the study drug. An AE can therefore be any unfavorable and unintended sign, symptom, laboratory finding outside of normal range with associated clinical symptoms or suspected latent clinical symptoms in the opinion of the investigator, physical examination finding, or disease temporally associated with the use of the study drug, whether or not the event is considered related to the study drug.

Planned hospital admissions or surgical procedures for an illness or disease that existed before the subject was enrolled in the study are not to be considered AEs unless the condition deteriorated in an unexpected manner during the study (e.g., surgery was performed earlier than planned).

7.1.3 Serious Adverse Events

A Serious Adverse Event (SAE) is any untoward medical occurrence at any dose that:

- Results in death: This includes deaths that appear to be completely unrelated to study medication (e.g., a car accident).
- Is a life-threatening event: An event that places the subject at immediate risk of

death at the time of the event; it does not refer to an event that hypothetically might have caused death if it were more severe.

- Requires inpatient hospitalization or prolonged hospitalization of an existing hospitalization.
- Results in permanent or prolonged (at least 28 days in duration) disability or incapacity.
- Is a congenital anomaly or birth defect in the offspring of a study subject.
- Medically important event: An event that may not be immediately life-threatening, or result in death or hospitalization, or require intervention to prevent one of the outcomes listed above, but is considered medically significant for other reasons. An opportunistic or otherwise unusual infection for the investigator's practice, such as tuberculosis, will be considered medically significant.

The term severe is used to describe the intensity of a specific event (as in mild, moderate, or severe); the event itself, however, may be of minor medical significance (such as severe headache). This is not the same as serious, which is based on outcome of the event, as described above. Seriousness, not intensity, serves as a guide for defining regulatory reporting obligations.

7.2 DOCUMENTING AND REPORTING OF ADVERSE EVENTS (INCLUDING SERIOUS ADVERSE EVENTS)

Adverse Events will be evaluated and documented using the grading scales contained in the Division of AIDS Table for Grading the Severity of Adult and Pediatric AEs (December 2004) and shown in Appendix E.

7.2.1 Documenting and Reporting Pretreatment Events

For enrolled subjects, record all pretreatment AEs that occur after the subject signs the informed consent form but before the first study drug administration on the AE CRF and Clinical Trials SAE Form (if applicable). The AE CRF and SAE form will indicate that the event occurred prior to the first dose of study drug.

7.2.2 Documenting and Reporting Adverse Events

Record all AEs that occur from Day 1 (from start of study medication administration) to the follow-up visit, regardless of the intensity, seriousness, or relationship to study drug, on the AE CRF, for all enrolled subject. Subjects who do not enroll (i.e., screen failures), collect AEs until the time of screen failure.

Grade AEs (serious and nonserious) in accordance with the scale presented below:

- Mild adverse event (minor; no specific medical intervention; asymptomatic laboratory findings only,; marginal clinical relevance); does not interfere with regular activities

- Moderate adverse event (minimal intervention; local intervention; noninvasive intervention [packing, cautery]); moderate level of discomfort and significantly interferes with regular activities
- Severe and undesirable adverse event (significant symptoms requiring hospitalization or invasive intervention; transfusion; elective interventional radiological procedure; therapeutic endoscopy or operation); significant level of discomfort and prevents regular activities
- Life-threatening or disabling adverse event (complicated by acute, life-threatening metabolic or cardiovascular complications such as circulatory failure, hemorrhage, sepsis. Life-threatening physiologic consequences; need for intensive care or emergent invasive procedure; emergent interventional radiological procedure, therapeutic endoscopy or operation).

Once an event has resolved, any recurrence will be reported as a new event with a corresponding grade.

Whenever possible, report AEs as a specific diagnosis or syndrome (e.g., flu syndrome) rather than as individual signs or symptoms. If no specific diagnosis or syndrome is identified, AEs should be reported as separate and individual events.

An AE includes the following:

- Pre-existing event that increases in frequency or intensity.
- Condition detected or diagnosed during the study period, even though it may have been present, in retrospect, prior to the first dose of study drug.
- Laboratory abnormalities outside of normal limits and requiring therapeutic intervention.
- An overdose of the study drug without any signs or symptoms will be considered an AE. A calculated dose that exceeds its correct dose by 10% or more and is administered to the subject will be considered an overdose and documented as an AE.

The following events **will not** be identified as AEs in this study:

- Progression or exacerbation of the subject's underlying disease. However, clinical sequelae that result from disease progression, such as pleural effusion or small bowel obstruction, are reportable as AEs.
- Medical or surgical procedures (e.g., surgery, endoscopy, tooth extraction, etc); however, the condition (the "triggering event") that leads to the procedure may be an AE.
- Pre-existing conditions present or detected prior to the first dose of study drug that do not worsen.

7.2.3 Assigning Attribution of Adverse Events

The investigator **must** attempt to determine the cause of each event. To ensure consistency of AE/SAE causality assessments, investigators should apply the following guideline:

Related:

There is an association between the event and the administration of investigational study drug, a plausible mechanism for the event to be related to the investigational study drug and causes other than the investigational study drug have been ruled out, and/or the event re-appeared on re-exposure to the investigational study drug.

Possibly Related:

There is an association between the event and the administration of the investigational study drug and there is a plausible mechanism for the event to be related to investigational study drug, but there may also be alternative etiology, such as characteristics of the subject's clinical status or underlying disease.

Unlikely Related:

The event is unlikely to be related to the investigational study drug and likely to be related to factors other than investigational study drug.

Not Related:

The event is related to an etiology other than the investigational study drug (the alternative etiology must be documented in the study subject's medical record).

7.2.4 Classifying Action Taken with Study Drug

Classification	Definition
Dose Not Changed	Study Drug dose not changed in response to the AE
Dose Reduced	Study drug dose reduced in response to an AE
Drug Interrupted	Study drug administration interrupted in response to an AE
Drug Withdrawn	Study drug administration permanently discontinued in response to an AE
Not Applicable	Action taken regarding study drug administration does not apply. "Not applicable" should be used in circumstances such as when the investigational treatment had been completed before the adverse event began and no opportunity to decide whether to continue, interrupt or withdraw treatment is possible.

7.2.5 Classifying Adverse Event Outcome

Classification	Definition
Recovered/Resolved	Resolution of an AE with no residual signs or symptoms
Recovered/Resolved with sequelae	Resolution of an AE with residual signs or symptoms
Not Recovered/ Not resolved (continuing)	Either incomplete improvement or no improvement of an AE, such that it remains ongoing
Fatal	Outcome of an AE is death. "Fatal" should be used when death is at least possibly related to the adverse event.
Unknown	Outcome of an AE is not known (e.g., a subject lost to follow up)

7.2.6 Documenting and Reporting Serious Pretreatment Events and Serious Adverse Events

All SAEs that occur after obtaining informed consent through the Follow–up visit, regardless of causality, must be reported by the investigator to Icon Pharmacovigilance. In addition, all SAEs, including those that result in death, that occur after the Completion Visit and that are considered related to study drug(s) must be reported to Icon Pharmacovigilance within 24 hours.

SAEs will be recorded on the Clinical Trials SAE Form using a recognized medical term or diagnosis that accurately reflects the event. SAEs will be assessed by the investigator for severity, relationship to the investigational study drug(s) and possible etiologies. On the Clinical Trials SAE Form, relationship to study drug(s) will be assessed only as related or not related, and severity assessment will not be required. For the purposes of study analysis, if the event has not resolved at the end of the study reporting period, it will be documented as ongoing. For purposes of regulatory safety monitoring, the investigator is required to follow the event to resolution and report to Icon the outcome of the event using the SAE Form.

The investigator is responsible for notifying the Sponsor within 24 hours of identifying an SAE, regardless of the presumed relationship to the investigational study drug. The SAE Form should be completed for new/initial events as well as to report follow-up information on previously reported events. Investigators are asked to report follow up information as soon as it becomes available, to ensure timely reporting to Health Authorities.

The SAE Form should be faxed to Icon Pharmacovigilance using the fax cover sheet provided:

Serious Adverse Event Contact Information
Icon Pharmacovigilance

SAE Email Address [redacted]

[redacted]

UK SAE Fax Number: [redacted]

[redacted]

Alios Biopharma or its designees, as study sponsor, is responsible for reporting suspected, unexpected, serious adverse reactions (SUSARs) involving the study drug(s) to all regulatory authorities, and participating investigators, in accordance with ICH Guidelines, and/or local regulatory requirements, as applicable.

7.2.7 Documenting and Reporting of Pregnancy

Subjects will be counseled to inform the investigator of any pregnancy that occurs during study treatment and for 90 days after the last dose of study drug/s.

If a subject or the female partner of a male subject becomes pregnant while participating in the study, study treatment must be permanently discontinued immediately. The investigator must notify the Sponsor's Medical Monitor and ICON Pharmacovigilance within 1 business day of the sites' knowledge of the subject's (or partner's) pregnancy, by utilizing the Pregnancy Initial Report Form. If confirmed to be on active drug, the subject or partner will be followed until end of pregnancy and the infant will be followed for 1 year after the birth, provided informed consent is obtained. A separate informed consent form will be provided to explain these follow-up activities. Pregnancy itself does not constitute an adverse event.

7.3 FOLLOW-UP OF ADVERSE EVENTS AND SERIOUS ADVERSE EVENTS

Follow all AEs (serious and non-serious) until resolution or otherwise explained, the subject dies, the event stabilizes and is not expected to further resolve, or when alternative therapy is instituted, whichever occurs first. If alternative therapy is instituted, it should be documented. Alios BioPharma may request that the investigator perform or arrange for supplemental measurements or evaluations to further clarify the nature of the event.

7.4 SPONSOR'S REVIEW OF ADVERSE EVENTS AND SERIOUS ADVERSE EVENTS

Alios BioPharma will maintain an ongoing review of all AEs and SAEs. The PK/PD relationship between AL-335 and its metabolites and various safety parameters including ECG changes, vital signs, and relevant laboratory parameters will be evaluated.

7.5 EMERGENCY UNBLINDING FOR PARTS 1 AND 2 (SAD AND FOOD EFFECT)

Randomization codes will be given to the site pharmacist at the start of the study and maintained in a secure location. Treatment assignment will be made available to the PI in the event of a medical emergency or an adverse event that necessitates identification for

the welfare of a subject. Whenever possible, the PI will contact the Sponsor to discuss the need to break the blind prior to proceeding with unblinding. The PI will notify the sponsor as soon as is practical in the event of the study blind being broken and will document the reason.

7.6 EMERGENCY UNBLINDING FOR PART 3 (MAD)

Randomization information will be made available to the pharmacist for the subjects enrolled at their site. The treatment assignments will be made available to the PI in the event of a medical emergency or an adverse event that necessitates identification for the welfare of a subject. Whenever possible, the PI will contact the Sponsor to discuss the need to break the blind prior to proceeding with unblinding. The PI will notify the sponsor as soon as is practical in the event of the study blind being broken and will document the reason.

8.0 STUDY VARIABLES AND MEASUREMENTS

8.1 EFFICACY VARIABLES/MEASUREMENTS

Serum HCV RNA will be measured in subjects with CHC infection. No efficacy variables will be evaluated in the healthy volunteer cohorts.

8.2 SAFETY VARIABLES/MEASUREMENTS

Safety evaluation will include AEs and SAEs, vital signs, laboratory tests (including hematology and serum chemistries), ECG, physical examination, urinalysis, and pregnancy tests.

8.2.1 Adverse Events

Adverse events, including pretreatment events, will be recorded from the time of consent through 21 days after the last dose of study drug. All AEs/SAEs will be coded using the *Medical Dictionary for Regulatory Activities (MedDRA)*.

8.2.2 Clinical Laboratory Measurements

Clinical laboratory variables, including hematology, serum chemistries, urinalysis, and pregnancy testing, will be assessed at screening. Hematology, serum chemistries, and pregnancy testing will be evaluated periodically during the study as indicated in this protocol.

8.2.3 Prior and Concomitant Medications

Use of all medications and supportive therapy from within 21 days of the screening visit through study completion will be recorded. All concomitant medications will be mapped using the World Health Organization (WHO) Drug Dictionary.

8.3 PHARMACOKINETIC MEASUREMENTS

Predose and postdose plasma and urine samples will be collected from subjects in all dosing cohorts at specified times throughout the study for the determination of PK parameters for AL-335 and its metabolites (timepoints specified in Section 6.1, *Study Procedures*, *Schedule of Events*). Plasma and urine concentrations of study medication and metabolites will be assessed by a liquid chromatography tandem mass spectrometry method and used to calculate the values of PK parameters, including:

Ae	Total amount excreted in urine
$AUC_{0\text{-}inf}$	Area under plasma concentration-time curve from hour 0 to infinity
$AUC_{0\text{-}last}$	Area under plasma concentration-time curve from hour 0 to last sample with measurable plasma concentrations
$AUC_{0\text{-}tau}$	Area under plasma concentration-time curve over one dosing interval
C_{last}	Last measurable plasma concentration
C_{max}	Maximum observed concentration
C_{min}	Minimum observed concentration (for repeated doses)
$Cl_{R(0\text{-}t)}$	Renal clearance over 0 to t time period
CL	Systemic clearance
CL/F	The apparent oral clearance
λz	terminal elimination rate constant
$t_{1/2}$	Terminal elimination half-life
t_{last}	Time to last measurable plasma concentration
t_{max}	Time of maximum concentration
Vd	Volume of distribution
V_z/F	The apparent steady-state volume of distribution

8.4 PHARMACODYNAMIC MEASUREMENTS

Blood samples for HCV viral load measurements will be collected for assessment in subjects with CHC as outlined in Table 6.1-3.

8.5 VIRAL RESISTANCE

Blood samples will be collected from subjects with CHC to assess the potential emergence of viral resistance to AL-335.

9.0 STATISTICAL CONSIDERATIONS

9.1 GENERAL CONSIDERATIONS

Continuous data will be summarized by descriptive statistics, including number of subjects, mean, standard deviation, median, and range. Categorical data will be summarized by the number and percentage of subjects. Time-to-event endpoints will be estimated using the Kaplan-Meier method. All analyses will be presented by dose cohort and the combined study population.

Details will be provided in the statistical analysis plan (SAP). The Clinical Study Report (CSR)

may be written in two stages for this study; one for the SAD and Food Effect portions (Parts 1 and 2) and another for the MAD phase (Part 3).

9.2 STUDY ENDPOINTS

9.2.1 Primary Endpoint

Analysis of safety measures including but not limited to tabulation of treatment emergent adverse events, physical examination findings, vital signs, 12-lead ECGs, and clinical lab results (including chemistry, hematology, and urine).

This endpoint may be characterized by analyzing the number of participants with adverse events per type as a measure of safety and tolerability after increasing single oral doses in HV, as well as analyzing the number of participants with adverse events per type as a measure of safety and tolerability after 7 days of oral dosing in subjects with CHC infection.

9.2.2 Secondary Endpoints

PK parameters of AL-335, ALS-022399, ALS-022227 (and other metabolites if applicable) following single dose administration: C_{max}, t_{max}, $t_{1/2}$, CL/F and V_z/F (for AL-335 only), AUC_{0-inf} or AUC_{last}

PK parameters of AL-335, ALS-022399, ALS-022227 (and other metabolites if applicable) following repeat dose administration: C_{max}, t_{max}, $t_{1/2}$, AUC_{last} and AUC_{0-tau}

PK parameters of AL-335, ALS-022399, ALS-022227 (and other metabolites if applicable) following single dose administration in tablet form as compared with suspension: C_{max}, t_{max}, $t_{1/2}$, CL/F and V_z/F (for AL-335 only), AUC_{0-inf} or AUC_{last}

PK parameters of AL-335, ALS-022399, ALS-022227 (and other metabolites if applicable) after a single oral dose in HV in fasted conditions as compared with fed conditions

Concentration in urine and urinary excretion of AL-335, ALS-022399, ALS-022227 (and other metabolites if applicable) after a single oral dose in HV in fasted conditions

HCV RNA viral load change from baseline in subjects with CHC infection

Sequence analysis of the HCV NS5B region in subjects with CHC infection, as appropriate

9.3 DETERMINATION OF SAMPLE SIZE

No formal sample size calculations have been performed as this is an exploratory study. The number of subjects participating in each cohort is considered sufficient to achieve the objectives of the study. Approximately 40 (up to 64) healthy subjects, and 70 (up to 80) subjects with CHC infection will be evaluated in this study.

The number of subjects is considered sufficient to support PK and safety assessments, however as this is a small study no sample size calculation was performed. Three additional doses in the SAD and one additional in the MAD part of the study may be enrolled at the

discretion of the Sponsor and Principal Investigator (PI) based on the emerging PK profile and the presence of an acceptable safety profile.

10.0 RANDOMIZATION

In Part 1, approximately 40 (up to 64) subjects will be assigned to up to 8 single-dose cohorts of 8 subjects each. For each dose cohort, 8 subjects will be randomized in a 3:1 ratio to receive AL-335 or placebo.

For Part 2, subjects in Cohort 3 from Part 1 will receive the same assignment for their second dose.

In Part 3, approximately 70 (up to 80) subjects with CHC infection will be enrolled. For each dose cohort, 10 subjects will be randomized in a 4:1 ratio to receive AL-335 or placebo.

Subjects will not be stratified by any baseline characteristics.

10.1 STUDY CONDUCT

10.1.1 Subject Disposition

An accounting of all subjects over the course of the study will be reported by dose cohort. Enrollment, study drug administration, subject completion, premature discontinuation, and major protocol violations will be tabulated and summarized by dose cohort.

10.1.2 Replacement of Subjects

All 8 HV subjects and at least 8 of the 10 subjects with CHC infection must complete the dosing schedule for each cohort. A decision to replace subjects will be made by the Sponsor in consultation with the PI. Subjects withdrawn for safety reasons will not be replaced.

10.1.3 Procedures for Handling Missing, Unused, or Spurious Data

Strategies for handling missing, unused, or spurious data will be specified in the SAP.

10.2 ANALYSIS DATA SETS

The Full Analysis Set (FA) is defined as all enrolled subjects who have received at least one dose of study medication. Further analysis data sets will be defined for each part of the study in SAP.

10.3 DEMOGRAPHICS AND BASELINE CHARACTERISTICS

Demographic data (age, sex, ethnicity, body weight) and baseline disease characteristics will be tabulated and summarized by dose cohort and presented in data listings.

10.4 SAFETY ANALYSIS

10.4.1 Adverse Events

The MedDRA medical dictionary will be used to map the AE/SAE verbatim terms to specific system organ classes (SOC) and preferred terms. Adverse events and SAEs will be summarized in summary tables and tabulated in by-subject listings by SOC and preferred term for each dosing cohort and by treatment assignment (AL-335 or placebo). Incidence rates will be presented.

10.4.2 Vital Signs, ECG, Physical Examination, and Laboratory Assessments

Vital signs, including resting pulse, blood pressure, and body temperature, will be summarized and tabulated by dosing cohort and by treatment assignment (AL-335 or placebo). The proportion of subjects with abnormal findings based on physical examinations over time will be summarized by dosing cohort and by treatment assignment (AL-335 or placebo). Laboratory values will be graded and summarized based on CTCAE v3.0. Number and percentage of subjects with Grades 3 and 4 laboratory abnormalities will be tabulated by dosing cohort and by treatment assignment (AL-335 or placebo).

In the event a safety signal is detected, the PK/PD relationship between AL-335 and its metabolites and various safety parameters including ECG changes, vital signs, and relevant laboratory parameters will be evaluated.

10.4.3 Concomitant Medications

All reported concomitant medications will be mapped using the WHO Drug Dictionary. Concomitant medications will be tabulated in summary tables and by-subject listings.

10.5 PHARMACOKINETIC ANALYSIS

The calculated values for all PK parameters will be tabulated by subject, by dose cohort and by treatment assignment and presented as graphs and by-subject listings. Summary statistics will be performed by cohort by treatment.

Dose proportionality will be assessed in Parts 1 and 3 using the power model for C_{max}, and AUC. Assessment of the steady state will be performed in Part 3 on C_{trough} and AUC.

Bioequivalence within the food conditions will be assessed in Part 2 on C_{max} and AUC. T_{max} will be compared. Additional details for statistical consideration for PK analysis will be defined in the SAP.

10.6 HCV VIRAL LOAD ANALYSIS

For subjects with CHC infection only, raw data mean changes in HCV concentration as well as maximum decrease from baseline will be tabulated by dose group. Data will also be presented in graphs by treatment assignment, by cohort and by individual. All individual values will be presented in subject listings.

11.0 ADMINISTRATIVE CONSIDERATIONS

The investigator and/or sponsor, consistent with local regulatory practice, will submit this protocol, the informed consent, investigator's brochure, and any other relevant supporting information to the competent authority and appropriate IRB/IEC for review and approval prior to study initiation. A letter confirming IRB/IEC approval of the protocol and informed consent, a statement that the IRB/IEC is organized and operates according to GCP and the applicable laws and regulations, and financial disclosures **must** be forwarded to Alios BioPharma prior to screening subjects for the study. Amendments to the protocol must also be approved by the IRB/IEC and local regulatory agency, as appropriate, prior to the implementation of changes in this study.

11.1 STUDY COMPLIANCE

The study will be conducted in compliance with this protocol, principles of ICH GCP, Declaration of Helsinki, and all applicable national regulations governing clinical trials.

11.2 INFORMED CONSENT AND PROTECTED SUBJECT HEALTH INFORMATION AUTHORIZATION

A copy of the IRB/IEC-approved informed consent must be forwarded to Alios BioPharma for regulatory purposes. The investigator or designee **must** explain to each subject the purpose and nature of the study, the study procedures, the possible adverse effects, and all other elements of consent as defined in § 21CFR Part 50, EU regulations (for EU sites), and other applicable national and local regulations governing informed consent. Each subject must provide a signed and dated informed consent prior to enrollment into this study. Signed consent forms must remain in each subject's study file and be available for verification by study monitors at any time.

In accordance with individual local and national subject privacy regulations, the investigator or designee **must** explain to each subject prior to screening that for the evaluation of study results, the subject's protected health information obtained during the study may be shared with Alios BioPharma and its designees, regulatory agencies, and IECs/IRBs. As the study sponsor, Alios BioPharma will not use the subject's protected health information or disclose it to a third party without applicable subject authorization. It is the investigator's or designee's responsibility to obtain written permission to use protected health information from each subject, or if appropriate, the subject's legal guardian. If a subject or subject's legal guardian withdraws permission to use protected health information, it is the investigator's responsibility to obtain the withdrawal request in writing from the subject or subject's legal guardian **and** to ensure that no further data will be collected from the subject. Any data collected on the subject prior to withdrawal will be used in the analysis of study results.

11.3 SUBJECT SCREENING LOG

The investigator **must** keep a record that lists **all** subjects considered for screening in the study. For those subjects subsequently excluded, record the reason(s) for exclusion.

11.4 CASE REPORT FORMS

Study site personnel will complete CRFs designed for this study according to the completion guidelines that will be provided. Paper CRFs should be completed in black or dark-blue ink. All corrections on paper CRFs will be made by drawing a single line through the information to be corrected, without obscuring it. All corrections will be initialed and dated (and explained, if necessary). Do not use "white-out" or obscuring correction fluid/tape to make changes. An electronic CRF (eCRF) may be used for this study. Study site personnel will be trained and authorized to use the system in compliance with 21CFR Part 11 prior to recording data on eCRFs. All corrections to eCRFs will be made by authorized users, and the changes will be automatically logged in the system.

The investigator will ensure that the CRFs are accurate, complete, legible, and completed in a timely fashion. Separate source records are required to support all CRF entries. The CRF is not to be used to document data without prior written or electronic records. Case report forms should be completed for every subject enrolled in the study. At the study's conclusion, a PDF file will be created for each site containing their subjects' data submitted on eCRFs. In the event of an audit or regulatory authority inspection, copies of the eCRFs will be printed.

11.5 STUDY MONITORING REQUIREMENTS

Representatives of Alios BioPharma or its designee will monitor this study until completion. Monitoring will be conducted through personal visits with the investigator and site staff as well as any appropriate communications by mail, fax, e-mail, or telephone. The purpose of monitoring is to ensure compliance with the protocol and the quality and integrity of the data. This study is also subject to Quality Assurance reviews and/or audits under the Alios BioPharma Clinical Quality Assurance program.

Every effort will be made to maintain the anonymity and confidentiality of all subjects during this clinical study. However, because of the experimental nature of this treatment, the investigator agrees to allow the IRB/IEC, representatives of Alios BioPharma, its designated agent, and authorized employees of the appropriate regulatory agencies to inspect the facilities used in this study and, for purposes of verification, allow direct access to the hospital or clinic records of all subjects enrolled into this study. A statement to this effect will be included in the informed consent form authorizing the use of protected health information.

11.6 RETENTION OF RECORDS

The investigator must retain a copy of all documents relating to this clinical trial for a minimum of 5 years after a marketing application is approved for the drug, unless

Alios BioPharma notifies the investigator in writing that the documents no longer need to be retained because a marketing application will not be filed. The investigator must retain the documents for a longer period, where so required by other applicable requirements. Essential documents shall be archived in a way that ensures that they are readily available, upon request, to the competent authorities and appropriate regulatory authorities. The medical files of trial subjects shall be retained in accordance with national legislation and the maximum period of time permitted by the hospital, institution, or private practice. The investigator is responsible for contacting Alios BioPharma before any study-related documents are moved to another location or destroyed, and he or she must receive written approval from Alios BioPharma before such relocation or destruction of documents proceeds.

11.7 CONFIDENTIALITY AND PUBLICATION POLICY

By conducting this study, the investigator affirms to Alios BioPharma that all study results and information furnished by Alios BioPharma will be maintained in strict confidence. Such information will be communicated to the investigator's IRB/IEC under an appropriate understanding of confidentiality.

A published summary of the results of this study is, however, permissible according to Alios BioPharma and is not inconsistent with the preceding affirmation of confidentiality. Any publication of data collected as a result of this study will be considered a joint publication by the investigator and appropriate Alios BioPharma personnel. Authorship, including order, will be determined by Alios BioPharma in consultation with the Principal Investigator. Contribution of the author to the study design, enrollment, data review, and manuscript preparation and review will be considered when determining the order of authorship for multicenter studies. Alios BioPharma must receive a copy of any presentation, manuscript, or abstract for review at least 45 days prior to public presentation or submission for publication. Any publication outside of this agreement is not permitted.

11.8 CONDUCT OF STUDY AND PROTECTION OF HUMAN SUBJECTS

The principal investigator must ensure the following (unless sponsor is required per local regulations):

1. He or she will personally conduct or supervise the study.
2. His or her staff and all persons who assist in the conduct of the study clearly understand their responsibilities and have their names included in the Study Staff Signature and Delegation of Authority log. The investigator will sign the authorization log whenever it is updated with new responsibilities or staff membership.
3. The study is conducted according to the protocol and all applicable regulations.
4. The protection of each subject's rights and welfare is maintained.
5. Signed and dated informed consent and permission to use protected health information are obtained from each subject prior to conducting study procedures. If a subject or

subject's legal guardian withdraws permission to use protected health information, the investigator will obtain a written request from the subject or subject's legal guardian and will ensure that no further data be collected from the subject.

6. The consent process is conducted in compliance with all applicable regulations and privacy acts.
7. The IRB/IEC and local competent authority comply with applicable regulations and conducts initial and ongoing reviews and approvals of the study.
8. Any amendment to the protocol is submitted promptly to the competent authority and IRB/IEC.
9. Any significant protocol deviations are reported to Alios BioPharma, the local competent authority and the IRB/IEC according to the guidelines at each study site.
10. All Safety Reports are submitted promptly to the local competent authority and IRB/IEC in accordance with the institution's internal policy.
11. All SAEs are reported to Icon Pharmacovigilance within 24 hours of knowledge of the event, and to the local competent authority and the IRB/IEC.

12.0 REFERENCES

[AASLD] American Association for the Study of Liver Diseases and the Infectious Diseases Society of America. Recommendations for Testing, Managing, and Treating Hepatitis C. Accessed from http://www.hcvguidelines.org/full-report-view on 9/17/2014.

[CDER] Center for Drug Evaluation and Research (CDER), US Food and Drug Administration Guidance for industry: Estimating the Maximum Safe Starting Dose in Initial Clinical Trials for Therapeutics in Adult Healthy Volunteers. July 2005.

[CDER]Center for Drug Evaluation and Research (CDER), US Food and Drug Administration Guidance for industry: Food-effect bioavailability and fed bioequivalence studies. December 2002.

Fontana RJ. Side effects of long term oral antiviral therapy for hepatitis B. Hepatology 2009;49(5):S185–195.

Lawitz E, Mangia A, Wyles D. Sofosbuvir for previously untreated chronic hepatitis C infection. N Engl J Med 2013;368:1878–87.

Lewis W and Dalakas MC. Mitochondrial toxicity of antiviral drugs. Nature Medicine 1995;1:417–22.

Perz JF, Armstrong GL, Farrington LA, et al. The contribution of hepatitis B virus and hepatitis C virus infections to cirrhosis and primary liver cancer worldwide. J Hepatol 2006 Oct;45(4):529–38.

Planas R, Balleste B, Alvarez MA, et al. Natural history of decompensated hepatitis C virus-related cirrhosis. A study of 200 patients. J Hepatol 2004;40:823–30.

Poynard T, Bedossa P, Opolon P. Natural history of liver fibrosis progression in patients with chronic hepatitis C. The OBSVIRC, METAVIR, CLINIVIR, and DOSVIRC groups. Lancet 1997;349:825–32.

Simmonds P, Bukh J, Combet C, et al. Consensus proposals for a unified system of nomenclature of hepatitis C virus genotypes. Hepatology 2005;42:962–73.

Svarovskaia E, Dvory-Sobol H, Parkin N, et al. Infrequent development of resistance in genotype 1-6 hepatitis C virus-infected subjects treated with sofosbuvir in Phase 2 and 3 clinical trials. Clin Infect Dis 2014;59(12):1666-74.

World Health Organization website Global Alert and Response. http://www.who.int/csr/disease/hepatitis/en/index.html referenced on 3/14/11.

13.0 APPENDICES

Appendix A. Investigator Signature Page 67
Appendix B. Clinical Laboratory Evaluations 68
Appendix C. Prohibited Medications 70
Appendix D. Blood Volumes 71
Appendix E. Toxicity Tables 74
Appendix F. Child-Pugh Score 106

Appendix B. Clinical Laboratory Evaluations

**Chemistries:**	**Hematology (CBC):**
Albumin<br>Bicarbonate<br>BUN<br>Calcium<br>Chloride<br>Cholesterol<br>Creatinine<br>Creatine Kinase<br>Glucose<br>INR<br>Lipase<br>LDH<br>Phosphorus<br>Potassium<br>PT/PTT<br>Sodium<br>Total protein<br>Triglycerides<br>Uric acid<br>Liver function tests:<br>Alkaline phosphatase<br>ALT<br>AST<br><br>Total bilirubin<br>Direct bilirubin	Hematocrit<br>Hemoglobin<br>MCH<br>MCHC<br>MCV<br>MPV<br>Platelet count<br>RDW<br>Red blood cell count<br>White blood cell count<br>White blood cell differentiation<br>(Percentage and ABS)<br><br>Basophils<br>Eosinophils<br>Lymphocytes<br>Monocytes<br>Neutrophils
**Urinalysis (UA):**<br><br>Color and appearance<br>pH and SG<br>Bilirubin<br>Glucose<br>Ketones<br>Leukocytes<br>Nitrite<br>Occult blood/hematuria<br>Protein<br>Urobilinogen<br>Microscopic (inc RBSs and WBCs)- reflex test only	**In-house Drug Screen:**<br><br>*Based on Phase 1 unit*

Females only:	Other Tests:
Pregnancy Test<br>FSH and estradiol (consistent with institutional standards)	HAV IgM<br>HBsAg<br>HBsAb<br>HBcAb<br>HCVAb<br>HIVAb<br>HCV RNA<br>Alpha fetoprotein (AFP)

Appendix C. Prohibited Medications

Drugs that May Affect AL-335	
**CYP3A Inducers**	**Strong Inhibitors of CYP3A**
Carbamazepine	Itraconazole, lopinavir/ritonavir
Phenytoin	clarithromycin
Rifampin	ritonavir
St. John's wort	ketoconazole
	indinavir/ritonavir
	conivaptan
**Drugs that Might be Affected by AL-335 (Sensitive or narrow therapeutic CYP3A substrates)**	
Alfentanil	Fluticasone (inhaled prohibited; nasal is ok)
Alfuzosin	Lovastatin
Amiodarone	Lurasidone
Aprepitant	Midazolam (oral)
Bepridil	Nisoldipine
Budesonide	Pimozide
Buspirone	Propafenone
Cisapride	Quetiapine
Conivaptan	Quinidine
Darifenacin	Salmeterol
Dasatinib	Sildenafil
Domperidone	Simvastatin
Dronedarone	Tadalafil
Eletriptan	Ticagrelor
Eplerenone	Tolvaptan
Ergot derivatives (dihydroergotamine, ergotamine, ergonovine, methylergonovine)	Triazolam
Felodipine	Vardenafil
Flecainide	
**Drugs that May be Affected by AL-335 (Use with caution)**	
Alprazolam	Methadone
Amlodipine	Midazolam (IV)
Atorvastatin	Nicardipine
Bosentan	Nifedipine
Colchicine	Repaglinide
Diltiazem	Trazodone
Fentanyl	Warfarin
Lidocaine (local use is ok)	Zolpidem

Appendix D. Blood Volumes

Table 1: Estimated Blood Volumes: SAD

Days	Screen	Check-in				Check-out		Completion
**Assessments**	**-21 to -3**	**-2**	**-1**	**1**	**2**	**3**	**4 thru 6 (each day)**	**8**
Drug Screen	2	2						
PK Samples				40	8	4	4	4
Serum Chemistries w/ALT and AST	5	5			5	5		5
CBC w/ INR, PT/PTT	3	3			3	3		3
Hepatitis and HIV screen	4							
Pregnancy Test	2	2						2
Totals	16	12		40	16	12	12	14

Estimated total volume is ~ 122 mL. Note that volumes are approximate. There may be some variation in the volumes that are required between laboratories at different clinical trial centers.

Part 2: Food Effect

Days / Assessments	Screen	Check-in			Checkout					Check-in			Checkout				
	**-21 to -3**	**-1**	**1**	**2**	**3**	**4**	**5**	**6**	**8**	**11-21**	**+1**	**+2**	**+3**	**+4**	**+5**	**+6**	**+8**
Hepatitis and HIV screen	4																
PK Samples			40	8	4	4	4	4	4		40	8	4	4	4	4	4
Serum Chemistries w/ ALT & AST	5	5		5	5				5	5		5	5				
Drug Screen	2	2								2							
CBC w/diff., PT/PTT & INR and Urinalysis	3	3		3	3				3	3		3	3				3
Pregnancy Test	2	2								2							
Totals	16	12	40	16	12	4	4	4	12	12	40	16	12	4	4	4	7

Estimated total volume is ~ 219 mL. Note that volumes are approximate. There may be some variation in the volumes that are required between laboratories at different clinical trial centers.

Part 3: MAD																	
	Screen	Check-in		Dosing								Check-out					Completion
**Assessments** / **Day**	**-28 to -2**	**-2**	**-1**	**1**	**2**	**3**	**4**	**5**	**6**	**7**	**8**	**9**	**10**	**11**	**12**	**17**	**21 (±1)**
Drug Screen	2	2															
Hepatitis and HIV screen (HBsAg, HBsAB, HBcAb, HCVAb, HIVAb)	10																
HCV genotype	3																
IL28B determination		3															
PK Samples				40	4	4	4	4	4	40	8	4	4	4	4	4	4
Liver Function Tests		2	2	2	2	2	2	2	2	2	2	2	2	2	2	2	2
CBC w/diff PT/PTT & INR and Urinalysis, Serum Chemistry	15	15			15					15		15			15		15
HCV RNA concentration	6	6		24	6	6	6	6	6	6	6	6	6	6	6	6	6
Plasma for drug resistance monitoring		8		8	8	8	8	8	8	8	8	8	8	8	8	8	8
Pregnancy Test	2	2										2					2
Alpha-fetoprotein	7																
Totals	43	38	2	74	35	20	20	20	20	71	24	37	20	20	35	20	37

Estimated total volume is ~ 536 mL. Note that volumes are approximate. There may be some variation in the volumes that are required between laboratories at different clinical trial centers.

Appendix E. Toxicity Tables

DIVISION OF AIDS TABLE FOR GRADING THE SEVERITY OF ADULT AND PEDIATRIC ADVERSE EVENTS. PUBLISH DATE: DECEMBER, 2004

Quick Reference

The Division of AIDS Table for Grading the Severity of Adult and Pediatric AEs (“DAIDS grading table”) is a descriptive terminology to be utilized for AE reporting in this study. A grading (severity) scale is provided for each AE term.

General Instructions

Estimating Severity Grade
If the need arises to grade a clinical AE that is not identified in the DAIDS grading table, use the category “Estimating Severity Grade” located at the top of the table on the following page.

Grading Adult and Pediatric AEs
The DAIDS grading table includes parameters for grading both adult and pediatric AEs. When a single set of parameters is not appropriate for grading specific types of AEs for both adult and pediatric populations, separate sets of parameters for adult and/or pediatric populations (with specified respective age ranges) are provided. If there is no distinction in the table between adult and pediatric values for a type of AE, then the single set of parameters listed is to be used for grading the severity of both adult and pediatric events of that type.

Determining Severity Grade
If the severity of an AE could fall under either one of 2 grades (e.g., the severity of an AE could be either grade 2 or grade 3), select the higher of the 2 grades for the AE.

Note: The laboratory normal ranges should be taken into consideration to assign gradings to a laboratory value.

Definitions

Basic self-care functions	Adult: activities such as bathing, dressing, toileting, transfer/movement, continence, and feeding.<br>Young children: activities that are age and culturally appropriate (e.g., feeding self with culturally appropriate eating implement).
Usual social & functional activities	Adult: adaptive tasks and desirable activities, such as going to work, shopping, cooking, use of transportation, pursuing a hobby, etc.<br>Young Children: activities that are age and culturally appropriate (e.g., social interactions, play activities, learning tasks, etc.).
Medical intervention	Use of pharmacologic or biologic agent(s) for treatment of an AE.
Operative intervention	Surgical OR other invasive mechanical procedures.

PARAMETER	GRADE 1<br>MILD	GRADE 2<br>MODERATE	GRADE 3<br>SEVERE	GRADE 4<br>POTENTIALLY<br>LIFE-THREATENING
**ESTIMATING SEVERITY GRADE**				
Clinical AE NOT identified elsewhere in this DAIDS grading table	Symptoms causing no or minimal interference with usual social & functional activities	Symptoms causing greater than minimal interference with usual social & functional activities	Symptoms causing inability to perform usual social & functional activities	Symptoms causing inability to perform basic self-care functions OR Medical or operative intervention indicated to prevent permanent impairment, persistent disability, or death
**SYSTEMIC**				
Acute systemic allergic reaction	Localized urticaria (wheals) with no medical intervention indicated	Localized urticaria with medical intervention indicated OR Mild angioedema with no medical intervention indicated	Generalized urticaria OR Angioedema with medical intervention indicated OR Symptomatic mild bronchospasm	Acute anaphylaxis OR Life-threatening bronchospasm OR laryngeal edema
Chills	Symptoms causing no or minimal interference with usual social & functional activities	Symptoms causing greater than minimal interference with usual social & functional activities	Symptoms causing inability to perform usual social & functional activities	NA
Fatigue<br>Malaise	Symptoms causing no or minimal interference with usual social & functional activities	Symptoms causing greater than minimal interference with usual social & functional activities	Symptoms causing inability to perform usual social & functional activities	Incapacitating fatigue/ malaise symptoms causing inability to perform basic self-care functions
Fever (nonaxillary)	37.7°C – 38.6°C	38.7°C – 39.3°C	39.4°C – 40.5°C	> 40.5°C

PARAMETER	GRADE 1 MILD	GRADE 2 MODERATE	GRADE 3 SEVERE	GRADE 4 POTENTIALLY LIFE-THREATENING
Pain (indicate body site)<br>DO NOT use for pain due to injection<br>(See Injection site reactions: Injection site pain)<br>See also Headache, Arthralgia, and Myalgia	Pain causing no or minimal interference with usual social & functional activities	Pain causing greater than minimal interference with usual social & functional activities	Pain causing inability to perform usual social & functional activities	Disabling pain causing inability to perform basic self-care functions OR Hospitalization (other than emergency room visit) indicated

Basic Self-care Functions – Adult: Activities such as bathing, dressing, toileting, transfer/movement, continence, and feeding.
Basic Self-care Functions – Young Children: Activities that are age and culturally appropriate (e.g., feeding self with culturally appropriate eating implement).
Usual Social & Functional Activities – Adult: Adaptive tasks and desirable activities, such as going to work, shopping, cooking, use of transportation, pursuing a hobby, etc.

Usual Social & Functional Activities – Young Children: Activities that are age and culturally appropriate (e.g., social interactions, play activities, learning tasks, etc.).

CLINICAL				
**PARAMETER**	**GRADE 1 MILD**	**GRADE 2 MODERATE**	**GRADE 3 SEVERE**	**GRADE 4 POTENTIALLY LIFE-THREATENING**
Unintentional weight loss	NA	5% – 9% loss in body weight from baseline	10% – 19% loss in body weight from baseline	≥ 20% loss in body weight from baseline OR Aggressive intervention indicated [e.g., tube feeding or total parenteral nutrition (TPN)]
**INFECTION**				
Infection (any other than HIV infection)	Localized, no systemic antimicrobial treatment indicated AND Symptoms causing no or minimal interference with usual social & functional activities	Systemic antimicrobial treatment indicated OR Symptoms causing greater than minimal interference with usual social & functional activities	Systemic antimicrobial treatment indicated AND Symptoms causing inability to perform usual social & functional activities OR Operative intervention (other than simple incision and drainage) indicated	Life-threatening consequences (e.g., septic shock)
**INJECTION SITE REACTIONS**				
Injection site pain (pain without touching) Or Tenderness (pain when area is touched)	Pain/tenderness causing no or minimal limitation of use of limb	Pain/tenderness limiting use of limb OR Pain/tenderness causing greater than minimal interference with usual social & functional activities	Pain/tenderness causing inability to perform usual social & functional activities	Pain/tenderness causing inability to perform basic self-care function OR Hospitalization (other than emergency room visit) indicated for management of pain/tenderness
Injection site reaction (localized)				
**Adult > 15 years**	Erythema OR Induration of 5 x 5 cm – 9 x 9 cm (or 25 cm^2 – 81cm^2)	Erythema OR Induration OR Edema > 9 cm any diameter (or > 81 cm^2)	Ulceration OR Secondary infection OR Phlebitis OR Sterile abscess OR Drainage	Necrosis (involving dermis and deeper tissue)

CLINICAL				
PARAMETER	GRADE 1<br>MILD	GRADE 2<br>MODERATE	GRADE 3<br>SEVERE	GRADE 4<br>POTENTIALLY<br>LIFE-THREATENING
**Pediatric ≤ 15 years**	Erythema OR Induration OR Edema present but ≤ 2.5 cm diameter	Erythema OR Induration OR Edema > 2.5 cm diameter but < 50% surface area of the extremity segment (e.g., upper arm/thigh)	Erythema OR Induration OR Edema involving ≥ 50% surface area of the extremity segment (e.g., upper arm/thigh) OR Ulceration OR Secondary infection OR Phlebitis OR Sterile abscess OR Drainage	Necrosis (involving dermis and deeper tissue)

Basic Self-care Functions – Adult: Activities such as bathing, dressing, toileting, transfer/movement, continence, and feeding.

Basic Self-care Functions – Young Children: Activities that are age and culturally appropriate (e.g., feeding self with culturally appropriate eating implement).

Usual Social & Functional Activities – Adult: Adaptive tasks and desirable activities, such as going to work, shopping, cooking, use of transportation, pursuing a hobby, etc.

Usual Social & Functional Activities – Young Children: Activities that are age and culturally appropriate (e.g., social interactions, play activities, learning tasks, etc.).

CLINICAL				
**PARAMETER**	**GRADE 1 MILD**	**GRADE 2 MODERATE**	**GRADE 3 SEVERE**	**GRADE 4 POTENTIALLY LIFE-THREATENING**
Pruritis associated with injection See also Skin: Pruritis (itching - no skin lesions)	Itching localized to injection site AND Relieved spontaneously or with < 48 hours treatment	Itching beyond the injection site but not generalized OR Itching localized to injection site requiring ≥ 48 hours treatment	Generalized itching causing inability to perform usual social & functional activities	NA
**SKIN – DERMATOLOGICAL**				
Alopecia	Thinning detectable by study participant (or by caregiver for young children and disabled adults)	Thinning or patchy hair loss detectable by health care provider	Complete hair loss	NA
Cutaneous reaction/rash	Localized macular rash	Diffuse macular, maculopapular, or morbilliform rash OR Target lesions	Diffuse macular, maculopapular, or morbilliform rash with vesicles or limited number of bullae OR Cutaneous reaction /rash with superficial ulcerations of mucous membrane limited to 1 site[a] OR Cutaneous reaction/rash with at least 1 of the following[a]: elevation of AST and/or ALT > 2 x baseline but at least 5 x ULN[a]; fever (> 38°C or 100°F) [a]; eosinophils > 1000/mm^{3}[a]; serum sickness-like reaction[a]	Extensive or generalized bullous lesions OR Stevens-Johnson syndrome (SJS) OR Ulceration of mucous membrane involving 2 or more distinct mucosal sites OR Toxic epidermal necrolysis (TEN)
Hyperpigmentation	Slight or localized	Marked or generalized	NA	NA
Hypopigmentation	Slight or localized	Marked or generalized	NA	NA

CLINICAL				
**PARAMETER**	**GRADE 1 MILD**	**GRADE 2 MODERATE**	**GRADE 3 SEVERE**	**GRADE 4 POTENTIALLY LIFE-THREATENING**
Pruritis (itching – no skin lesions) (See also Injection site reactions: Pruritis associated with injection)	Itching causing no or minimal interference with usual social & functional activities	Itching causing greater than minimal interference with usual social & functional activities	Itching causing inability to perform usual social & functional activities	NA
**CARDIOVASCULAR**				
Cardiac arrhythmia (general) (By ECG or physical exam)	Asymptomatic AND No intervention indicated	Asymptomatic AND Nonurgent medical intervention indicated	Symptomatic, non-life threatening AND Nonurgent medical intervention indicated	Life-threatening arrhythmia OR Urgent intervention indicated

Basic Self-care Functions – Adult: Activities such as bathing, dressing, toileting, transfer/movement, continence, and feeding.
Basic Self-care Functions – Young Children: Activities that are age and culturally appropriate (e.g., feeding self with culturally appropriate eating implement).
Usual Social & Functional Activities – Adult: Adaptive tasks and desirable activities, such as going to work, shopping, cooking, use of transportation, pursuing a hobby, etc.
Usual Social & Functional Activities – Young Children: Activities that are age and culturally appropriate (e.g., social interactions, play activities, learning tasks, etc.).

CLINICAL				
**PARAMETER**	**GRADE 1 MILD**	**GRADE 2 MODERATE**	**GRADE 3 SEVERE**	**GRADE 4 POTENTIALLY LIFE-THREATENING**
Cardiac ischemia/ infarction	NA	NA	Symptomatic ischemia (stable angina) OR Testing consistent with ischemia	Unstable angina OR Acute myocardial Infarction
Hemorrhage (significant acute blood loss)	NA	Symptomatic AND No transfusion indicated	Symptomatic AND Transfusion of ≤ 2 units packed RBCs (for children ≤ 10 cc/kg) indicated	Life-threatening hypotension OR Transfusion of > 2 units packed RBCs (for children > 10 cc/kg) indicated
Hypertension[a]				
**Adult > 17 years** (with repeat testing at same visit)	> 140 to < 160 mmHg systolic OR > 90 to < 100 mmHg diastolic	≥ 160 to < 180 mmHg systolic OR ≥ 100 to < 110 mmHg diastolic	≥ 180 mmHg systolic OR ≥ 110 mmHg diastolic	Life-threatening consequences (e.g., malignant hypertension) OR Hospitalization indicated (other than emergency room visit)
**Pediatric ≤ 17 years** (with repeat testing at same visit)	NA	91st – 94th percentile adjusted for age, height, and gender (systolic and/or diastolic)	≥ 95th percentile adjusted for age, height, and gender (systolic and/or diastolic)	Life-threatening consequences (e.g., malignant hypertension) OR Hospitalization indicated (other than emergency room visit)
Hypotension	NA	Symptomatic, corrected with oral fluid replacement	Symptomatic, i.v. fluids indicated	Shock requiring use of vasopressors or mechanical assistance to maintain blood pressure
Pericardial effusion	Asymptomatic, small effusion requiring no intervention	Asymptomatic, moderate or larger effusion requiring no intervention	Effusion with non-life threatening physiologic consequences OR Effusion with nonurgent intervention indicated	Life-threatening consequences (e.g., tamponade) OR Urgent intervention indicated
Prolonged PR interval				
**Adult > 16 years**	PR interval 0.21 – 0.25 s	PR interval > 0.25 s	Type II 2nd degree AV block OR Ventricular pause > 3.0 s	Complete AV block

CLINICAL					
PARAMETER		GRADE 1 MILD	GRADE 2 MODERATE	GRADE 3 SEVERE	GRADE 4 POTENTIALLY LIFE-THREATENING
	**Pediatric ≤ 16 years**	1st degree AV block (PR > normal for age and rate)	Type I 2nd degree AV block	Type II 2nd degree AV block	Complete AV block

[a] Revised by Tibotec.

Basic Self-care Functions – Adult: Activities such as bathing, dressing, toileting, transfer/movement, continence, and feeding.
Basic Self-care Functions – Young Children: Activities that are age and culturally appropriate (e.g., feeding self with culturally appropriate eating implement).
Usual Social & Functional Activities – Adult: Adaptive tasks and desirable activities, such as going to work, shopping, cooking, use of transportation, pursuing a hobby, etc.
Usual Social & Functional Activities – Young Children: Activities that are age and culturally appropriate (e.g., social interactions, play activities, learning tasks, etc.).

CLINICAL					
PARAMETER		GRADE 1<br>MILD	GRADE 2<br>MODERATE	GRADE 3<br>SEVERE	GRADE 4<br>POTENTIALLY<br>LIFE-THREATENING
Prolonged QTc					
	**Adult > 16 years**	Asymptomatic, QTc interval 0.45 – 0.47 s OR Increase in interval < 0.03 s above baseline	Asymptomatic, QTc interval 0.48 – 0.49 s OR Increase in interval 0.03 – 0.05 s above baseline	Asymptomatic, QTc interval ≥ 0.50 s OR Increase in interval ≥ 0.06 s above baseline	Life-threatening consequences, e.g., Torsade de pointes or other associated serious ventricular dysrhythmia
	**Pediatric ≤ 16 years**	Asymptomatic, QTc interval 0.450 – 0.464 s	Asymptomatic, QTc interval 0.465 – 0.479 s	Asymptomatic, QTc interval ≥ 0.480 s	Life-threatening consequences, e.g., Torsade de pointes or other associated serious ventricular dysrhythmia
Thrombosis/embolism		NA	Deep vein thrombosis AND No intervention indicated (e.g., anticoagulation, lysis filter, invasive procedure)	Deep vein thrombosis AND Intervention indicated (e.g., anticoagulation, lysis filter, invasive procedure)	Embolic event (e.g., pulmonary embolism, life-threatening thrombus)
Vasovagal episode (associated with a procedure of any kind)		Present without loss of consciousness	Present with transient loss of consciousness	NA	NA
Ventricular dysfunction (congestive heart failure)		NA	Asymptomatic diagnostic finding AND intervention indicated	New onset with symptoms OR Worsening symptomatic congestive heart failure	Life-threatening congestive heart failure

CLINICAL				
**PARAMETER**	**GRADE 1 MILD**	**GRADE 2 MODERATE**	**GRADE 3 SEVERE**	**GRADE 4 POTENTIALLY LIFE-THREATENING**
**GASTROINTESTINAL**				
Anorexia	Loss of appetite without decreased oral intake	Loss of appetite associated with decreased oral intake without significant weight loss	Loss of appetite associated with significant weight loss	Life-threatening consequences OR Aggressive intervention indicated (e.g., tube feeding or total parenteral nutrition [TPN])
Ascites	Asymptomatic	Symptomatic AND Intervention indicated (e.g., diuretics or therapeutic paracentesis)	Symptomatic despite intervention	Life-threatening consequences

Basic Self-care Functions – Adult: Activities such as bathing, dressing, toileting, transfer/movement, continence, and feeding.

Basic Self-care Functions – Young Children: Activities that are age and culturally appropriate (e.g., feeding self with culturally appropriate eating implement).

Usual Social & Functional Activities – Adult: Adaptive tasks and desirable activities, such as going to work, shopping, cooking, use of transportation, pursuing a hobby, etc.

Usual Social & Functional Activities – Young Children: Activities that are age and culturally appropriate (e.g., social interactions, play activities, learning tasks, etc.)

CLINICAL				
PARAMETER	GRADE 1<br>MILD	GRADE 2<br>MODERATE	GRADE 3<br>SEVERE	GRADE 4<br>POTENTIALLY<br>LIFE-THREATENING
Cholecystitis	NA	Symptomatic AND Medical intervention indicated	Radiologic, endoscopic, or operative intervention indicated	Life-threatening consequences (e.g., sepsis or perforation)
Constipation	NA	Persistent constipation requiring regular use of dietary modifications, laxatives, or enemas	Obstipation with manual evacuation indicated	Life-threatening consequences (e.g., obstruction)
Diarrhea				
**Adult and Pediatric ≥ 1 year**	Transient or intermittent episodes of unformed stools OR Increase of ≤ 3 stools over baseline per 24-hour period	Persistent episodes of unformed to watery stools OR Increase of 4 – 6 stools over baseline per 24-hour period	Bloody diarrhea OR Increase of ≥ 7 stools per 24-hour period OR i.v. fluid replacement indicated	Life-threatening consequences (e.g., hypotensive shock)
**Pediatric < 1 year**	Liquid stools (more unformed than usual) but usual number of stools	Liquid stools with increased number of stools OR Mild dehydration	Liquid stools with moderate dehydration	Liquid stools resulting in severe dehydration with aggressive rehydration indicated OR Hypotensive shock
Dysphagia-Odynophagia	Symptomatic but able to eat usual diet	Symptoms causing altered dietary intake without medical intervention indicated	Symptoms causing severely altered dietary intake with medical intervention indicated	Life-threatening reduction in oral intake
Mucositis/stomatitis (clinical exam)<br>Indicate site (e.g., larynx, oral)<br>See Genitourinary for Vulvovaginitis<br>See also Dysphagia-Odynophagia and Proctitis	Erythema of the Mucosa	Patchy pseudomembranes or ulcerations	Confluent pseudomembranes or ulcerations OR Mucosal bleeding with minor trauma	Tissue necrosis OR Diffuse spontaneous mucosal bleeding OR Life-threatening consequences (e.g., aspiration, choking)

CLINICAL				
**PARAMETER**	**GRADE 1 MILD**	**GRADE 2 MODERATE**	**GRADE 3 SEVERE**	**GRADE 4 POTENTIALLY LIFE-THREATENING**
Nausea	Transient (< 24 hours) or intermittent nausea with no or minimal interference with oral intake	Persistent nausea resulting in decreased oral intake for 24 – 48 hours	Persistent nausea resulting in minimal oral intake for > 48 hours OR Aggressive rehydration indicated (e.g., i.v. fluids)	Life-threatening consequences (e.g., hypotensive shock)

Basic Self-care Functions – Adult: Activities such as bathing, dressing, toileting, transfer/movement, continence, and feeding.
Basic Self-care Functions – Young Children: Activities that are age and culturally appropriate (e.g., feeding self with culturally appropriate eating implement).
Usual Social & Functional Activities – Adult: Adaptive tasks and desirable activities, such as going to work, shopping, cooking, use of transportation, pursuing a hobby, etc.
Usual Social & Functional Activities – Young Children: Activities that are age and culturally appropriate (e.g., social interactions, play activities, learning tasks, etc.)

CLINICAL				
**PARAMETER**	**GRADE 1 MILD**	**GRADE 2 MODERATE**	**GRADE 3 SEVERE**	**GRADE 4 POTENTIALLY LIFE-THREATENING**
Pancreatitis	NA	Symptomatic AND Hospitalization not indicated (other than emergency room visit)	Symptomatic AND Hospitalization indicated (other than emergency room visit)	Life-threatening consequences (e.g., circulatory failure, hemorrhage, sepsis)
Proctitis (functional-symptomatic) Also see Mucositis/stomatitis for clinical exam	Rectal discomfort AND No intervention Indicated	Symptoms causing greater than minimal interference with usual social & functional activities OR Medical intervention indicated	Symptoms causing inability to perform usual social & functional activities OR Operative intervention indicated	Life-threatening consequences (e.g., perforation)
Vomiting	Transient or intermittent vomiting with no or minimal interference with oral intake	Frequent episodes of vomiting with no or mild dehydration	Persistent vomiting resulting in orthostatic hypotension OR Aggressive rehydration indicated (e.g., i.v. fluids)	Life-threatening consequences (e.g., hypotensive shock)
**NEUROLOGIC**				
Alteration in personality-behavior or in mood (e.g., agitation, anxiety, depression, mania, psychosis)	Alteration causing no or minimal interference with usual social & functional activities	Alteration causing greater than minimal interference with usual social & functional activities	Alteration causing inability to perform usual social & functional activities	Behavior potentially harmful to self or others (e.g., suicidal and homicidal ideation or attempt, acute psychosis) OR Causing inability to perform basic self-care functions
Altered Mental Status For Dementia, see Cognitive and behavioral/attentional disturbance (including dementia and attention deficit disorder)	Changes causing no or minimal interference with usual social & functional activities	Mild lethargy or somnolence causing greater than minimal interference with usual social & functional activities	Confusion, memory impairment, lethargy, or somnolence causing inability to perform usual social & functional activities	Delirium OR obtundation, OR coma

CLINICAL				
**PARAMETER**	**GRADE 1 MILD**	**GRADE 2 MODERATE**	**GRADE 3 SEVERE**	**GRADE 4 POTENTIALLY LIFE-THREATENING**
Ataxia	Asymptomatic ataxia detectable on exam OR Minimal ataxia causing no or minimal interference with usual social & functional activities	Symptomatic ataxia causing greater than minimal interference with usual social & functional activities	Symptomatic ataxia causing inability to perform usual social & functional activities	Disabling ataxia causing inability to perform basic self-care functions

Basic Self-care Functions – Adult: Activities such as bathing, dressing, toileting, transfer/movement, continence, and feeding.
Basic Self-care Functions – Young Children: Activities that are age and culturally appropriate (e.g., feeding self with culturally appropriate eating implement).
Usual Social & Functional Activities – Adult: Adaptive tasks and desirable activities, such as going to work, shopping, cooking, use of transportation, pursuing a hobby, etc.
Usual Social & Functional Activities – Young Children: Activities that are age and culturally appropriate (e.g., social interactions, play activities, learning tasks, etc.)

CLINICAL				
**PARAMETER**	**GRADE 1 MILD**	**GRADE 2 MODERATE**	**GRADE 3 SEVERE**	**GRADE 4 POTENTIALLY LIFE-THREATENING**
Cognitive and behavioral/attentional disturbance (including dementia and attention deficit disorder)	Disability causing no or minimal interference with usual social & functional activities OR Specialized resources not indicated	Disability causing greater than minimal interference with usual social & functional activities OR Specialized resources on part-time basis indicated	Disability causing inability to perform usual social & functional activities OR Specialized resources on a full-time basis indicated	Disability causing inability to perform basic self-care functions OR Institutionalization Indicated
CNS ischemia (acute)	NA	NA	Transient ischemic Attack	Cerebral vascular accident (CVA, stroke) with neurological deficit
Developmental delay **Pediatric ≤ 16 years**	Mild developmental delay, either motor or cognitive, as determined by comparison with a developmental screening tool appropriate for the setting	Moderate developmental delay, either motor or cognitive, as determined by comparison with a developmental screening tool appropriate for the setting	Severe developmental delay, either motor or cognitive, as determined by comparison with a developmental screening tool appropriate for the setting	Developmental regression, either motor or cognitive, as determined by comparison with a developmental screening tool appropriate for the setting
Headache	Symptoms causing no or minimal interference with usual social & functional activities	Symptoms causing greater than minimal interference with usual social & functional activities	Symptoms causing inability to perform usual social & functional activities	Symptoms causing inability to perform basic self-care functions OR Hospitalization indicated (other than emergency room visit) OR Headache with significant impairment of alertness or other neurologic function
Insomnia	NA	Difficulty sleeping causing greater than minimal interference with usual social & functional activities	Difficulty sleeping causing inability to perform usual social & functional activities	Disabling insomnia causing inability to perform basic self-care functions

CLINICAL				
**PARAMETER**	**GRADE 1 MILD**	**GRADE 2 MODERATE**	**GRADE 3 SEVERE**	**GRADE 4 POTENTIALLY LIFE-THREATENING**
Neuromuscular weakness (including myopathy & neuropathy)	Asymptomatic with decreased strength on exam OR Minimal muscle weakness causing no or minimal interference with usual social & functional activities	Muscle weakness causing greater than minimal interference with usual social & functional activities	Muscle weakness causing inability to perform usual social & functional activities	Disabling muscle weakness causing inability to perform basic self-care functions OR Respiratory muscle weakness impairing ventilation

Basic Self-care Functions – Adult: Activities such as bathing, dressing, toileting, transfer/movement, continence, and feeding.
Basic Self-care Functions – Young Children: Activities that are age and culturally appropriate (e.g., feeding self with culturally appropriate eating implement).
Usual Social & Functional Activities – Adult: Adaptive tasks and desirable activities, such as going to work, shopping, cooking, use of transportation, pursuing a hobby, etc.
Usual Social & Functional Activities – Young Children: Activities that are age and culturally appropriate (e.g., social interactions, play activities, learning tasks, etc.)

CLINICAL				
**PARAMETER**	**GRADE 1 MILD**	**GRADE 2 MODERATE**	**GRADE 3 SEVERE**	**GRADE 4 POTENTIALLY LIFE-THREATENING**
Neurosensory alteration (including paresthesia and painful neuropathy)	Asymptomatic with sensory alteration on exam or minimal paresthesia causing no or minimal interference with usual social & functional activities	Sensory alteration or paresthesia causing greater than minimal interference with usual social & functional activities	Sensory alteration or paresthesia causing inability to perform usual social & functional activities	Disabling sensory alteration or paresthesia causing inability to perform basic self-care functions
Seizure: (new onset) **Adult ≥ 18 years** See also Seizure: (known pre-existing seizure disorder)	NA	1 seizure	2 – 4 seizures	Seizures of any kind which are prolonged, repetitive (e.g., status epilepticus), or difficult to control (e.g., refractory epilepsy)
Seizure: (known pre-existing seizure disorder) **Adult ≥ 18 years** For worsening of existing epilepsy the grades should be based on an increase from previous level of control to any of these levels.	NA	Increased frequency of pre-existing seizures (nonrepetitive) without change in seizure character OR Infrequent breakthrough seizures while on stable medication in a previously controlled seizure disorder	Change in seizure character from baseline either in duration or quality (e.g., severity or focality)	Seizures of any kind which are prolonged, repetitive (e.g., status epilepticus), or difficult to control (e.g., refractory epilepsy)
Seizure **Pediatric < 18 years**	Seizure, generalized onset with or without secondary generalization, lasting < 5 minutes with < 24 hours post-ictal state	Seizure, generalized onset with or without secondary generalization, lasting 5 – 20 minutes with < 24 hours post-ictal state	Seizure, generalized onset with or without secondary generalization, lasting > 20 minutes	Seizure, generalized onset with or without secondary generalization, requiring intubation and sedation
Syncope (not associated with a procedure)	NA	Present	NA	NA

CLINICAL				
**PARAMETER**	**GRADE 1 MILD**	**GRADE 2 MODERATE**	**GRADE 3 SEVERE**	**GRADE 4 POTENTIALLY LIFE-THREATENING**
Vertigo	Vertigo causing no or minimal interference with usual social & functional activities	Vertigo causing greater than minimal interference with usual social & functional activities	Vertigo causing inability to perform usual social & functional activities	Disabling vertigo causing inability to perform basic self-care functions

Basic Self-care Functions – Adult: Activities such as bathing, dressing, toileting, transfer/movement, continence, and feeding.

Basic Self-care Functions – Young Children: Activities that are age and culturally appropriate (e.g., feeding self with culturally appropriate eating implement).

Usual Social & Functional Activities – Adult: Adaptive tasks and desirable activities, such as going to work, shopping, cooking, use of transportation, pursuing a hobby, etc.

Usual Social & Functional Activities – Young Children: Activities that are age and culturally appropriate (e.g., social interactions, play activities, learning tasks, etc.)

CLINICAL					
**PARAMETER**		**GRADE 1 MILD**	**GRADE 2 MODERATE**	**GRADE 3 SEVERE**	**GRADE 4 POTENTIALLY LIFE-THREATENING**
**RESPIRATORY**					
Bronchospasm (acute)		FEV1 or peak flow reduced to 70%-80%	FEV1 or peak flow 50% – 69%	FEV1 or peak flow 25% – 49%	Cyanosis OR FEV1 or peak flow < 25% OR Intubation
Dyspnea or respiratory distress					
	**Adult ≥ 14 years**	Dyspnea on exertion with no or minimal interference with usual social & functional activities	Dyspnea on exertion causing greater than minimal interference with usual social & functional activities	Dyspnea at rest causing inability to perform usual social & functional activities	Respiratory failure with ventilatory support indicated
	**Pediatric < 14 years**	Wheezing OR minimal increase in respiratory rate for age	Nasal flaring OR Intercostal retractions OR Pulse oximetry 90% – 95%	Dyspnea at rest causing inability to perform usual social & functional activities OR Pulse oximetry < 90%	Respiratory failure with ventilatory support indicated
**MUSCULOSKELETAL**					
Arthralgia See also Arthritis		Joint pain causing no or minimal interference with usual social & functional activities	Joint pain causing greater than minimal interference with usual social & functional activities	Joint pain causing inability to perform usual social & functional activities	Disabling joint pain causing inability to perform basic self-care functions
Arthritis See also Arthralgia		Stiffness or joint swelling causing no or minimal interference with usual social & functional activities	Stiffness or joint swelling causing greater than minimal interference with usual social & functional activities	Stiffness or joint swelling causing inability to perform usual social & functional activities	Disabling joint stiffness or swelling causing inability to perform basic self-care functions
Bone Mineral Loss					
	**Adult ≥ 21 years**	BMD t-score -2.5 to -1.0	BMD t-score < -2.5	Pathological fracture (including loss of vertebral height)	Pathologic fracture causing life-threatening consequences
	**Pediatric < 21 years**	BMD z-score -2.5 to -1.0	BMD z-score < -2.5	Pathological fracture (including loss of vertebral height)	Pathologic fracture causing life-threatening consequences

Basic Self-care Functions – Adult: Activities such as bathing, dressing, toileting, transfer/movement, continence, and feeding.
Basic Self-care Functions – Young Children: Activities that are age and culturally appropriate (e.g., feeding self with culturally appropriate eating implement).
Usual Social & Functional Activities – Adult: Adaptive tasks and desirable activities, such as going to work, shopping, cooking, use of transportation, pursuing a hobby, etc.
Usual Social & Functional Activities – Young Children: Activities that are age and culturally appropriate (e.g., social interactions, play activities, learning tasks, etc.)

CLINICAL				
**PARAMETER**	**GRADE 1<br>MILD**	**GRADE 2<br>MODERATE**	**GRADE 3<br>SEVERE**	**GRADE 4<br>POTENTIALLY<br>LIFE-THREATENING**
Myalgia (noninjection site)	Muscle pain causing no or minimal interference with usual social & functional activities	Muscle pain causing greater than minimal interference with usual social & functional activities	Muscle pain causing inability to perform usual social & functional activities	Disabling muscle pain causing inability to perform basic self-care functions
Osteonecrosis	NA	Asymptomatic with radiographic findings AND No operative intervention indicated	Symptomatic bone pain with radiographic findings OR Operative intervention indicated	Disabling bone pain with radiographic findings causing inability to perform basic self-care functions
**GENITOURINARY**				
Cervicitis (<u>symptoms</u>) (For use in studies evaluating topical study agents)<br>For other cervicitis see Infection: Infection (any other than HIV infection)	Symptoms causing no or minimal interference with usual social & functional activities	Symptoms causing greater than minimal interference with usual social & functional activities	Symptoms causing inability to perform usual social & functional activities	Symptoms causing inability to perform basic self-care functions
Cervicitis (<u>clinical exam</u>) (For use in studies evaluating topical study agents)<br>For other cervicitis, see Infection: Infection (any other than HIV infection)	Minimal cervical abnormalities on examination (erythema, mucopurulent discharge, or friability) OR Epithelial disruption < 25% of total surface	Moderate cervical abnormalities on examination (erythema, mucopurulent discharge, or friability) OR Epithelial disruption of 25% – 49% total surface	Severe cervical abnormalities on examination (erythema, mucopurulent discharge, or friability) OR Epithelial disruption 50% – 75% total surface	Epithelial disruption > 75% total surface

CLINICAL				
**PARAMETER**	**GRADE 1 MILD**	**GRADE 2 MODERATE**	**GRADE 3 SEVERE**	**GRADE 4 POTENTIALLY LIFE-THREATENING**
Intermenstrual bleeding (IMB)	Spotting observed by participant OR Minimal blood observed during clinical or colposcopic examination	Inter-menstrual bleeding not greater in duration or amount than usual menstrual cycle	Inter-menstrual bleeding greater in duration or amount than usual menstrual cycle	Hemorrhage with life-threatening hypotension OR Operative intervention indicated
Urinary tract obstruction (e.g., stone)	NA	Signs or symptoms of urinary tract obstruction without hydronephrosis or renal dysfunction	Signs or symptoms of urinary tract obstruction with hydronephrosis or renal dysfunction	Obstruction causing life-threatening Consequences

Basic Self-care Functions – Adult: Activities such as bathing, dressing, toileting, transfer/movement, continence, and feeding.
Basic Self-care Functions – Young Children: Activities that are age and culturally appropriate (e.g., feeding self with culturally appropriate eating implement).
Usual Social & Functional Activities – Adult: Adaptive tasks and desirable activities, such as going to work, shopping, cooking, use of transportation, pursuing a hobby, etc.
Usual Social & Functional Activities – Young Children: Activities that are age and culturally appropriate (e.g., social interactions, play activities, learning tasks, etc.)

CLINICAL				
**PARAMETER**	**GRADE 1 MILD**	**GRADE 2 MODERATE**	**GRADE 3 SEVERE**	**GRADE 4 POTENTIALLY LIFE-THREATENING**
Vulvovaginitis (symptoms) (Use in studies evaluating topical study agents) For other vulvovaginitis see Infection: Infection (any other than HIV infection)	Symptoms causing no or minimal interference with usual social & functional activities	Symptoms causing greater than minimal interference with usual social & functional activities	Symptoms causing inability to perform usual social & functional activities	Symptoms causing inability to perform basic self-care functions
Vulvovaginitis (clinical exam) (Use in studies evaluating topical study agents) For other vulvovaginitis see Infection: Infection (any other than HIV infection)	Minimal vaginal abnormalities on examination OR Epithelial disruption < 25% of total surface	Moderate vaginal abnormalities on examination OR Epithelial disruption of 25% – 49% total surface	Severe vaginal abnormalities on examination OR Epithelial disruption 50% – 75% total surface	Vaginal perforation OR Epithelial disruption > 75% total surface
**OCULAR/VISUAL**				
Uveitis	Asymptomatic but detectable on exam	Symptomatic anterior uveitis OR Medical intervention indicated	Posterior or pan-uveitis OR Operative intervention indicated	Disabling visual loss in affected eye(s)
Visual changes (from baseline)	Visual changes causing no or minimal interference with usual social & functional activities	Visual changes causing greater than minimal interference with usual social & functional activities	Visual changes causing inability to perform usual social & functional activities	Disabling visual loss in affected eye(s)
**ENDOCRINE/METABOLIC**				
Abnormal fat accumulation (e.g., back of neck, breasts, abdomen)	Detectable by study participant (or by caregiver for young children and disabled adults)	Detectable on physical exam by health care provider	Disfiguring OR Obvious changes on casual visual inspection	NA

Basic Self-care Functions – Adult: Activities such as bathing, dressing, toileting, transfer/movement, continence, and feeding.
Basic Self-care Functions – Young Children: Activities that are age and culturally appropriate (e.g., feeding self with culturally appropriate eating implement).
Usual Social & Functional Activities – Adult: Adaptive tasks and desirable activities, such as going to work, shopping, cooking, use of transportation, pursuing a hobby, etc.
Usual Social & Functional Activities – Young Children: Activities that are age and culturally appropriate (e.g., social interactions, play activities, learning tasks, etc.)

CLINICAL				
**PARAMETER**	**GRADE 1 MILD**	**GRADE 2 MODERATE**	**GRADE 3 SEVERE**	**GRADE 4 POTENTIALLY LIFE-THREATENING**
Diabetes mellitus	NA	New onset without need to initiate medication OR Modification of current medications to regain glucose control	New onset with initiation of medication indicated OR Diabetes uncontrolled despite treatment modification	Life-threatening consequences (e.g., ketoacidosis, hyperosmolar nonketotic coma)
Gynecomastia	Detectable by study participant or caregiver (for young children and disabled adults)	Detectable on physical exam by health care provider	Disfiguring OR Obvious on casual visual inspection	NA
Hyperthyroidism	Asymptomatic	Symptomatic causing greater than minimal interference with usual social & functional activities OR Thyroid suppression therapy indicated	Symptoms causing inability to perform usual social & functional activities OR Uncontrolled despite treatment modification	Life-threatening consequences (e.g., thyroid storm)
Hypothyroidism	Asymptomatic	Symptomatic causing greater than minimal interference with usual social & functional activities OR Thyroid replacement therapy indicated	Symptoms causing inability to perform usual social & functional activities OR Uncontrolled despite treatment modification	Life-threatening consequences (e.g., myxedema coma)
Lipoatrophy (e.g., fat loss from the face, extremities, buttocks)	Detectable by study participant (or by caregiver for young children and disabled adults)	Detectable on physical exam by health care provider	Disfiguring OR Obvious on casual visual inspection	NA

Basic Self-care Functions – Adult: Activities such as bathing, dressing, toileting, transfer/movement, continence, and feeding.

Basic Self-care Functions – Young Children: Activities that are age and culturally appropriate (e.g., feeding self with culturally appropriate eating implement).

Usual Social & Functional Activities – Adult: Adaptive tasks and desirable activities, such as going to work, shopping, cooking, use of transportation, pursuing a hobby, etc.

Usual Social & Functional Activities – Young Children: Activities that are age and culturally appropriate (e.g., social interactions, play activities, learning tasks, etc.)

LABORATORY				
**PARAMETER**	**GRADE 1 MILD**	**GRADE 2 MODERATE**	**GRADE 3 SEVERE**	**GRADE 4 POTENTIALLY LIFE-THREATENING**
**HEMATOLOGY** *Standard International Units are listed in italics*				
Absolute CD4+ count **Adult and Pediatric > 13 years** (HIV negative only)	300 – 400/mm³<br>*300 – 400/µL*	200 – 299/mm³<br>*200 – 299/µL*	100 – 199/mm³<br>*100 – 199/µL*	< 100/mm³<br>*< 100/µL*
Absolute lymphocyte count **Adult and Pediatric > 13 years** (HIV negative only)	600 – 650/mm³<br>*0.600×10^9 – 0.650×10^9/L*	500 – 599/mm³<br>*0.500×10^9 – 0.599×10^9/L*	350 – 499/mm³<br>*0.350×10^9 – 0.499×10^9/L*	< 350/mm³<br>*< 0.350×10^9/L*
Absolute neutrophil count (ANC)				
**Adult and Pediatric > 7 days**	1,000 – 1,300/mm³<br>*1.000×10^9 – 1.300×10^9/L*	750 – 999/mm³<br>*0.750×10^9 – 0.999×10^9/L*	500 – 749/mm³<br>*0.500×10^9 – 0.749×10^9/L*	< 500/mm³<br>*< 0.500×10^9/L*
**Infant[a,b] 2 – ≤ 7 days**	1,250 – 1,500/mm³<br>*1.250×10^9 – 1.500×10^9/L*	1,000 – 1,249/mm³<br>*1.000×10^9 – 1.249×10^9/L*	750 – 999/mm³<br>*0.750×10^9 – 0.999×10^9/L*	< 750/mm³<br>*< 0.750×10^9/L*
**Infant[a,b] 1 day**	4,000 – 5,000/mm³<br>*4.000×10^9 – 5.000×10^9/L*	3,000 – 3,999/mm³<br>*3.000×10^9 – 3.999×10^9/L*	1,500 – 2,999/mm³<br>*1.500×10^9 – 2.999×10^9/L*	< 1,500/mm³<br>*< 1.500×10^9/L*
Fibrinogen, decreased[c]	100 – 200 mg/dL<br>*1.00 – 2.00 g/L*<br>OR<br>≥ 0.75 to < 1.00 x LLN	75 – 99 mg/dL<br>*0.75 – 0.99 g/L*<br>OR<br>≥ 0.50 to < 0.75 x LLN	50 – 74 mg/dL<br>*0.50 – 0.74 g/L*<br>OR<br>≥ 0.25 to < 0.50 x LLN	< 50 mg/dL<br>*< 0.50 g/L*<br>OR<br>< 0.25 x LLN<br>OR Associated with gross bleeding
Hemoglobin (Hgb)[d]				
**Adult and Pediatric ≥ 57 days** (HIV positive only)	8.5 – 10.0 g/dL<br>*5.2 – 6.1 mmol/L*	7.5 – 8.4 g/dL<br>*4.6 – 5.1 mmol/L*	6.5 – 7.4 g/dL<br>*3.9 – 4.5 mmol/L*	< 6.5 g/dL<br>*< 3.9 mmol/L*

LABORATORY					
PARAMETER		GRADE 1<br>MILD	GRADE 2<br>MODERATE	GRADE 3<br>SEVERE	GRADE 4<br>POTENTIALLY<br>LIFE-THREATENING
	**Adult and Pediatric ≥ 57 days** (HIV negative only)	10.0 – 10.9 g/dL<br>*6.1 – 6.6 mmol/L*<br>OR<br>Any decrease<br>2.5 – 3.4 g/dL<br>*1.5 – 2.0 mmol/L*	9.0 – 9.9 g/dL<br>*5.5 – 6.0 mmol/L*<br>OR<br>Any decrease<br>3.5 – 4.4 g/dL<br>*2.1 – 2.6 mmol/L*	7.0 – 8.9 g/dL<br>*4.2 – 5.4 mmol/L*<br>OR<br>Any decrease<br>≥ 4.5 g/dL<br>*≥ 2.7 mmol/L*	< 7.0 g/dL<br>*< 4.2 mmol/L*
	**Infant[a,b] 36 – 56 days** (HIV positive or negative)	8.5 – 9.4 g/dL<br>*5.2 – 5.7 mmol/L*	7.0 – 8.4 g/dL<br>*4.2 – 5.1 mmol/L*	6.0 – 6.9 g/dL<br>*3.6 – 4.1 mmol/L*	< 6.0 g/dL<br>*< 3.6 mmol/L*

a Values are for term infants.
b Use age and sex appropriate values (e.g., bilirubin), including preterm infants.
c Revised by Tibotec.
d Revised by Tibotec; monomer conversion factor used for conversion from g/dL to mmol/L

LABORATORY				
**PARAMETER**	**GRADE 1 MILD**	**GRADE 2 MODERATE**	**GRADE 3 SEVERE**	**GRADE 4 POTENTIALLY LIFE-THREATENING**
**Infant**[a,b] **22 – 35 days** (HIV positive or negative)	9.5 – 10.5 g/dL *5.8 – 6.4 mmol/L*	8.0 – 9.4 g/dL *4.8 – 5.7 mmol/L*	7.0 – 7.9 g/dL *4.2 – 4.7 mmol/L*	< 7.00 g/dL *< 4.2 mmol/L*
**Infant**[a,b] **1 – 21 days** (HIV positive or negative)	12.0 – 13.0 g/dL *7.3 – 7.9 mmol/L*	10.0 – 11.9 g/dL *6.1 – 7.2 mmol/L*	9.0 – 9.9 g/dL *5.5 – 6.0 mmol/L*	< 9.0 g/dL *< 5.5 mmol/L*
International normalized ratio of prothrombin time (INR)[c]	≥ 1.1 to ≤ 1.5 x ULN	> 1.5 to ≤ 2.0 x ULN	> 2.0 to ≤ 3.0 x ULN	> 3.0 x ULN
Methemoglobin	5.0% – 10.0%	10.1% – 15.0%	15.1% – 20.0%	> 20.0%
Prothrombin time (PT)[c,d]	≥ 1.1 to ≤ 1.25 x ULN	> 1.25 to ≤ 1.50 x ULN	> 1.50 to ≤ 3.00 x ULN	> 3.00 x ULN
Partial thromboplastin time (PTT)[c]	≥ 1.1 to ≤ 1.66 x ULN	> 1.66 to ≤ 2.33 x ULN	> 2.33 to ≤ 3.00 x ULN	> 3.00 x ULN
Platelets, decreased	100,000 – 124,999/mm³ *100.000 x 10^9 – 124.999 x 10^9/L*	50,000 – 99,999/mm³ *50.000 x 10^9– 99.999 x 10^9/L*	25,000 – 49,999/mm³ *25.000 x 10^9– 49.999 x 10^9/L*	< 25,000/mm³ *< 25.000 x 10^9/L*
WBC, decreased	2,000 – 2,500/mm³ *2.000 x 10^9 – 2.500 x 10^9/L*	1,500 – 1,999/mm³ *1.500 x 10^9– 1.999 x 10^9/L*	1,000 – 1,499/mm³ *1.000 x 10^9– 1.499 x 10^9/L*	< 1,000/mm³ *< 1.000 x 10^9/L*
**CHEMISTRIES** ***Standard International Units are listed in italics***				
Acidosis	NA	pH < normal, but ≥ 7.3	pH < 7.3 without life-threatening consequences	pH < 7.3 with life-threatening consequences
Albumin, serum, low	3.0 g/dL – < LLN *30 g/L – < LLN*	2.0 – 2.9 g/dL *20 – 29 g/L*	< 2.0 g/dL *< 20 g/L*	NA
Alkaline phosphatase[c]	≥ 1.25 to ≤ 2.5 x ULN[b]	> 2.5 to ≤ 5.0 x ULN[b]	> 5.0 to ≤ 10.0 x ULN[b]	> 10.0 x ULN[b]
Alkalosis	NA	pH > normal, but ≤ 7.5	pH > 7.5 without life-threatening consequences	pH > 7.5 with life-threatening consequences
ALT (SGPT)[c]	≥ 1.25 to ≤ 2.5 x ULN	> 2.5 to ≤ 5.0 x ULN	> 5.0 to ≤ 10.0 x ULN	> 10.0 x ULN
AST (SGOT)[c]	≥ 1.25 to ≤ 2.5 x ULN	> 2.5 to ≤ 5.0 x ULN	> 5.0 to ≤ 10.0 x ULN	> 10.0 x ULN
Bicarbonate, serum, low	16.0 mEq/L – < LLN *16.0 mmol/L – < LLN*	11.0 – 15.9 mEq/L *11.0 – 15.9 mmol/L*	8.0 – 10.9 mEq/L *8.0 – 10.9 mmol/L*	< 8.0 mEq/L *< 8.0 mmol/L*

[a] Values are for term infants.

[b] Use age- and sex-appropriate values (e.g., bilirubin), including preterm infants.

[c] Revised by Tibotec.

[d] If the local laboratory is reporting PT as percentage, only INR value will be considered for reporting PT related abnormalities and AEs.

LABORATORY					
PARAMETER		GRADE 1 MILD	GRADE 2 MODERATE	GRADE 3 SEVERE	GRADE 4 POTENTIALLY LIFE-THREATENING
Bilirubin (Total)[a]					
	**Adult and Pediatric >14 days**	≥ 1.1 to ≤ 1.5 x ULN	> 1.5 to ≤ 2.5 x ULN	> 2.5 to ≤ 5.0 x ULN	> 5.0 x ULN
	**Infant[b,c] ≤ 14 days** (non-hemolytic)	NA	20.0 – 25.0 mg/dL<br>*342 – 428 µmol/L*	25.1 – 30.0 mg/dL<br>*429 – 513 µmol/L*	> 30.0 mg/dL<br>*> 513.0 µmol/L*
	**Infant [b,c] ≤ 14 days** (hemolytic)	NA	NA	20.0 – 25.0 mg/dL<br>*342 – 428 µmol/L*	> 25.0 mg/dL<br>*> 428 µmol/L*
Calcium, serum, high (corrected for albumin)					
	**Adult and Pediatric ≥ 7 days**	10.6 – 11.5 mg/dL<br>*2.65 – 2.88 mmol/L*	11.6 – 12.5 mg/dL<br>*2.89 – 3.13 mmol/L*	12.6 – 13.5 mg/dL<br>*3.14 – 3.38 mmol/L*	> 13.5 mg/dL<br>*> 3.38 mmol/L*
	**Infant [b,c] < 7 days**	11.5 – 12.4 mg/dL<br>*2.88 – 3.10 mmol/L*	12.5 – 12.9 mg/dL<br>*3.11 – 3.23 mmol/L*	13.0 – 13.5 mg/dL<br>*3.24 – 3.38 mmol/L*	> 13.5 mg/dL<br>*> 3.38 mmol/L*
Calcium, serum, low (corrected for albumin)					
	**Adult and Pediatric ≥ 7 days**	7.8 – 8.4 mg/dL<br>*1.95 – 2.10 mmol/L*	7.0 – 7.7 mg/dL<br>*1.75 – 1.94 mmol/L*	6.1 – 6.9 mg/dL<br>*1.53 – 1.74 mmol/L*	< 6.1 mg/dL<br>*< 1.53 mmol/L*
	**Infant [b,c] < 7 days**	6.5 – 7.5 mg/dL<br>*1.63 – 1.88 mmol/L*	6.0 – 6.4 mg/dL<br>*1.50 – 1.62 mmol/L*	5.50 – 5.90 mg/dL<br>*1.38 – 1.49 mmol/L*	< 5.50 mg/dL<br>*< 1.38 mmol/L*
Cardiac troponin I (cTnI)		NA	NA	NA	Levels consistent with myocardial infarction or unstable angina as defined by the manufacturer
Cardiac troponin T (cTnT)		NA	NA	NA	≥ 0.20 ng/mL OR<br>Levels consistent with myocardial infarction or unstable angina as defined by the manufacturer
Cholesterol (fasting)					
	**Adult ≥ 18 years**	200 – 239 mg/dL<br>*5.18 – 6.19 mmol/L*	240 – 300 mg/dL<br>*6.20 – 7.77 mmol/L*	> 300 mg/dL<br>*> 7.77 mmol/L*	NA
	**Pediatric < 18 years**	170 – 199 mg/dL<br>*4.40 – 5.15 mmol/L*	200 – 300 mg/dL<br>*5.16 – 7.77 mmol/L*	> 300 mg/dL<br>*> 7.77 mmol/L*	NA
Creatine kinase[a]		≥ 3.0 to ≤ 5.9 x ULN[c]	> 5.9 to ≤ 9.9 x ULN[c]	> 9.9 to ≤ 19.9 x ULN[c]	> 19.9 x ULN[c]

LABORATORY					
PARAMETER		GRADE 1<br>MILD	GRADE 2<br>MODERATE	GRADE 3<br>SEVERE	GRADE 4<br>POTENTIALLY<br>LIFE-THREATENING
Creatinine[a]		≥ 1.1 to ≤ 1.3 x ULN[c]	> 1.3 to ≤ 1.8 x ULN[c]	> 1.8 to ≤ 3.4 x ULN[c]	> 3.4 x ULN[c]
Glucose, serum, high					
	Nonfasting	116 – 160 mg/dL<br>*6.44 – 8.88 mmol/L*	161 – 250 mg/dL<br>*8.89 – 13.88 mmol/L*	251 – 500 mg/dL<br>*13.89 – 27.75 mmol/L*	> 500 mg/dL<br>*> 27.75 mmol/L*
	Fasting	110 – 125 mg/dL<br>*6.11 – 6.94 mmol/L*	126 – 250 mg/dL<br>*6.95 – 13.88 mmol/L*	251 – 500 mg/dL<br>*13.89 – 27.75 mmol/L*	> 500 mg/dL<br>*> 27.75 mmol/L*

[a] Revised by Tibotec.
[b] Values are for term infants.
[c] Use age- and sex-appropriate values (e.g., bilirubin), including preterm infants.

LABORATORY					
PARAMETER		GRADE 1 MILD	GRADE 2 MODERATE	GRADE 3 SEVERE	GRADE 4 POTENTIALLY LIFE-THREATENING
Glucose, serum, low					
	**Adult and Pediatric ≥ 1 month**	55 – 64 mg/dL<br>*3.05 – 3.55 mmol/L*	40 – 54 mg/dL<br>*2.22 – 3.00 mmol/L*	30 – 39 mg/dL<br>*1.67 – 2.21 mmol/L*	< 30 mg/dL<br>*< 1.67 mmol/L*
	**Infant[a,b] < 1 month**	50 – 54 mg/dL<br>*2.78 – 3.00 mmol/L*	40 – 49 mg/dL<br>*2.22 – 2.77 mmol/L*	30 – 39 mg/dL<br>*1.67 – 2.21 mmol/L*	< 30 mg/dL<br>*< 1.67 mmol/L*
Lactate		< 2.0 x ULN without acidosis	≥ 2.0 x ULN without acidosis	Increased lactate with pH < 7.3 without life-threatening consequences	Increased lactate with pH < 7.3 with life-threatening consequences
LDL cholesterol (fasting)					
	**Adult ≥ 18 years**	130 – 159 mg/dL<br>*3.37 – 4.12 mmol/L*	160 – 190 mg/dL<br>*4.13 – 4.90 mmol/L*	≥ 191 mg/dL<br>*≥ 4.91 mmol/L*	NA
	**Pediatric > 2 – < 18 Years**	110 – 129 mg/dL<br>*2.85 – 3.34 mmol/L*	130 – 189 mg/dL<br>*3.35 – 4.90 mmol/L*	≥ 190 mg/dL<br>*≥ 4.91 mmol/L*	NA
Lipase[c]		≥ 1.1 to ≤ 1.5 x ULN	> 1.5 to ≤ 3.0 x ULN	> 3.0 to ≤ 5.0 x ULN	> 5.0 x ULN
Magnesium, serum, low		1.2 – 1.4 mEq/L<br>*0.60 – 0.70 mmol/L*	0.9 – 1.1 mEq/L<br>*0.45 – 0.59 mmol/L*	0.6 – 0.8 mEq/L<br>*0.30 – 0.44 mmol/L*	< 0.60 mEq/L<br>*< 0.30 mmol/L*
Pancreatic amylase[c]		≥ 1.1 to ≤ 1.5 x ULN	> 1.5 to ≤ 2.0 x ULN	> 2.0 to ≤ 5.0 x ULN	> 5.0 x ULN
Phosphate, serum, low					
	**Adult and Pediatric > 14 years**	2.5 mg/dL – < LLN<br>*0.81 mmol/L – < LLN*	2.0 – 2.4 mg/dL<br>*0.65 – 0.80 mmol/L*	1.0 – 1.9 mg/dL<br>*0.32 – 0.64 mmol/L*	< 1.00 mg/dL<br>*< 0.32 mmol/L*
	**Pediatric 1 – 14 years**	3.0 – 3.5 mg/dL<br>*0.97 – 1.13 mmol/L*	2.5 – 2.9 mg/dL<br>*0.81 – 0.96 mmol/L*	1.5 – 2.4 mg/dL<br>*0.48 – 0.80 mmol/L*	< 1.50 mg/dL<br>*< 0.48 mmol/L*
	**Pediatric < 1 year**	3.5 – 4.5 mg/dL<br>*1.13 – 1.45 mmol/L*	2.5 – 3.4 mg/dL<br>*0.81 – 1.12 mmol/L*	1.5 – 2.4 mg/dL<br>*0.48 – 0.80 mmol/L*	< 1.50 mg/dL<br>*< 0.48 mmol/L*
Potassium, serum, high		5.6 – 6.0 mEq/L<br>*5.6 – 6.0 mmol/L*	6.1 – 6.5 mEq/L<br>*6.1 – 6.5 mmol/L*	6.6 – 7.0 mEq/L<br>*6.6 – 7.0 mmol/L*	> 7.0 mEq/L<br>*> 7.0 mmol/L*
Potassium, serum, low		3.0 – 3.4 mEq/L<br>*3.0 – 3.4 mmol/L*	2.5 – 2.9 mEq/L<br>*2.5 – 2.9 mmol/L*	2.0 – 2.4 mEq/L<br>*2.0 – 2.4 mmol/L*	< 2.0 mEq/L<br>*< 2.0 mmol/L*
Sodium, serum, high		146 – 150 mEq/L<br>*146 – 150 mmol/L*	151 – 154 mEq/L<br>*151 – 154 mmol/L*	155 – 159 mEq/L<br>*155 – 159 mmol/L*	≥ 160 mEq/L<br>*≥ 160 mmol/L*
Sodium, serum, low		130 – 135 mEq/L<br>*130 – 135 mmol/L*	125 – 129 mEq/L<br>*125 – 129 mmol/L*	121 – 124 mEq/L<br>*121 – 124 mmol/L*	≤ 120 mEq/L<br>*≤ 120 mmol/L*
Triglycerides (fasting)		NA	500 – 750 mg/dL<br>*5.65 – 8.48 mmol/L*	751 – 1,200 mg/dL<br>*8.49 – 13.56 mmol/L*	> 1,200 mg/dL<br>*> 13.56 mmol/L*
Uric acid		7.5 – 10.0 mg/dL<br>*0.45 – 0.59 mmol/L*	10.1 – 12.0 mg/dL<br>*0.60 – 0.71 mmol/L*	12.1 – 15.0 mg/dL<br>*0.72 – 0.89 mmol/L*	> 15.0 mg/dL<br>*> 0.89 mmol/L*

[a] Values are for term infants.

[b] Use age- and sex-appropriate values (e.g., bilirubin), including preterm infants.

[c] Revised by Tibotec.

LABORATORY					
PARAMETER		GRADE 1<br>MILD	GRADE 2<br>MODERATE	GRADE 3<br>SEVERE	GRADE 4<br>POTENTIALLY<br>LIFE-THREATENING
**URINALYSIS** ***Standard International Units are listed in italics***					
Hematuria (microscopic)		6 – 10 RBC/HPF	> 10 RBC/HPF	Gross, with or without clots OR with RBC casts	Transfusion indicated
Proteinuria, random collection		1 +	2 – 3 +	4 +	NA
Proteinuria, 24 hour collection					
	**Adult and Pediatric ≥ 10 years**	200 – 999 mg/24 h<br>*0.200 – 0.999 g/d*	1,000 – 1,999 mg/24 h<br>*1.000 – 1.999 g/d*	2,000 – 3,500 mg/24 h<br>*2.000 – 3.500 g/d*	> 3,500 mg/24 h<br>*> 3.500 g/d*
	**Pediatric > 3 months – < 10 years**	201 – 499 mg/m2/24 h<br>*0.201 – 0.499 g/d*	500 – 799 mg/m2/24 h<br>*0.500 – 0.799 g/d*	800 – 1,000 mg/m2/24 h<br>*0.800 – 1.000 g/d*	> 1,000 mg/ m2/24 h<br>*> 1.000 sg/d*

Appendix F. Child-Pugh Score

The score employs five clinical measures of liver disease. Each measure is scored 1-3, with 3 indicating most severe derangement.

Measure	1 Point	2 Points	3 Points
Total bilirubin, μmol/l (mg/dl)	< 34 (≤ 2)	34-50 (2-3)	> 50 (> 3)
Serum albumin, g/l	> 35	28-35	< 28
PT INR	< 1.7	1.71-2.30	> 2.30
Ascites	None	Mild	Moderate to Severe
Hepatic encephalopathy	None	Grade I-II (or suppressed with medication)	Grade III-IV (or refractory)

Chronic liver disease is classified into Child-Pugh class A to C, employing the added score from above.

Points	Class
5-6	A
7-9	B
10-15	C

Pugh RN, Murray-Lyon IM, Dawson JL, Pietroni MC, Williams R (1973). "Transection of the oesophagus for bleeding oesophageal varices". *The British journal of surgery* 60 (8): 646–9.
